# P(V)-Promoted Rh-Catalyzed Highly Regioselective Hydroformylation of Styrenes under Mild Conditions

**DOI:** 10.3390/molecules29092039

**Published:** 2024-04-28

**Authors:** Tong Ru, Yajiao Zhang, Qiuxiang Wei, Sheng Zuo, Zhenhua Jia, Fen-Er Chen

**Affiliations:** 1 Department of Medicinal Chemistry, School of Pharmacy, Fudan University, Shanghai 201203, China; 19111030025@fudan.edu.cn; 2 Engineering Center of Catalysis and Synthesis for Chiral Molecules, Department of Chemistry, Fudan University, Shanghai 200433, China; 200410027@fzu.edu.cn (Y.Z.); qiuxiangwei21@163.com (Q.W.);; 3 Shanghai Engineering Center of Industrial Asymmetric Catalysis for Chiral Drugs, Shanghai 200433, China; 4 College of Chemical Engineering, Fuzhou University, Fuzhou 350102, China

**Keywords:** hybrid phosphate, hydroformylation, styrene, branched selectivity

## Abstract

Hydroformylation of olefins is widely used in the chemical industry due to its versatility and the ability to produce valuable aldehydes with 100% atom economy. Herein, a hybrid phosphate promoter was found to efficiently promote rhodium-catalyzed hydroformylation of styrenes under remarkably mild conditions with high regioselectivities. Preliminary mechanistic studies revealed that the weak coordination between the Rhodium and the P=O double bond of this pentavalent phosphate likely induced exceptional reactivity and high ratios of branched aldehydes to linear products.

## 1. Introduction

Hydroformylation, popularly known as the “oxo” process, is an important transition-metal-catalyzed industrial process for the production of aldehydes from alkenes [1,2]. Annually, these oxo products have been produced on the scale of over 10 million tons worldwide [3,4,5]. BASF and ICI [6,7,8] developed the first- and second-generation catalytic systems using cobalt as the catalyst, respectively, under harsh conditions of 100–350 bar pressure and 100–200 °C. The third-generation process employs P-ligand-modified Rh catalysts, reducing the requirements of high pressure and temperature significantly, which are regarded as the most effective and selective catalysts for hydroformylation to date [9,10,11,12]. Despite advancement in recent years, several challenges still remain, including the high cost of using rhodium as a catalyst, suboptimal selectivity for some substrates, and a relatively high operational temperature of 85–130 °C [13].

**Scheme 1:**
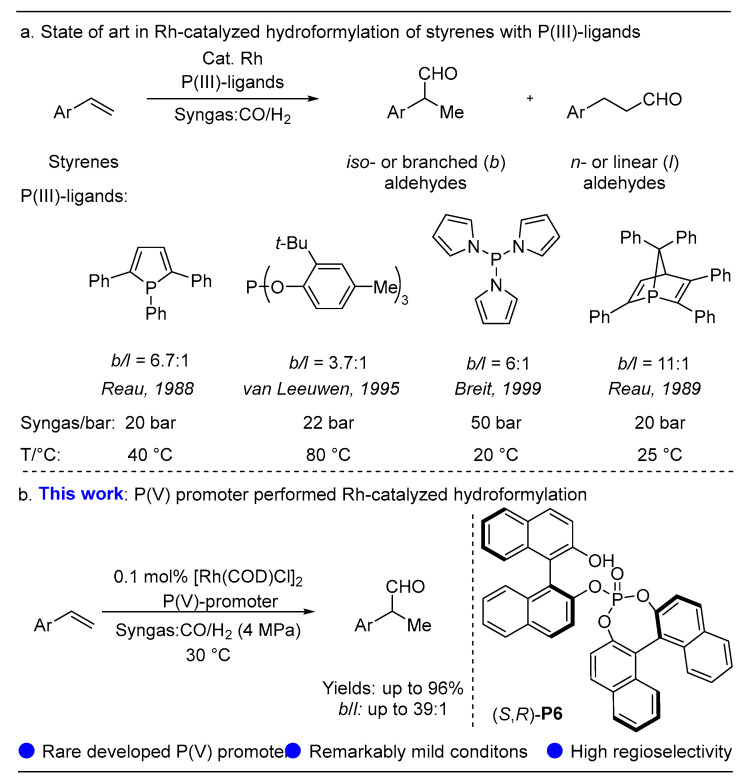
State of the art in Rh-catalyzed hydroformylation of styrenes with P(III)-ligands and this work: P(V) promoted Rh-catalyzed hydroformylation under mild conditions [14,15,16,17].

Mono-trivalent phosphine-ligands have been well-studied in the rhodium catalyzed hydroformylation of styrene to produce branched aldehyde selectively [14,15,16,17] (Scheme 1a). The ratios of branched to linear products was limited to 6.7:1 when using the phosphole ligand [14]. The tris(2, 4-di-*tert*-butylphenyl)phosphite ligand, widely utilized in the industry with a high *s*/*c* of 3500, exhibited poor regioselectivity (*b*/*l* = 3.7:1) [15]. When using trispyrrolylphosphine [16] and phosphanorbornadienes [17] as ligands, only moderate regioselectivities could be achieved. Furthermore, the possibility of phosphine oxidation to phosphine oxide during both preparation and reaction restricts the utility of P(III) ligands. Typically, the oxidation of triphenylphosphine (TPP) ligands to triphenylphosphine oxide (TPPO) decreases the electron density of the P atom, leading to lower coordination ability. However, pentavalent phosphate as an additive is usually stable and has been less extensively explored [18,19,20].

Macheetti and He et al. found that the carbon monoxide insertion into metal alkyl complexes was accelerated by P(V) [21,22]. Alper used a phosphine oxide ligand in rhodium-catalyzed hydroformylation of alkenes, achieving moderate yields and high regioselectivities [23]. Gusevskaya and co-workers reported cobalt-catalyzed hydroformylation with phosphine oxides under milder conditions [24]. The bidentate ligands, including phosphine, amino, and oxygen-phosphine oxide, have also been shown to promote the selectivity of transition-metal-catalyzed hydroformylation [25,26,27,28,29,30,31,32]. In our previous research, we developed the heterogeneous and homogeneous catalytic protocols of hydroformylation [33,34]. To continue our efforts in this field, in this paper, we present a novel hybrid phosphate that promoted rhodium-catalyzed hydroformylation of styrenes, exhibiting high reactivity and regioselectivity under remarkably mild conditions (Scheme 1b).

## 2. Results and Discussion

We initiated our study of Rh-catalyzed hydroformylation with styrene as the model substrate, 0.1 mol% [Rh(COD)Cl]_2_ as the catalyst, and 0.6 mol% trimethyl phosphate **P1** in toluene at 30 °C. In 24 h, only a 16% yield of branched aldehyde **2a** was obtained with moderate regioselectivity (*b*/*l* = 8.0:1, Table 1, entry 1). A notable increase in activity was achieved compared to the triphenyl phosphate **P2**, leading to a 40% yield of **2a** with 6.6:1 regioselectivity (entry 2), while the triphenylphosphine oxide **P3** was used, only yielding a trace amount of **2a** (entry 4). Moreover, the chiral phosphoric-acid-derived (*R*)-**P4** [35,36,37] was tested, resulting in a slowed reaction and the detection of only a trace amount of the desired product (entry 5). 

Inspired by the hybrid phosphine–phosphite developed by Takeya [38], we prepared a series of hybrid phosphates, **P5**–**P11**, and evaluated their efficiency for our desired Rh-catalyzed hydroformylation. When (*R*, *R*)-**P5** was used, high regioselectivity was observed, albeit the overall yield of **2a** was only 28% (entry 5). To our delight, (*S*, *R*)-**P6**, the diastereomer of **P5**, was employed, leading to a 96% yield of **2a** with excellent regioselectivity, and the ratio of *b*/*l* was up to 25.4:1 (entry 6,). However, no enantioselectivity was observed with **P6**, suggesting that the chiral skeleton may not affect asymmetric hydroformylation, which represents an important transformation to produce chiral aldehydes from simple alkenes [39,40,41,42,43,44]. Furthermore, we screened other hybrid chiral phosphates, such as the more sterically hindered (*S*, *R*)-**P7**, featuring a phenyl substituent at ortho-position of the hydroxyl group. Only a moderate outcome was achieved without any stereoselective control (entry 7). In addition, the employment of (*S*, *R*)-**P8** [45,46] yielded moderate results in terms of both yield and regioselectivity (entry 8). We then utilized more rigid chiral spiro backbone-based hybrid phosphates, **P9**–**P11**, recognized as a class of privileged ligands in asymmetric catalysis [47,48,49,50,51,52]; the reactivity and selectivity were not significantly improved (entries 9–11). 

**Figure 1:**
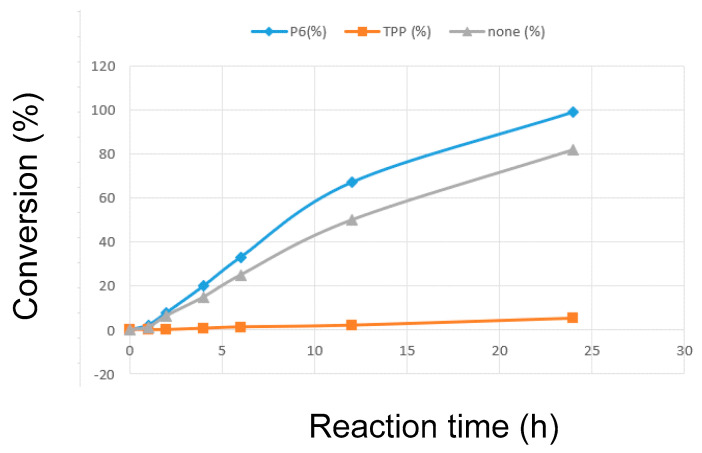
Catalytic performance of **P6** and TPP.

To verify the function of **P6**, we compared the performance of **P6** with triphenyphosphine (TPP) in Rh-catalyzed hydroformylation. As depicted in Figure 1, P6 was found to promote hydroformylation efficiently (blue line). However, the reaction proceeded slowly in the absence **P6**, resulting in 82% conversion of **1a** (gray line). Under the same conditions, TPP was utilized to slow the reaction, leading to lower conversion of styrene (orange line). 

**Figure 2:**
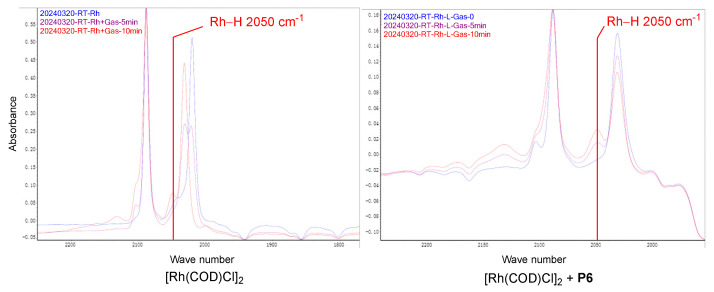
High-pressure (CO/H_2_ 1:1, 4.0 MPa) *in-situ* IR spectra.

Moreover, the acceleration effect may induce the rapid formation of active Rh-H species in the hydroformylation process [24]. As illustrated in Figure 2, we conducted in situ high-pressure (CO/H_2_ 1:1, 4.0 MPa) IR to detect the possible Rh−H species. Fortunately, we observed the Rh−H signal (2050 cm^−1^) within 10 min in the absence of **P6**. Notably, the use of **P6** shortened the time for the appearance of the same peak to 5 min.

Inspired by these results, we conducted a comprehensive study of various reaction parameters of regioselective Rh-catalyzed hydroformylation with phosphate **P6**, as detailed in Table 2. Initially, we noted that the yield of **2a** was not increased notably by simply elevating the reaction temperature from 30 to 50 °C. However, it resulted in a significant decrease in the *b*/*l* ratio (Table 2, entries 1–3). Our efforts then focused on the adjustment of the ratio of Rh/phosphate to optimize the conditions. Unfortunately, decreasing the ratio of Rh/L6 from 1:3 to 1:2 or increasing the ratio to 1:6 led to lower yields of **2a** with lower selectivity (entries 4 and 5, respectively). Further increasing the molar ratio to 1:8 resulted in less satisfactory results, suggesting that the optimal ratio of Rh/phosphate was 1:3 (entry 6). Of note, when we lowered the pressure of syngas (CO/H_2_ = 1:1) from 4.0 to 2.0 MPa, a trace amount of **2a** was detected (entries 7 and 8).

In addition, we examined the influence of various organic solvents. The transition to THF as the solvent slightly decreased the selectivity towards the branched product **2a** (entry 9), while the yield of **2a** and regioselectivity did not benefit from other solvents such as Et_2_O and DCM (entries 10 and 11). We changed different Rh catalysts in the desired hydroformylation reaction and found that the comparable results were obtained, when Rh(acac)(CO)_2_ was treated as the catalyst (entry 12) and RhCl_3_ significantly reduced the yield of **2a**, despite with good regioselectivity (entry 13).

**Scheme 2:**
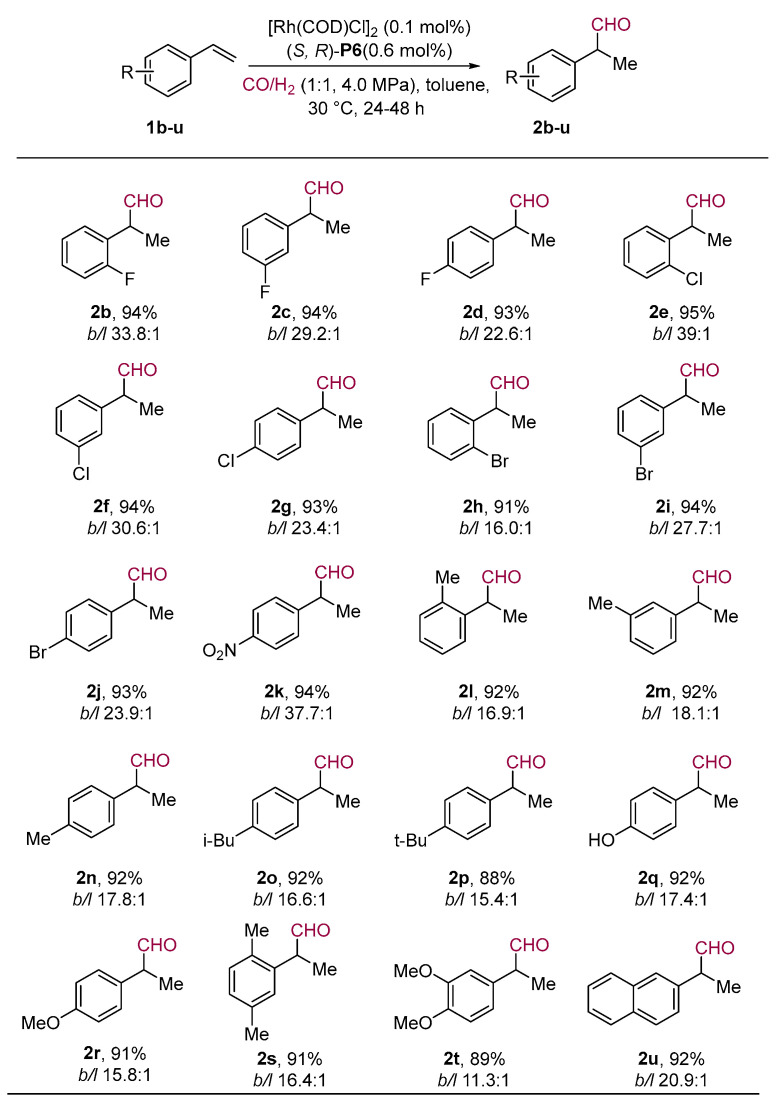
Substrate scope of Rh-(*S*, *R*)-**P6** catalyzed hydroformylation. Reaction conditions: substrates (3 mmol), [Rh(COD)Cl]_2_ (0.1 mol %), **P6** (0.6 mol %), toluene (25 mL), 30 °C, 24–48 h, syngas (CO/H_2_ = 1:1); all yields were isolated yields; *b*/*l* ratios were determined using GC.

With the optimal reaction conditions in hand, we next explored the scope of the Rh-catalyzed regioselective hydroformylation. This is depicted in Scheme 2. In general, styrene bearing diverse substituents at the para, meta, or ortho positions on the benzyl ring (**1b**–**1u**) were accommodated well to afford the corresponding products **2b**–**2u** in good-to-excellent yields (88–95%) with high regioselectivities (*b*/*l* = 11.3:1–39:1). Notably, mono-substituted styrenes with electron-withdrawing groups, including fluoro (**1b**–**1d**), chloro (**1e**–**1g**), bromo (**1h**–**1j**), and nitro groups (**1k**), showed comparatively higher reactivities and regioselectivities than those with electron-donating groups, such as methyl (**1l**–**1n**), *iso*-butyl (**1o**), *tert*-butyl (**1p**), hydroxyl (**1q**), and methoxyl (**1r**). Moreover, the side reactions, for instance, hydrogenation or hydrogenolysis, were not involved, and the possible by-products were not detected with GC analysis in **2b**–**2k**. Furthermore, disubstituted styrenes with dimethyl and dimethoxy groups also performed well under standard conditions, producing the target branched aldehydes **2s** and **2t** with commendable regioselectivities (*b*/*l* = 16.4:1–11.3:1) with 91% and 89% yields, respectively. Polyarene styrene derivative was successfully transformed into the aldehyde product **2u** with a 92% yield and a *b*/*l* ratio of 20.9:1.

**Scheme 3:**
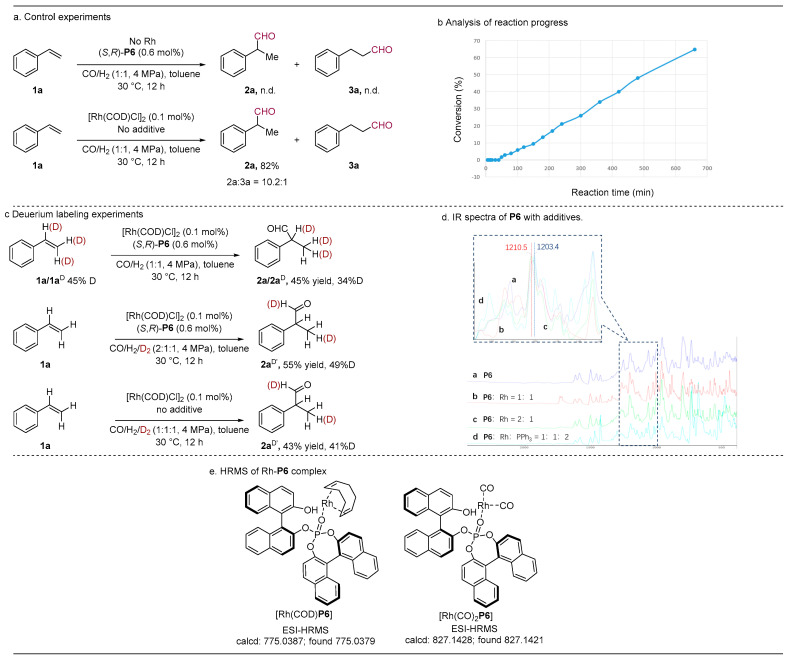
Mechanistic studies.

To elucidate the possible reaction mechanism, we conducted a series of control experiments as depicted in Scheme 3a. In the absence of rhodium catalyst, the aldehyde products were not detected and lower regioselectivity was observed without **P6**. When the reaction progress was monitored with offline analysis of time aliquots, we noticed a linear relationship between the reaction time and yield of **2a** (Scheme 3b). Furthermore, deuterium labeling experiments (Scheme 3c) were also carried out to reveal the possible role of the phosphate ligand **P6** in the catalytic cycle. First, the KIE of C1/C2 deuterated styrene was determined as 1.57. Then, we conducted the hydroformylation under a deuterium/hydrogen atmosphere and found that 49% deuterium labeled aldehyde **2a^D^’** was detected under standard conditions, but a relatively lower yield was obtained without **P6**. This disparity provided support for the accelerated hydrogenolysis of Rh-acyl species (VI) into Rh-H species (II) [53,54].

With the developed methods of continuous variation at a concentration of 0.6 mol%, we observed a liner correlation between the reactivity and the **P6**/TPP ratio (Table S1). The competing experiment showed that the coordination ability of **P6** is weaker than TPP. We also monitored the formation of Rh/**P6** complex in solution state via NMR analysis. Unfortunately, the chemical shift of ^31^P of **P6** did not show significant differences after stirring with [Rh(COD)Cl]_2_. In the IR spectra, a shift of the P=O stretching band from 1203.4 to 1210.5 cm^−1^ was observed (Scheme 3d). This shift indicated a possible weak coordination of the phosphine oxide group to the Rh. Moreover, we successfully detected the [Rh(COD)**P6**] and [Rh(CO)_2_**P6**] species, respectively, via ESI-HRMS by simply mixing [Rh(COD)Cl]_2_ with **P6** in toluene under a nitrogen and CO atmosphere (Scheme 3e).

**Scheme 4:**
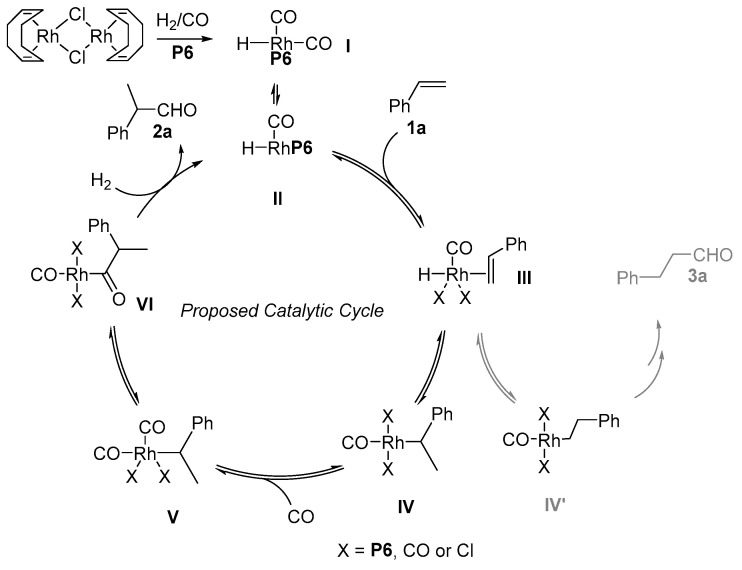
Proposed catalytic cycle.

Based on the precedent research and our experimental evidence, we proposed the mechanism of the regioselective Rh-catalyzed hydroformylation. As showed in Scheme 4, [Rh(COD)Cl]_2_ reacted with syngas to form rhodium complex (**I**) assisted by ligand, which was rapidly transformed into complex (**II**) via the release of one CO molecule. Subsequently, complex (**II**) coordinated with styrene to generate complex (**III**), with the hydride of rhodium complex (**III**) favoring attack on the C1 carbon atom of styrene over the C2 carbon atom, resulting in excellent regioselectivity. Following another coordination of CO and hydrolysis by H_2_, branched product **2a** was obtained and complex (**II**) was regenerated. 

## 3. Materials and Methods

All commercial regents were used directly without further purification and solvents were dried according to standard procedures. NMR spectra were recorded on a Bruker ADVANCE III (400 MHz) spectrometer. CDCl_3_ or DMSO-*d_6_* were the solvents used for the NMR analysis, with tetramethylsilane as the internal standard. Data are reported as follows: chemical shift [multiplicity (br = broad, s = singlet, d = doublet, t = triplet, m = multiplet), coupling constant(s) in Hertz, integration]. GC-MS analysis was carried out on Angilent 7820A GC system and Angilent 5977B MSD. HRMS were recorded on a Bruker micrOTOF spectrometer (ESI). IR spectra were carried out on a ThermoFisher NICOLET iS10 IR spectrometer.

### 3.1. Synthesis of the Phosphates

#### 3.1.1. Synthesis of (R)-**P4** [35,37,55,56]


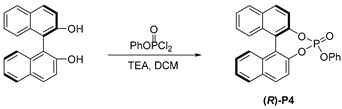


The (*R*)-(+)-1,1′-bi-2-naphthol (858 mg, 3 mmol) and Et_3_N (1.2 mL, 9 mmol) were dissolved in dry CH_2_Cl_2_ (10 mL), and then phenyl dichlorophosphate (756 mg, 3.6 mmol) was added dropwise under argon at 0 °C. The reaction was allowed to warm to room temperature and stirred overnight. After that, the solid was removed via filtration. The filtrate was concentrated and purified using flash column chromatography (CH_2_Cl_2_/PE) to obtain product **P4** as a white solid (1.14 g, 90% yield). ^1^H NMR (400 MHz, CDCl_3_) *δ* 7.96 (d, *J* = 8.9 Hz, 1H), 7.90 (d, *J* = 8.9 Hz, 1H), 7.85 (d, *J* = 8.2 Hz, 2H), 7.55 (d, *J* = 8.9 Hz, 1H), 7.37 (q, *J* = 3.0 Hz, 3H), 7.29–7.18 (m, 8H), 7.14–7.09 (m, 1H).

#### 3.1.2. Synthesis of (R, R)-**P5**


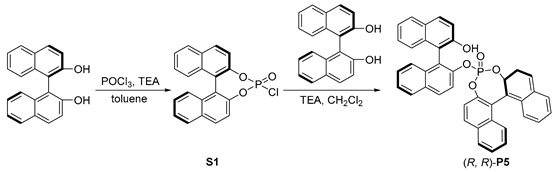


Preparation of chlorophosphonate **S1.**

The (*R*)-(+)-1,1′-bi-2-naphthol (5 g, 17.5 mmol) and Et_3_N (10 mL, 70 mmol) was dissolved in dry toluene (90 mL) and POCl_3_ (2.9 g, 19 mmol) was added dropwise under argon at 0 °C. The reaction was stirred at room temperature overnight. The solid was removed via filtration. The filtrate was concentrated and purified using flash column chromatography (EtOAc/PE) to obtain product **S1** as a white solid (4.99 g, 78% yield).

Preparation of the ligand (*R*, *R*)-**P5.**

Under a nitrogen atmosphere, to a solution of **S1** (3.0 g, 8 mmol) and (*R*)-(+)-1,1′-bi-2-naphthol (2.3 g, 8 mmol) in anhydrous CH_2_Cl_2_ (50 mL), Et_3_N (1.2 mL, 8 mmol) was added at 0 °C. The reaction was stirred at room temperature overnight. The solvent was removed under vacuum and the residue was purified using flash column chromatography (CH_2_Cl_2_/PE) to obtain product (*R*, *R*)-**P5** as a white solid (3.44 g, 70% yield). ^1^H NMR (400 MHz, DMSO-*d*_6_) *δ* 9.58 (s, 1H), 8.24 (d, *J* = 8.9 Hz, 1H), 8.18 (s, 1H), 8.06 (dt, *J* = 13.3, 7.1 Hz, 3H), 7.89 (d, *J* = 8.6 Hz, 2H), 7.82 (d, *J* = 9.7 Hz, 2H), 7.71 (d, *J* = 8.9 Hz, 1H), 7.50 (d, *J* = 7.6 Hz, 3H), 7.35–7.29 (m, 4H), 7.25 (d, *J* = 7.5 Hz, 1H), 7.19–7.12 (m, 4H), 6.98 (d, *J* = 8.3 Hz, 1H), 6.11 (d, *J* = 8.9 Hz, 1H); ^13^C NMR (100 MHz, DMSO-*d*_6_) *δ* 153.8, *δ* 146.9 (d, *J* = 11.7 Hz), 146.4 (d, *J* = 6.4 Hz), 146.0 (d, *J* = 8.4 Hz), 134.1, 133.8, 132.3, 132.0, 131.8 (d, *J* = 5.4 Hz), 131.7, 131.6 (d, *J* = 3.1 Hz), 130.1, 129.2, 128.7 (d, *J* = 9.9 Hz), 128.4, 127.8, 127.5 (d, *J* = 9.2 Hz), 127.0, 126.7, 126.6, 126.4, 126.2 (d, *J* = 5.0 Hz), 124.2, 124.14, 123.2, 121.2, 121.1 (d, *J* = 6.7 Hz), 121.0, 120.2, 120.1, 120.1 (d, *J* = 8.3 Hz), 119.6, 118.7, 113.2; ^31^P NMR (161 MHz, DMSO-*d*_6_) *δ* −2.78. HRMS (ESI) calcd for [C_40_H_35_NaO_5_P, M+Na]^+^: 639.1332, found: 639.1333.

#### 3.1.3. Synthesis of (S, R)-**P6**

The (*S*, *R*)-**P6** was prepared according to the (*R*, *R*)-**P5** procedure.

^1^H NMR (400 MHz, DMSO-*d*_6_) *δ* 9.75 (s, 1H), 8.19 (t, *J* = 8.7 Hz, 2H), 8.08 (t, *J* = 7.2 Hz, 2H), 7.98 (d, *J* = 8.3 Hz, 1H), 7.87 (t, *J* = 8.8 Hz, 2H), 7.81 (d, *J* = 9.0 Hz, 1H), 7.69 (d, *J* = 8.9 Hz, 1H), 7.51 (t, *J* = 7.8 Hz, 3H), 7.43 (d, *J* = 8.9 Hz, 1H), 7.34 (t, *J* = 8.8 Hz, 4H), 7.22–7.13 (m, 3H), 7.05 (d, *J* = 8.8 Hz, 2H), 6.78 (d, *J* = 8.5 Hz, 1H), 6.47 (d, *J* = 8.9 Hz, 1H); ^13^C NMR (100 MHz, DMSO-*d*_6_) *δ* 153.6, *δ* 146.9 (d, *J* = 11.7 Hz), 146.3 (d, *J* = 6.5 Hz), 145.9 (d, *J* = 8.3 Hz), 134.2, 133.7, 132.2, 131.9, 131.7 (d, *J* = 4.5 Hz), 131.6, 131.5, 130.2, 129.2 (d, *J* = 9.1 Hz), 128.7, 128.3 (d, *J* = 13.0 Hz), 127.8, 127.5 (d, *J* = 7.4 Hz), 126.7, 126.6, 126.6, 126.5, 126.2, 124.5 (d, *J* = 7.6 Hz), 124.4, 123.0, 121. 0, 120.8, 120.2, 119.7, 118.7, 113.2; ^31^P NMR (161 MHz, DMSO-*d*_6_) *δ* −2.59. HRMS (ESI) calcd for [C_40_H_35_NaO_5_P, M+Na]^+^: 639.1332, found: 639.1328.

#### 3.1.4. Synthesis of (S, R)-**P7**


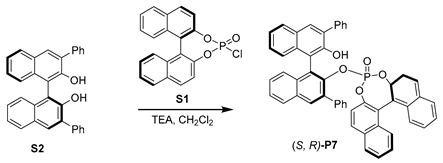


Under a nitrogen atmosphere, to a solution of **S1** (36.6 mg, 0.1 mmol) and **S2** (43.8 mg, 0.1 mmol) in anhydrous CH_2_Cl_2_ (10 mL), Et_3_N (30 mg, 0.3 mmol) was added at 0 °C. The reaction was stirred at room temperature overnight. The solvent was removed under vacuum and the residue was purified using flash column chromatography (CH_2_Cl_2_/PE) to obtain **P7** as a white solid (616 mg, 80% yield). ^1^H NMR (400 MHz, CDCl_3_) *δ* 8.10 (t, *J* = 9.3 Hz, 2H), 7.90 (d, *J* = 8.2 Hz, 1H), 7.87–7.79 (m, 3H), 7.71 (d, *J* = 7.9 Hz, 1H), 7.52 (m, 3H), 7.44–7.37 (m, 2H), 7.24 (m, 5H), 7.15–7.09 (m, 1H), 6.98 (m, 3H), 6.88 (d, *J* = 5.8 Hz, 1H), 6.80 (m, 5H), 6.60–6.53 (m, 1H), 6.28 (m, 5H). ^13^C NMR (100 MHz, CDCl_3_) *δ* 151.4, 146.3, *δ* 146.2 (d, *J* = 8.2 Hz), 144.4 (d, *J* = 11.7 Hz), 144.3, 143.5 (d, *J* = 8.9 Hz), 143.4, 138.9, 138.6, 138.5, 138.4, 133.3, 133.0, 132.9 (d, *J* = 2.0 Hz), 132.8, 131.1, 130.3, 129.4, 128.2, 127.9, 127.8, 127.2, 127.0, 126.8, 126.8, 126.7, 126.6, 126.5, 126.4, 125.7, 125.6, 125.6 (d, *J* = 2.2 Hz), 125.5, 125.3, 125.1, 124.4 (d, *J* = 2.6 Hz), 124.3, 123.7, 123.6, 1123.5 (d, *J* = 3.5 Hz), 122.9, 122.8, 122.7, 121.6 (d, *J* = 2.0 Hz), 121.0, 120.7 (d, *J* = 2.1 Hz). 120.4, 119.3, 118.5, 114.7. ^31^P NMR (161 MHz, CDCl_3_) *δ* −0.28.

#### 3.1.5. Synthesis of (S, R)-**P8**


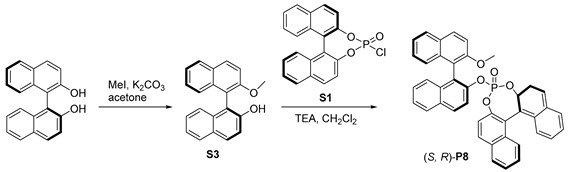


Preparation of **S3**:

The (*S*)-(+)-1,1’-bi-2-naphthol (1.43 g, 5 mmol) and K_2_CO_3_ (828 mg, 6 mmol) was dissolved in dry acetone (40 mL) and MeI (775 mg, 5.5 mmol) was added dropwise under a nitrogen atmosphere at 30 °C. The slurry was stirred for 18 h under reflux. The solid was removed via filtration. The filtrate was concentrated and purified using flash column chromatography (EtOAc/PE) to obtain product **S3** as a white solid (1.47 g, 98% yield). 1H NMR (400 MHz, CDCl_3_) *δ* 8.04 (d, *J* = 9.0 Hz, 1H), 7.92–7.90 (m, 2H), 7.86 (d, *J* = 8.1 Hz, 1H), 7.49 (d, J = 9.1 Hz, 1H), 7.39–7.28 (m, 4H), 7.22 (ddd, *J =* 8.2, 6.8, 1.3 Hz, 1H), 7.17 (d, *J* = 8.1 Hz, 1H), 7.05 (d, *J* = 8.4 Hz, 1H), 3.81 (s, 3H). ^13^C NMR (100 MHz, CDCl_3_) *δ* 156.1, 151.3, 134.1, 133.9, 131.2, 129.9, 129.5, 129.3, 128.3, 128.3, 127.5, 126.5, 125.0, 124.9, 124.3, 123.4, 117.6, 115.4, 115.1, 113.9, 56.8.

Preparation of (*S*, *R*)-**P8.**

Under a nitrogen atmosphere, to a solution of **S1** (366 mg, 1 mmol) and **S3** (300 mg, 1 mmol) in anhydrous CH_2_Cl_2_ (10 mL), Et_3_N (0.45 mL, 3 mmol) was added at 0 °C. The reaction was stirred at room temperature overnight. The solvent was removed under vacuum and the residue was purified using flash column chromatography (CH_2_Cl_2_/PE) obtain product (*S*, *R*)-**P8** as a white solid (505 mg, 80% yield). ^1^H NMR (400 MHz, CDCl_3_) *δ* 7.80 (d, *J* = 9.0 Hz, 1H), 7.68 m, 3H), 7.58 (dd, *J* = 12.4, 8.2 Hz, 2H), 7.46 (m, 2H), 7.26 (d, *J* = 8.2 Hz, 2H), 7.20–7.15 (m, 2H), 7.12 (d, *J* = 7.6 Hz, 1H), 7.06–6.99 (m, 4H), 6.93 (dt, *J* = 11.7, 7.5 Hz, 4H), 6.86–6.78 (m, 2H), 6.09 (d, *J* = 8.8 Hz, 1H), 3.54 (s, 3H).^13^C NMR (100 MHz, CDCl_3_) *δ* 171.0, 155.0, 147.0, 146.8, 146.0, 133.8, 133.7, 132.0, 131.7, 131.6, 131.5, 131.4, 131.2, 130.6, *δ* 129.9 (d, *J* = 5.8 Hz), 129.8, 129.8, 128.5, 128.4, 128.3 (d, *J* = 11.1 Hz), 128.2, 128.1, 127.6, 127.1, 126.8, 126.6, 126.4, 126.3 (d, *J* = 9.7 Hz),, 126.1, 125.6 (d, *J* = 10.3 Hz), 125.5, 123.3, 121.1, 120.5, 120.4, 119.9, 119.6, 116.8, 113.3, 56.4. ^31^P NMR (161 MHz, CDCl_3_) *δ* −2.76.

#### 3.1.6. Synthesis of (R, R)-**P9** [57]


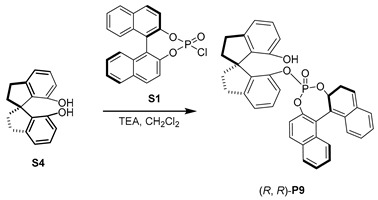


Under a nitrogen atmosphere, to a solution of **S1** (3 g, 8 mmol) and (*R*)-2,2’,3,3’-tetrahydro-1,1’-spirobi [1H-indene]-7,7’-diol (**S4**, 2 g, 8 mmol) in anhydrous CH_2_Cl_2_ (50 mL), Et_3_N (1.2 mL, 8 mmol) was added at 0 °C. The reaction was stirred at room temperature overnight. The solvent was removed under vacuum and the residue was purified using flash column chromatography (CH_2_Cl_2_/PE) to obtain product **P9** as a white solid (3.3 g, 72% yield). ^1^H NMR (400 MHz, CDCl_3_) *δ* 8.06 (d, *J* = 9.0 Hz, 1H), 7.96 (t, *J* = 8.8 Hz, 2H), 7.91 (d, *J* = 8.9 Hz, 1H), 7.65 (d, *J* = 8.9 Hz, 1H), 7.50 (t, *J* = 7.4 Hz, 2H), 7.32 (td, *J* = 14.2, 6.1 Hz, 6H), 7.16 (d, *J* = 7.3 Hz, 1H), 6.93 (s, 2H), 6.66 (d, *J* = 8.8 Hz, 1H), 6.52 (d, *J* = 5.7 Hz, 1H), 3.14–3.01 (m, 3H), 2.93 (dd, *J* = 15.8, 8.6 Hz, 1H), 2.30 (q, *J* = 7.4, 5.0 Hz, 2H), 2.20 (dd, *J* = 12.5, 7.3 Hz, 1H), 2.06 (d, *J* = 12.1 Hz, 1H); ^13^C NMR (100 MHz, CDCl_3_) *δ* 153.2, 147.6 (d, *J* = 1.7 Hz), 147.5 (d, *J* = 2.6 Hz), 147.3, *δ* 146.6 (d, *J* = 8.9 Hz), 145.3, 138.1 (d, *J* = 8.5 Hz), 135.1, 132.6, 132.4, 132.3, 132.1, 131.9, 131.3, 129.0, 128.9, 128.8, 127.5, 127.4, 127.21, 127.0, 126.3, 126.2, 122.5, 117.4, 121.9 (d, *J* = 2.2 Hz), 121.1 (d, *J* = 2.1 Hz), 120.9 (d, *J* = 2.8 Hz), 120.7 (d, *J* = 3.2 Hz), 117.2, 116.2, 59.2, 38.1, 37.8, 31.6 (d, *J* = 6.2 Hz); ^31^P NMR (162 MHz, CDCl_3_) *δ* −2.02. HRMS (ESI) calcd for [C_37_H_27_NaO_5_P, M+Na]^+^: 605.1488, found: 605.1474.

#### 3.1.7. Synthesis of (*S*, *R*)-**P10**

The (*S*, *R*)-**P10** was prepared according to the (*R*, *R*)-**P9** procedure.

^1^H NMR (400 MHz, CDCl_3_) *δ* 7.97 (d, *J* = 8.9 Hz, 1H), 7.93–7.87 (m, 2H), 7.83 (s, 1H), 7.45 (t, *J* = 8.1 Hz, 3H), 7.29 (m, 6H), 7.15 (d, *J* = 7.3 Hz, 1H), 7.00 (t, *J* = 7.7 Hz, 1H), 6.85 (d, *J* = 8.9 Hz, 1H), 6.59 (dd, *J* = 15.6, 7.7 Hz, 2H), 3.03 (t, *J* = 7.3 Hz, 2H), 2.89 (dd, *J* = 16.6, 7.6 Hz, 1H), 2.80 (dd, *J* = 15.7, 8.6 Hz, 1H), 2.30 (d, *J* = 9.9 Hz, 1H), 2.21 (dt, *J* = 22.2, 7.6 Hz, 3H); ^13^C NMR (100MHz, CDCl_3_) *δ* 152.5, 147.4 (d, *J* = 6.7 Hz), 147.4, 147.3 (d, *J* = 11.5 Hz), 146.1 (d, *J* = 8.5 Hz), 145.1, 137.8 (d, *J* = 7.3 Hz), 134.6, 132.2, 131.9, 131.7, 131.5, 131.1, 129.2, 128.5, 128.4, 128.2, 127.3, 127.0, 126.7, 126.6, 125.8 (d, *J* = 6.1 Hz), 122.6, 121.4 (d, *J* = 2.4 Hz), 120.9 (d, *J* = 2.1 Hz), 120.7 (d, *J* = 3.1 Hz), 120.2 (d, *J* = 3.3 Hz), 117.9, 117.4, 115.3, 58.8, 38.0, 37.8, 31.3, 31.1; ^31^P NMR (161 MHz, CDCl_3_) *δ* −1.91. HRMS (ESI) calcd for [C_37_H_27_NaO_5_P, M+Na]^+^: 605.1488, found: 605.1480.

#### 3.1.8. Synthesis of (S, S, S, R)-**P11**


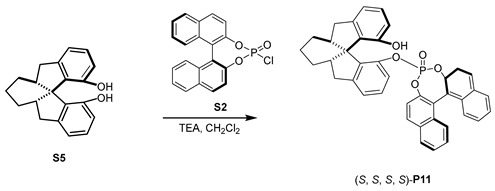


Under a nitrogen atmosphere, to a solution of **S2** (366 mg, 1 mmol) and cyclohexyl-fused chiral spirobiindanediol [58] **S5** (292 mg, 1 mmol) in anhydrous CH_2_Cl_2_ (8 mL), Et_3_N (0.15 mL, 1 mmol) was added at 0 °C. The reaction was stirred at room temperature overnight. The solvent was removed under vacuum and the residue was purified using flash column chromatography (CH_2_Cl_2_/PE) to obtain product **P11** as a white solid (466 mg, 75% yield). ^1^H NMR (400 MHz, CDCl_3_) *δ* 8.01 (d, *J* = 8.9 Hz, 1H), 7.94 (t, *J* = 7.9 Hz, 2H), 7.85 (d, *J* = 8.9 Hz, 1H), 7.57 (d, *J* = 8.9 Hz, 1H), 7.53–7.43 (m, 3H), 7.30 (m, 5H), 7.16 (d, *J* = 7.4 Hz, 1H), 7.06 (d, *J* = 8.9 Hz, 1H), 6.87 (t, *J* = 7.6 Hz, 1H), 6.76 (d, *J* = 7.3 Hz, 1H), 6.35 (d, *J* = 7.9 Hz, 1H), 3.15 (dd, *J* = 15.9, 7.5 Hz, 1H), 2.73 (dd, *J* = 15.5, 6.1 Hz, 2H), 2.65 (s, 1H), 2.57 (s, 1H), 2.29 (dd, *J* = 15.1, 7.6 Hz, 1H), 1.47 (m, 4H), 1.28–1.19 (m, 2H); ^13^C NMR (100 MHz, CDCl_3_) *δ* 152.7, *δ* 148.3 (d, *J* = 6.6 Hz), 147.7, 147.1 (d, *J* = 11.6 Hz), 146.2 (d, *J* = 8.7 Hz), 144.9, 135.7 (d, *J* = 8.1 Hz), 133.3, 132.2 (d, *J* = 7.7 Hz), 131.8, 131.6, 131.5, 131.1, 129.2, 128.5 (d, *J* = 8.9 Hz), 128.0, 127.1, 126.9, 126.8 (d, *J* = 6.9 Hz), 125.9, 123.0, 121.4 (d, *J* = 2.3 Hz), 120.9 (d, *J* = 2.3 Hz), 120.5 (d, *J* = 2.9 Hz), 120.0 (d, *J* = 3.4 Hz), 118.3, 117.2, 115.3, 61.1, 44.4, 43.0, 37.8, 36.0, 25.8, 23.5, 17.2; ^31^P NMR (161 MHz, CDCl_3_) *δ* −2.93. HRMS (ESI) calcd for [C_40_H_31_NaO_5_P, M+Na]^+^: 645.1801, found: 645.1791.

### 3.2. Deuteration of Styrene


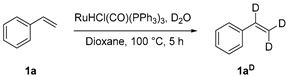


A mixture of RuHCl(CO)(PPh_3_)_3_ (38.1 mg, 0.04 mmol, 2.0 mol%), styrene (230 μL, 2.0 mmol) and D_2_O(1 mL) in dioxane (4 mL) in a stoppered Schlenck tube was stirred and heated at 100 °C for 5 h. The reaction mixture was cooled to room temperature and then extracted with diethyl ether. The combined organic extract was washed with water and a brine solution and dried over MgSO_4_. The crude product was purified using column chromatography on silica gel using n-hexane as the eluent to obtain the desired product **1a^D^** as a colorless oil (128 mg, 60% yield). Theoretical percentage of deuteration at the vinyl position = 97%. ^1^H NMR (400 MHz, CDCl_3_) *δ* 7.43–7.41 (m, 2H), 7.36–7.31(m, 2H), 7.28–7.24 (m, 1H), 6.71 (brs, 0.03H), 5.74–5.73 (m, 0.03H), 5.23(m, 0.03H).

### 3.3. General Procedure for Hydroformylation

The hydroformylation reactions were conducted in a batch reactor (Shanghai Yanzheng). In a typical run, 0.001 mmol of Chloro(1,5-cyclooctadiene)rhodium(I) dimer, 0.006 mmol of ligand (Rh/P = 1:3) was dissolved in 25 mL toluene, and then the solution of substrate (3.0 mmol) was added. Subsequently, the reactor was charged with 4.0 MPa syngas (CO/H_2_ = 1:1) for 12–48 h at 30 °C. The products were analyzed with GC and GC-MS. The yield and the regioselectivity of aldehydes were identified using GC. The mixture was concentrated under reduced pressure. Then, the crude product was purified using flash chromatography on silica gel to obtain the desired aldehyde.

**2-(2-Fluorophenyl)propanal (2b)** Colorless oil, 94% yield. ^1^H NMR (400 MHz, CDCl_3_) *δ* 9.67 (d, *J* = 0.7 Hz, 1H), 7.22 (m, 1H), 7.11–7.01 (m, 3H), 3.84 (q, *J* = 7.1 Hz, 1H), 1.38 (d, *J* = 7.2 Hz, 3H).
**2-(3-Fluorophenyl)propanal (2c)** Colorless oil, 94% yield. ^1^H NMR (400 MHz, CDCl_3_) *δ* 9.56 (d, *J* = 1.4 Hz, 1H), 7.27–7.21 (m, 1H), 6.92–6.87 (m, 2H), 6.85–6.81 (m, 1H), 3.54 (q, *J* = 6.4 Hz, 1H), 1.34 (d, *J* = 7.1 Hz, 3H).
**2-(4-fluorophenyl)propanal (2d)** Colorless oil, 93% yield. ^1^H NMR (400 MHz, CDCl_3_) *δ* 9.67 (d, *J* = 1.3 Hz, 1H), 7.38–7.34 (m, 2H), 7.18–7.14 (m, 2H), 3.64 (q, *J* = 6.7 Hz, 1H), 1.45 (d, *J* = 7.1 Hz, 3H).
**2-(2-chlorophenyl)propanal (2e)** Colorless oil, 95% yield. ^1^H NMR (400 MHz, CDCl_3_) *δ* 9.72 (s, 1H), 7.44 (dd, *J* = 7.5, 1.8 Hz, 1H), 7.31–7.22 (m, 2H), 7.14 (dd, *J* = 7.3, 2.1 Hz, 1H), 4.14 (q, *J* = 7.1 Hz, 1H), 1.44 (d, *J* = 7.1 Hz, 3H).
**2-(3-chlorophenyl)propanal (2f)** Colorless oil, 94% yield. ^1^H NMR (400 MHz, CDCl_3_) *δ* 9.68 (d, *J* = 1.3 Hz, 1H), 7.35–7.28 (m, 2H), 7.23 (d, *J* = 1.9 Hz, 1H), 7.11 (m, 1H), 3.64 (q, *J* = 6.7 Hz, 1H), 1.46 (d, *J* = 7.1 Hz, 3H).
**2-(4-Chlorophenyl)propanal (2g)** Colorless oil, 93% yield. ^1^H NMR (400 MHz, CDCl_3_) *δ* 9.64 (d, *J* = 1.3 Hz, 1H), 7.36–7.31 (m, 2H), 7.16–7.12 (m, 2H), 3.62 (q, *J* = 7.1 Hz, 1H), 1.42 (d, *J* = 7.1 Hz, 3H).
**2-(2-bromophenyl)propanal (2h)** Colorless oil, 91% yield. ^1^H NMR (400 MHz, CDCl_3_) *δ* 9.65 (s, 1H), 7.55 (dd, *J* = 8.0, 1.3 Hz, 1H), 7.27–7.22 (m, 1H), 7.09 (m, 1H), 7.03 (dd, *J* = 7.7, 1.7 Hz, 1H), 4.08 (q, *J* = 7.1 Hz, 1H), 1.34 (d, *J* = 7.1 Hz, 3H).
**2-(3-bromophenyl)propanal (2i)** Colorless oil, 94% yield. ^1^H NMR (400 MHz, CDCl_3_) *δ* 9.50 (d, *J* = 1.3 Hz, 1H), 7.29–7.23 (m, 2H), 7.10 (t, *J* = 7.8 Hz, 1H), 7.00 (m, 1H), 3.47 (q, *J* = 7.1, 6.4 Hz, 1H), 1.28 (d, *J* = 7.1 Hz, 3H).
**2-(4-bromophenyl)propanal (2j)** Colorless oil, 93% yield. ^1^H NMR (400 MHz, CDCl_3_) *δ* 9.66 (d, *J* = 1.3 Hz, 1H), 7.53–7.47 (m, 2H), 7.12–7.05 (m, 2H), 3.62 (q, *J* = 7.1, 6.7 Hz, 1H), 1.44 (d, *J* = 7.1 Hz, 3H).
**2-(4-nitrophenyl)propanal (2k)** Yellow solid, m.p. 39.5–40.1 °C, 94% yield. ^1^H NMR (400 MHz, CDCl_3_) *δ* 9.65 (d, *J* = 1.1 Hz, 1H), 8.17 (d, *J* = 8.7 Hz, 2H), 7.33 (d, *J* = 8.7 Hz, 2H), 3.73 (q, *J* = 7.1 Hz, 1H), 1.45 (d, *J* = 7.2 Hz, 3H).
**2-(*o*-Tolyl)propanal (2l)** Colorless oil, 92% yield. ^1^H NMR (400 MHz, CDCl_3_) *δ* 9.52 (s, 1H), 7.12–7.06 (m, 3H), 6.92 (d, *J* = 6.4 Hz, 1H), 3.72 (q, *J* = 7.0 Hz, 1H), 2.24 (s, 3H), 1.29 (d, *J* = 8.3 Hz, 3H).
**2-(*m*-Tolyl)propanal (2m)** Colorless oil, 92% yield. ^1^H NMR (400 MHz, CDCl_3_) *δ* 9.59 (d, *J* = 1.0 Hz, 1H), 7.18 (t, *J* = 7.9 Hz, 1H), 7.03 (d, *J* = 7.5 Hz, 1H), 6.92 (d, *J* = 6.6 Hz, 2H), 3.51 (q, *J* = 7.0 Hz, 1H), 2.27 (s, 3H), 1.34 (d, *J* = 7.1 Hz, 3H).
**2-(*p*-Tolyl)propanal (2n)** Colorless oil, 92% yield. ^1^H NMR (400 MHz, CDCl_3_) *δ* 9.66 (d, *J* = 1.3 Hz, 1H), 7.20 (d, *J* = 7.9 Hz, 2H), 7.10 (d, *J* = 8.0 Hz, 2H), 3.60 (q, *J* = 6.9 Hz, 1H), 2.35 (s, 3H), 1.42 (d, *J* = 7.1 Hz, 3H).
**2-(4-*iso*-Butylphenyl)propanal (2o)** Colorless oil, 92% yield. ^1^H NMR (400 MHz, CDCl_3_) *δ* 9.55 (d, *J* = 1.4 Hz, 1H), 7.06–6.98 (m, 4H), 3.49 (q, *J* = 6.1 Hz, 1H), 2.36 (d, *J* = 7.2 Hz, 2H), 1.75 (m, 1H), 1.31 (d, *J* = 7.1 Hz, 3H), 0.80 (d, *J* = 6.6 Hz, 6H).
**2-(4-(*tert*-Butyl)phenyl)propanal (2p)** Colorless oil, 88% yield. ^1^H NMR (400 MHz, CDCl_3_) *δ* 9.71 (d, *J* = 1.3 Hz, 1H), 7.44 (d, *J* = 8.3 Hz, 2H), 7.19 (d, *J* = 8.2 Hz, 2H), 3.65 (q, *J* = 7.0 Hz, 1H), 1.47 (d, *J* = 7.1 Hz, 3H), 1.36 (s, 9H).
**2-(4-Hydroxyphenyl)propanal (2q)** Colorless oil, 92% yield. ^1^H NMR (400 MHz, CDCl_3_) *δ* 9.53 (d, *J* = 1.0 Hz, 1H), 6.96 (d, *J* = 8.4 Hz, 2H), 6.77 (d, *J* = 8.4 Hz, 2H), 6.57 (s, 1H), 3.50 (q, *J* = 6.8 Hz, 1H), 1.31 (d, *J* = 7.1 Hz, 3H).
**2-(4-Methoxyphenyl)propanal (2r)** Colorless oil, 91% yield. ^1^H NMR (400 MHz, CDCl_3_) *δ* 9.53 (d, *J* = 1.4 Hz, 1H), 7.05–7.00 (m, 2H), 6.84–6.79 (m, 2H), 3.69 (s, 3H), 3.48 (q, *J* = 7.1 Hz, 1H).
**2-(2,5-dimethylphenyl)propanal (2s)** Colorless oil, 91% yield. ^1^H NMR (400 MHz, CDCl_3_) *δ* 9.71 (d, *J* = 1.1 Hz, 1H), 7.19 (d, *J* = 7.7 Hz, 1H), 7.08 (d, *J* = 9.1 Hz, 1H), 6.92 (s, 1H), 3.91–3.84 (m, 1H), 2.39 (s, 3H), 2.38 (s, 3H), 1.46 (d, *J* = 7.0 Hz, 3H).
**2-(3,4-dimethoxyphenyl)propanal (2t)** Colorless oil, 89% yield. ^1^H NMR (400 MHz, CDCl_3_) *δ* 9.54 (d, *J* = 1.4 Hz, 1H), 6.78 (d, *J* = 8.2 Hz, 1H), 6.66 (dd, *J* = 8.2, 2.0 Hz, 1H), 6.60 (d, *J* = 2.0 Hz, 1H), 3.77 (s, 3H), 3.77 (s, 3H), 3.51–3.44 (m, 1H), 1.32 (d, *J* = 7.1 Hz, 3H).
**2-(naphthalen-2-yl)propanal (2u)** White solid, m.p. 87.0–88.2 °C, 92% yield. ^1^H NMR (400 MHz, CDCl_3_) *δ* 9.80 (d, *J* = 1.4 Hz, 1H), 7.92–7.86 (m, 3H), 7.72 (s, 1H), 7.60–7.51 (m, 2H), 7.36 (dd, *J* = 8.4, 1.8 Hz, 1H), 3.82 (q, *J* = 6.6 Hz, 1H), 1.59 (d, *J* = 7.1 Hz, 3H).

## 4. Conclusions

In this study, we successfully developed a novel hybrid phosphate as a promoter for rhodium-catalyzed hydroformylation of styrenes, facilitating the synthesis of a variety of branched aldehydes with excellent yields and impressive regioselectivities. Moreover, this hybrid phosphate exhibited exceptional stability under standard conditions. Furthermore, mechanistic studies highlighted the weak coordination of rhodium catalyst, with phosphate likely accelerating the hydrogenolysis step in the catalytic cycle. The potential of application in hydroformylation reactions along with a comprehensive analysis of the reaction mechanism are currently ongoing in our lab.

## Figures and Tables

**Table 1:** Screening P(V) reagents for Rh-catalyzed hydroformylation of styrene ^a^.

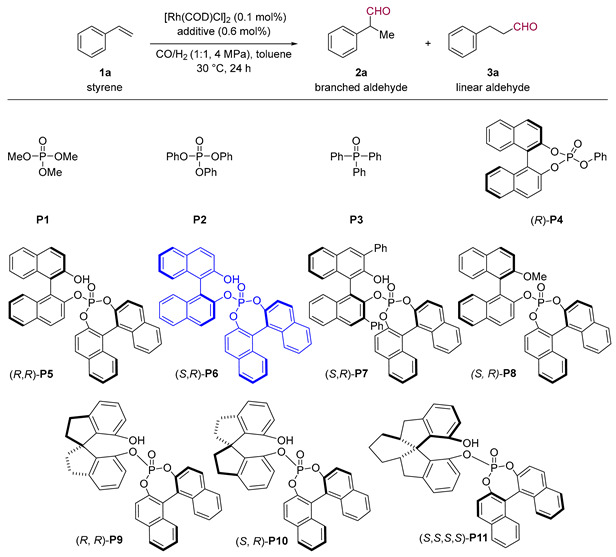			
**Entry**	**P(V) Reagents**	**Yield of 2a (%)** ^**b**^	***b*/*l* (2a/3a)** ^**b**^
1	**P1**	16	8.0:1
2	**P2**	40	6.6:1
3	**P3**	5	/
4	**P4**	10	/
5	**P5**	28	23.4:1
6 ^c^	**P6**	96	25.4:1
7 ^c^	**P7**	75	15.0:1
8 ^d^	**P8**	49	5.2:1
9	**P9**	27	22.8:1
10	**P10**	33	22.2:1
11	**P11**	6	/

^a^ Reaction conditions: styrene **1a** (3.0 mmol), [Rh(COD)Cl]_2_ (0.1 mol %), P(V) reagents (0.6 mol %), toluene (25 mL), syngas (CO/H_2_ = 1), 4.0 MPa, 30 °C, 24 h. ^b^ Determined using GC. ^c^ 0% ee of branched aldehyde. ^d^ 48 h.

**Table 2:** Optimization of Rh-catalyzed regioselective hydroformylation of styrene with **P6** ^a^.

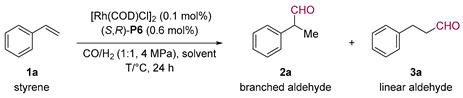						
**Entry**	**Rh/P6**	**T (°C)**	**P (MPa)**	**Solvent**	**Yield of 2a (%)** ^**b**^	***b*/*l* (2a/3a)** ^**b**^
1	1/3	30	4.0	toluene	96	25.4:1
2	1/3	40	4.0	toluene	92	12.1:1
3	1/3	50	4.0	toluene	87	6.7:1
4	1/2	30	40	toluene	90	19.5:1
5	1/6	30	4.0	toluene	31	20.1:1
6	1/8	30	4.0	toluene	22	20.1:1
7	1/3	30	3.0	toluene	12	18.4:1
8	1/3	30	2.0	toluene	6	/
9	1/3	30	4.0	THF	95	18.0:1
10	1/3	30	4.0	DCM	4	/
11	1/3	30	4.0	Et_2_O	71	18.9:1
12 ^c^	1/3	30	4.0	toluene	95	20.3:1
13 ^d^	1/3	30	4.0	toluene	17	22.3:1

^a^ Reaction conditions: **1a** (3.0 mmol), [Rh(COD)Cl]_2_ (0.1 mol %), **P6** (0.3–0.8%), organic solvent (25 mL), syngas (CO/H_2_ = 1:1). ^b^ Determined using GC. ^c^ Rh(acac)(CO)_2_ (0.1 mol%), ^d^ RhCl_3_ (0.1 mol%).

## Data Availability

The original contributions presented in the study are included in the Supplementary Material, further inquiries can be directed to the corresponding authors.

## Supplementary Materials

The following supporting information can be downloaded at: https://www.mdpi.com/article/10.3390/molecules29092039/s1, Table S1. Effect of the concentration of TPP and P6. Table S2. Competition isotope effect of deuterated styrene. Figure S1. GC spectra of reaction crude sample. Figure S2. HRMS-ESI spectra of Rh(COD)P6. Figure S3. HRMS-ESI spectra of Rh(CO)_2_P6. IR spectra: Figures S4–S55. Scheme S1. Competition Isotope Effect of deuterated styrene. Scheme S2. Competition isotope effect under H_2_/D_2_ atmosphere. References [35,37,48,55,56,57,58,59,60,61,62,63,64,65,66,67,68,69] are cited in the supplementary materials.
