# Supplementary material for: P(V)-Promoted Rh-Catalyzed Highly Regioselective Hydroformylation of Styrenes under Mild Conditions
Source: Molecules. 2024 Apr 28;29(9):2039. doi: 10.3390/molecules29092039 (PMC11085418; doi:10.3390/molecules29092039)
Supplement: Supplementary file 1 [file molecules-29-02039-s001.zip › molecules-2983766-supplementary.pdf]

P(V)-Promoted Rh-Catalyzed Highly Regioselective Hydroformylation of Styrenes under Mild Conditions

Tong Ru [1,2,3], Yajiao Zhang [2,3,4], Qiuxiang Wei [2,3,4], Sheng Zuo [2,3], Zhenhua Jia [2,3,*] and Fen-Er Chen [1,2,3,4,*]

[1] Department of Medicinal Chemistry, School of Pharmacy, Fudan University, Shanghai 201203, China; 19111030025@fudan.edu.cn
[2] Engineering Center of Catalysis and Synthesis for Chiral Molecules, Department of Chemistry, Fudan University, Shanghai 200433, China; 200410027@fzu.edu.cn (Y.Z.); qiuxiangwei21@163.com (Q.W.)
[3] Shanghai Engineering Center of Industrial Asymmetric Catalysis for Chiral Drugs, Shanghai 200433, China
[4] College of Chemical Engineering, Fuzhou University, Fuzhou 350102, China
* Correspondence: zhenhua_jia@fudan.edu.cn (Z.J.); rfchen@fudan.edu.cn (F.-E.C.)

Supporting Information

CONTENT

1. **Supporting tables and figures** S2
2. **General information** S6
3. **Synthesis of the phosphate** S6
   3.1 Synthesis of (*R*)-**P4** S7
   3.2 Synthesis of (*R*, *R*)-**P5** S7
   3.3 Synthesis of (*S*, *R*)-**P6** S8
   3.4 Synthesis of (*S*, *R*)-**P7** S8
   3.5 Synthesis of (*S*, *R*)-**P8** S9
   3.6 Synthesis of (*R*, *R*)-**P9** S9
   3.7 Synthesis of (*S*, *R*)-**P10** S11
   3.8 Synthesis of (*S*, *S*, *S*, *S*)-**P11** S11
4. **Deuteration of styrene** S12
5. **General procedure for hydroformylation** S12
6. **Characterization data of *rac*-*α*-aryl propionaldehydes** S13
7. **Isotope effect measurement** S18
8. **References** S19
9. **IR spectra** S20

10. NMR spectra S22

1. Supporting table and figures

Table S1 Effect of the concentration of TPP and **P6**.

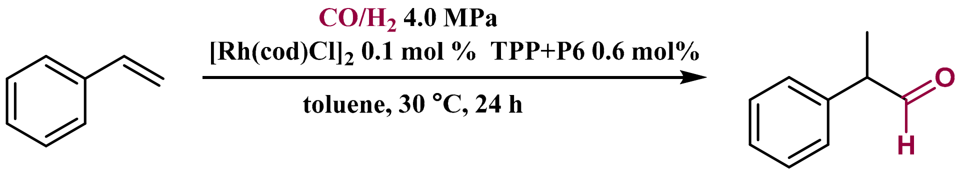

1a **2a**

Entry	TPP (mol%)	**P6** (mol%)	Yield [a]
1	0.6	0	4.9%
2	0.5	0.1	6%
3	0.4	0.2	7%
4	0.3	0.3	10%
5	0.2	0.4	21%
6	0.1	0.5	40%
7[b]	0	0.6	96%

[a] Yields were determined by GC-MS; [b] Standard condition.

Area Percent Report

```
Data Path : D:\Data\2024\
Data File : 20240121-RT-S.D
Acq On    : 21 Jan 2024  23:10
Operator  :
Sample    : 20240121-RT-S
Misc      :
ALS Vial  : 10   Sample Multiplier: 1

Integration Parameters: autoint1.e
Integrator: ChemStation

Method    : D:\Data\METHODS\GEN-01-45C-01.M
Title     :

Signal     : TIC: 20240121-RT-S.D\data.ms
```

peak #	R.T. min	first scan	max scan	last scan	PK TY	peak height	corr. area	corr. % max.	% of total
1	5.161	264	272	387	M	11327650	365768597	100.00%	96.230%
2	6.414	394	405	419	M	106945	14331416	3.92%	3.770%

Sum of corrected areas: 380100013

GEN-01-45C-01.M Tue Jan 23 15:48:40 2024

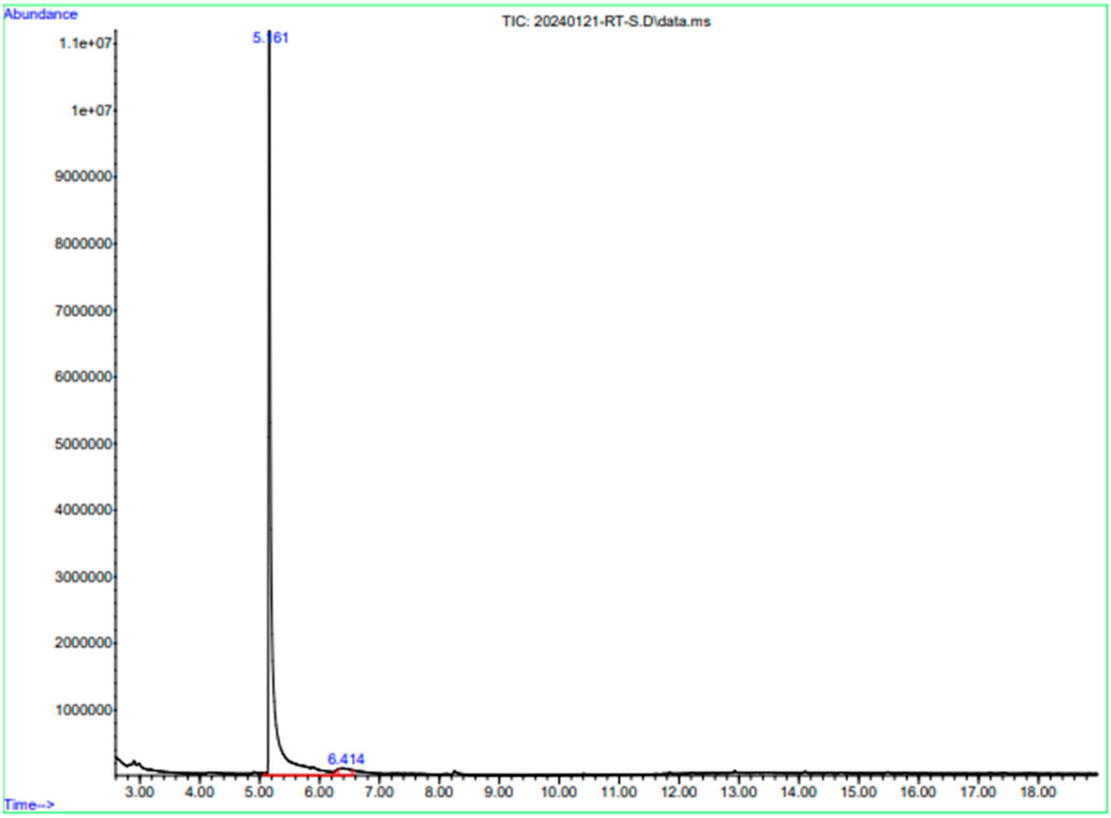

Figure S1 GC diagram of crude reaction mixture

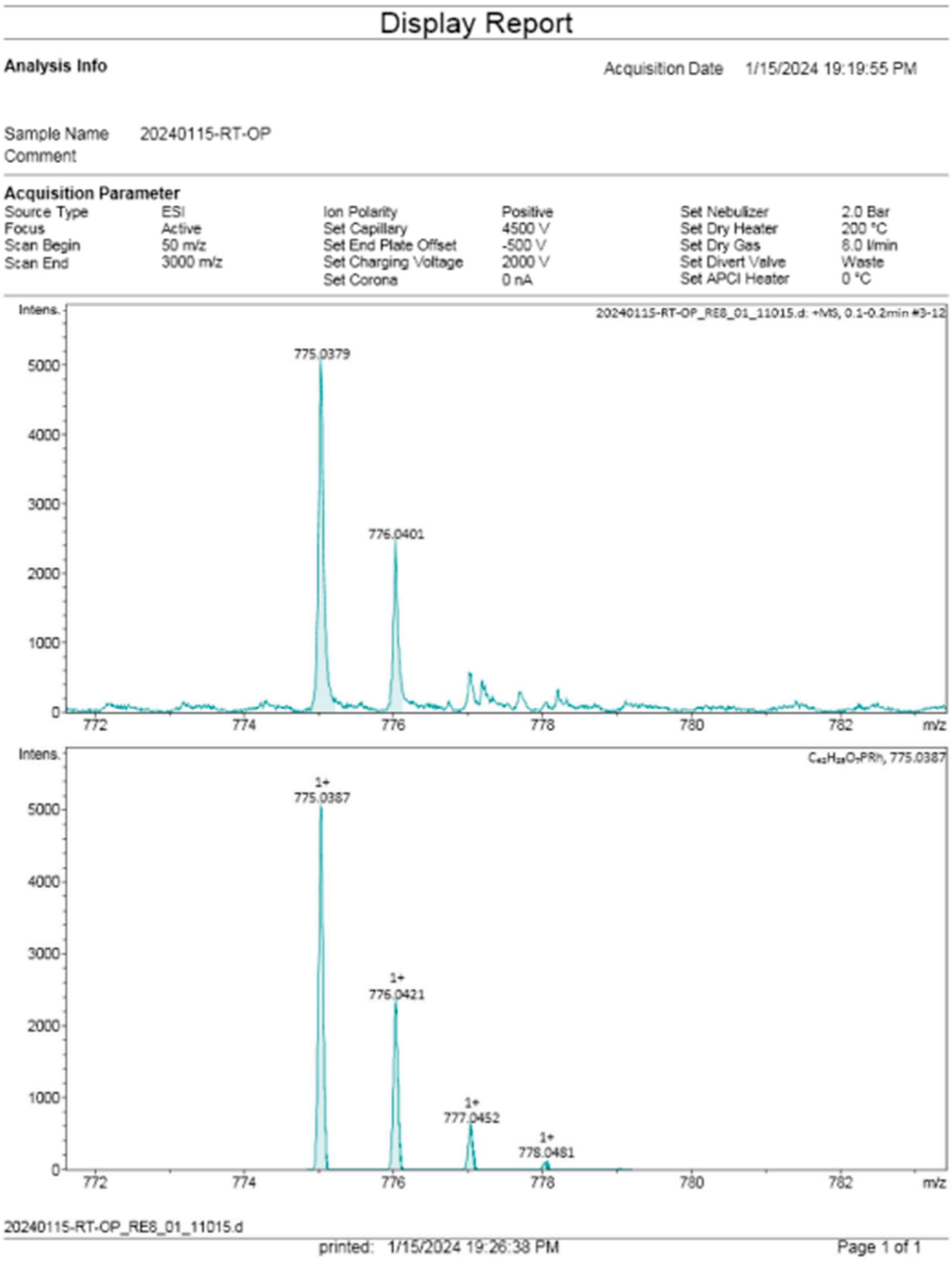

Figure S2 HRMS-ESI of Rh(COD)P6.

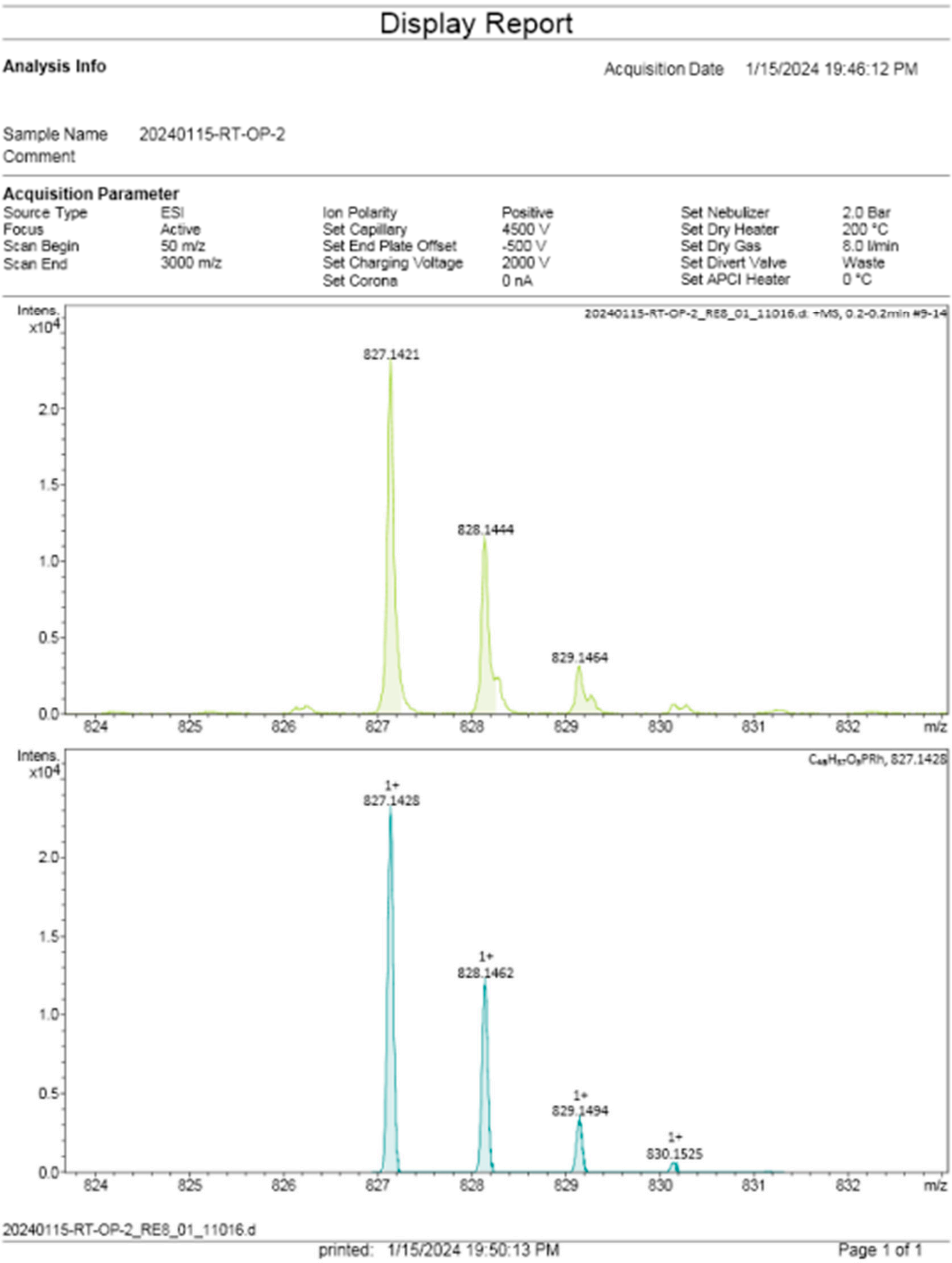

Figure S3 HRMS-ESI of $Rh(CO)_2P6$.

2. General information

All commercial regents were used without further purification. When necessary, solvents were dried with standard procedures. NMR spectra were recorded on Bruker ADVANCE III (400 MHz) spectrometer. $CDCl_3$ or DMSO-d_6 was the solvent used for the NMR analysis, with tetramethylsilane as the internal standard. Data are reported as follows: chemical shift [multiplicity (br = broad, s = singlet, d = doublet, t = triplet, m = multiplet), coupling constant(s) in Hertz, integration]. GC-MS analysis was carried out on Angilent 7820A GC system and Angilent 5977B MSD. HRMS were recorded on a Bruker micrOTOF spectrometer (ESI). IR spectra were carried out on ThermoFisher NICOLET iS10 IR spectrometer.

3. Synthesis of the phosphates

3.1 Synthesis of (*R*)-**P4**[1,2,3,4]

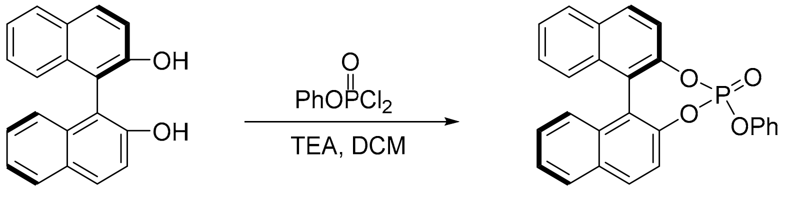

(*R*)-P4

The (*R*)-(+)-1,1'-bi-2-naphthol (858 mg, 3 mmol) and Et_3N (1.2 ml, 9 mmol) was dissolved in dry CH_2Cl_2 (10 ml), and then phenyl dichlorophosphate (756 mg, 3.6 mmol) was added dropwisely under argon at 0 °C. The reaction was allowed to warm to room temperature and stirred overnight. After that, the solid was removed by filtration. The filtrate was concentrated and purified by flash column chromatography (CH_2Cl_2 /PE) to give product **P4** as a white solid (1.14 g, 90 % yield). ^{1}H NMR (400 MHz, $CDCl_3$) δ 7.96 (d, J = 8.9 Hz, 1H), 7.90 (d, J = 8.9 Hz, 1H), 7.85 (d, J = 8.2 Hz, 2H), 7.55 (d, J = 8.9 Hz, 1H), 7.37 (q, J = 3.0 Hz, 3H), 7.29-7.18 (m, 8H), 7.14-7.09 (m, 1H).

3.2. Synthesis of (*R*, *R*)-**P5**

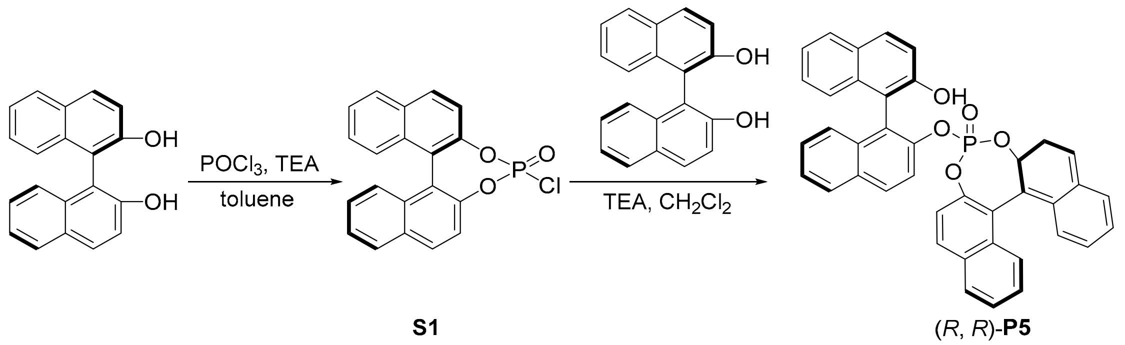

S1 (*R*, *R*)-**P5**

Preparation of chlorophosphonate **S1**

The (*R*)-(+)-1,1'-bi-2-naphthol (5 g, 17.5 mmol) and Et_3N (10 ml, 70 mmol) was dissolved in dry toluene (90 ml) and $POCl_3$ (2.9 g, 19 mmol) was added dropwisely under argon at 0 °C. The reaction was stirred at room temperature overnight. The solid was removed by filtration. The filtrate was concentrated and purified by flash column chromatography (EtOAc/PE) to give product **S1** as white solid (4.99 g, 78 % yield).

Preparation of the ligand (*R*, *R*)-**P5**

Under a nitrogen atmosphere, to a solution of **S1** (3.0 g, 8 mmol) and (*R*)-(+)-1,1'-bi-2-naphthol (2.3 g, 8mmol) in anhydrous CH_2Cl_2 (50 ml), Et_3N (1.2 mL, 8 mmol) was added at 0 °C. The reaction was stirred at room temperature overnight. The solvent was removed under vacuum and the residue was purified by flash column chromatography (CH_2Cl_2/PE) to give product (*R*, *R*)-**P5** as white solid (3.44 g, 70 % yield). ^{1}H NMR (400 MHz, DMSO-d_6) δ 9.58 (s, 1H), 8.24 (d, J = 8.9 Hz, 1H), 8.18 (s, 1H), 8.06 (dt, J = 13.3, 7.1 Hz, 3H), 7.89 (d, J = 8.6 Hz, 2H), 7.82 (d, J = 9.7 Hz, 2H), 7.71 (d, J = 8.9 Hz, 1H), 7.50 (d, J = 7.6 Hz, 3H), 7.35-7.29 (m, 4H), 7.25 (d, J = 7.5 Hz, 1H), 7.19-7.12 (m, 4H), 6.98 (d, J = 8.3 Hz, 1H), 6.11 (d, J = 8.9 Hz, 1H); ^{13}C NMR (100 MHz, DMSO-d_6) δ 153.8, δ 146.9 (d, J = 11.7 Hz), 146.4 (d, J = 6.4 Hz), 146.0 (d, J = 8.4 Hz), 134.1, 133.8, 132.3, 132.0, 131.8 (d, J = 5.4 Hz), 131.7, 131.6 (d, J = 3.1 Hz), 130.1, 129.2, 128.7 (d, J = 9.9 Hz), 128.4, 127.8, 127.5 (d, J = 9.2 Hz), 127.0, 126.7, 126.6, 126.4, 126.2 (d, J = 5.0 Hz), 124.2, 124.14, 123.2, 121.2, 121.1 (d, J = 6.7 Hz), 121.0, 120.2, 120.1, 120.1 (d, J = 8.3 Hz), 119.6, 118.7, 113.2; ^{31}P NMR (161 MHz, DMSO-d_6) δ -2.78 . HRMS (ESI) calcd for [$C_{40}H_{35}NaO_5P$, M+Na]$^+$: 639.1332, Found: 639.1333.

3.3. Synthesis of (*S*, *R*)-**P6**

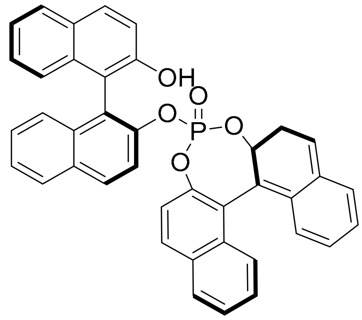

(*S*, *R*)-**P6**

^{1}H NMR (400 MHz, DMSO-d_6) δ 9.75 (s, 1H), 8.19 (t, J = 8.7 Hz, 2H), 8.08 (t, J = 7.2 Hz, 2H), 7.98 (d, J = 8.3 Hz, 1H), 7.87 (t, J = 8.8 Hz, 2H), 7.81 (d, J = 9.0 Hz, 1H), 7.69 (d, J = 8.9 Hz, 1H), 7.51 (t, J = 7.8 Hz, 3H), 7.43 (d, J = 8.9 Hz, 1H), 7.34 (t, J = 8.8 Hz, 4H), 7.22-7.13 (m, 3H), 7.05 (d, J = 8.8 Hz, 2H), 6.78 (d, J = 8.5 Hz, 1H), 6.47 (d, J = 8.9 Hz, 1H); ^{13}C NMR (100 MHz, DMSO-d_6) δ 153.6, δ 146.9 (d, J = 11.7 Hz), 146.3 (d, J = 6.5 Hz), 145.9 (d, J = 8.3 Hz), 134.2, 133.7, 132.2, 131.9, 131.7 (d, J = 4.5 Hz), 131.6, 131.5, 130.2, 129.2 (d, J = 9.1 Hz), 128.7, 128.3 (d, J = 13.0 Hz), 127.8, 127.5 (d, J = 7.4 Hz), 126.7, 126.6, 126.6, 126.5, 126.2, 124.5 (d, J = 7.6 Hz), 124.4, 123.0, 121. 0, 120.8, 120.2, 119.7, 118.7, 113.2; ^{31}P NMR (161 MHz, DMSO-d_6) δ -2.59. HRMS (ESI) calcd for [$C_{40}H_{35}NaO_5P$, M+Na]$^+$: 639.1332, Found: 639.1328.

3.4. Synthesis of (*S*, *R*)-**P7**

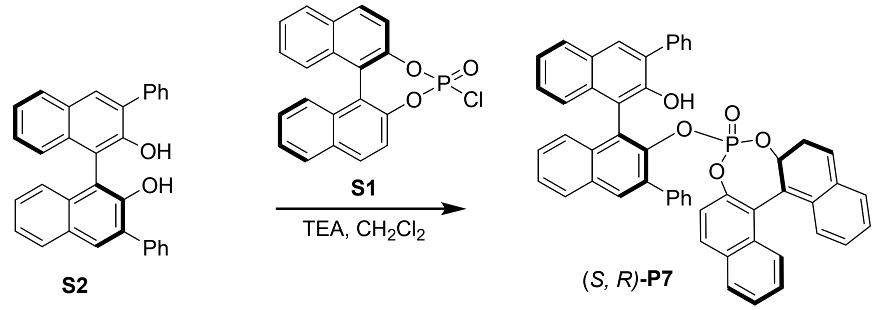

Under a nitrogen atmosphere, to a solution of **S1** (36.6 mg, 0.1 mmol) and **S2** (43.8 mg, 0.1 mmol) in anhydrous CH_2Cl_2 (10 ml), Et_3N (30 mg, 0.3 mmol) was added at 0 °C. The reaction was stirred at room temperature overnight. The solvent was removed under vacuum and the residue was purified by flash column chromatography (CH_2Cl_2/PE) to give **P7** as a white solid (616 mg, 80 % yield). ^{1}H NMR (400 MHz, $CDCl_3$) δ 8.10 (t, J = 9.3 Hz, 2H), 7.90 (d, J = 8.2 Hz, 1H), 7.87-7.79 (m, 3H), 7.71 (d, J = 7.9 Hz, 1H), 7.52 (m, 3H), 7.44-7.37 (m, 2H), 7.24 (m, 5H), 7.15 - 7.09 (m, 1H), 6.98 (m, 3H), 6.88 (d, J = 5.8 Hz, 1H), 6.80 (m, 5H), 6.60-6.53 (m, 1H), 6.28 (m, 5H). ^{13}C NMR (100 MHz, $CDCl_3$) δ 151.4, 146.3, δ 146.2 (d, J = 8.2 Hz), 144.4 (d, J = 11.7 Hz), 144.3, 143.5 (d, J = 8.9 Hz), 143.4, 138.9, 138.6, 138.5, 138.4, 133.3, 133.0, 132.9 (d, J = 2.0 Hz), 132.8, 131.1, 130.3, 129.4, 128.2, 127.9, 127.8, 127.2, 127.0, 126.8, 126.8, 126.7, 126.6, 126.5, 126.4, 125.7, 125.6, 125.6 (d, J = 2.2 Hz), 125.5, 125.3, 125.1, 124.4 (d, J = 2.6 Hz), 124.3, 123.7, 123.6, 1123.5 (d, J = 3.5 Hz), 122.9, 122.8, 122.7, 121.6 (d, J = 2.0 Hz), 121.0, 120.7 (d, J = 2.1 Hz). 120.4, 119.3, 118.5, 114.7. ^{31}P NMR (161 MHz, $CDCl_3$) δ -0.28.

3.5 Synthesis of (*S*, *R*)-**P8**

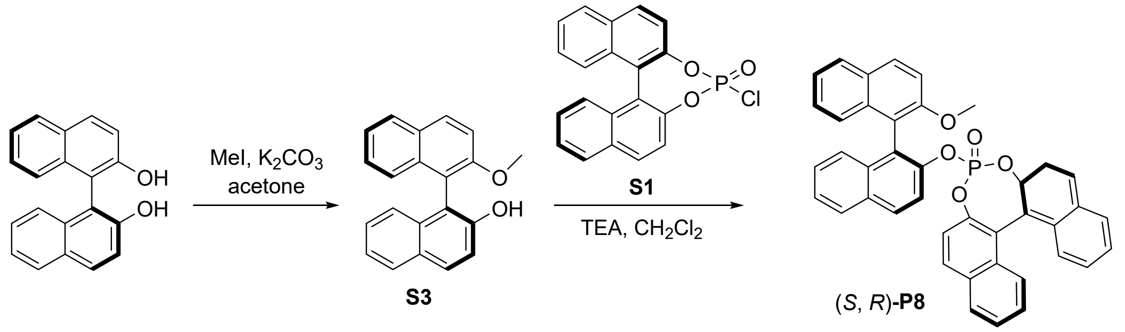

Preparation of **S3**:

The (*S*)-(+)-1,1'-bi-2-naphthol (1.43 g, 5 mmol) and K_2CO_3 (828 mg, 6 mmol) was dissolved in dry acetone (40 ml) and MeI (775 mg, 5.5 mmol) was added dropwisely under nitrogen atmosphere at 30 °C. The slurry was stirred for 18 h under reflux. The solid was removed by filtration. The filtrate was concentrated and purified by flash column chromatography (EtOAc/PE) to delivered product **S3** as a white solid (1.47 g, 98 % yield). 1H NMR (400 MHz, $CDCl_3$) δ 8.04 (d, J = 9.0 Hz, 1H), 7.92-7.90 (m, 2H), 7.86 (d, J = 8.1 Hz, 1H), 7.49 (d, J = 9.1 Hz, 1H), 7.39-7.28 (m, 4H), 7.22 (ddd, J = 8.2, 6.8, 1.3 Hz, 1H), 7.17 (d, J = 8.1 Hz, 1H), 7.05 (d, J = 8.4 Hz, 1H), 3.81 (s, 3H). ^{13}C NMR (100 MHz, $CDCl_3$) δ 156.1, 151.3, 134.1, 133.9, 131.2, 129.9, 129.5, 129.3, 128.3, 128.3, 127.5, 126.5, 125.0, 124.9, 124.3, 123.4, 117.6, 115.4, 115.1, 113.9, 56.8.

Preparation of (*S*, *R*)-**P8**

Under a nitrogen atmosphere, to a solution of **S1** (366 mg, 1 mmol) and **S3** (300 mg, 1 mmol) in anhydrous CH_2Cl_2 (10 ml), Et_3N (0.45 ml, 3 mmol) was added at 0 °C. The reaction was stirred at room temperature overnight. The solvent was removed under vacuum and the residue was purified by flash column chromatography (CH_2Cl_2/PE) to delivered product (*S*, *R*)-**P8** as a white solid (505 mg, 80 % yield). ^{1}H NMR (400 MHz, $CDCl_3$) δ 7.80 (d, J = 9.0 Hz, 1H), 7.68 m, 3H), 7.58 (dd, J = 12.4, 8.2 Hz, 2H), 7.46 (m, 2H), 7.26 (d, J = 8.2 Hz, 2H), 7.20-7.15 (m, 2H), 7.12 (d, J = 7.6 Hz, 1H), 7.06-6.99 (m, 4H), 6.93 (dt, J = 11.7, 7.5 Hz, 4H), 6.86-6.78 (m, 2H), 6.09 (d, J = 8.8 Hz, 1H), 3.54 (s, 3H).^{13}C NMR (100 MHz, $CDCl_3$) δ 171.0, 155.0, 147.0, 146.8, 146.0, 133.8, 133.7, 132.0, 131.7, 131.6, 131.5, 131.4, 131.2, 130.6, δ 129.9 (d, J = 5.8 Hz), 129.8, 129.8, 128.5, 128.4, 128.3 (d, J = 11.1 Hz), 128.2, 128.1, 127.6, 127.1, 126.8, 126.6, 126.4, 126.3 (d, J = 9.7 Hz),, 126.1, 125.6 (d, J = 10.3 Hz), 125.5, 123.3, 121.1, 120.5, 120.4, 119.9, 119.6, 116.8, 113.3, 56.4. ^{31}P NMR (161 MHz, $CDCl_3$) δ -2.76.

3.6 Synthesis of (*R*, *R*)-**P9**[5]

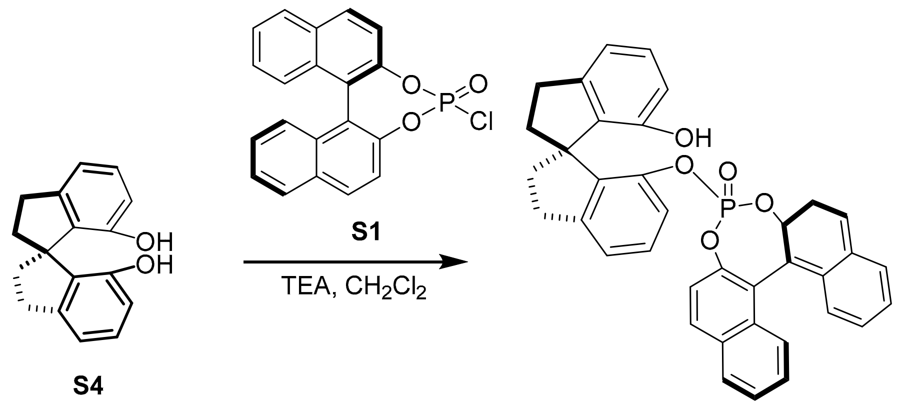

(*R*, *R*)-**P9**

Under a nitrogen atmosphere, to a solution of **S1** (3 g, 8 mmol) and (*R*)-2,2',3,3'-tetrahydro-1,1'-spirobi[1H-indene]-7,7'-diol (**S4**, 2 g, 8mmol) in anhydrous CH_2Cl_2 (50 ml), Et_3N (1.2 mL, 8 mmol) was added at 0 °C. The reaction was stirred at room temperature overnight. The solvent was removed under vacuum and the residue was purified by flash column chromatography (CH_2Cl_2/PE) to delivered product **P9** as a white solid (3.3 g, 72 % yield). ^{1}H NMR (400 MHz, $CDCl_3$) δ 8.06 (d, J = 9.0 Hz, 1H), 7.96 (t, J = 8.8 Hz, 2H), 7.91 (d, J = 8.9 Hz, 1H), 7.65 (d, J = 8.9 Hz, 1H), 7.50 (t, J = 7.4 Hz, 2H), 7.32 (td, J = 14.2, 6.1 Hz, 6H), 7.16 (d, J = 7.3 Hz, 1H), 6.93 (s, 2H), 6.66 (d, J = 8.8 Hz, 1H), 6.52 (d, J = 5.7 Hz, 1H), 3.14-3.01 (m, 3H), 2.93 (dd, J = 15.8, 8.6 Hz, 1H), 2.30 (q, J = 7.4, 5.0 Hz, 2H), 2.20 (dd, J = 12.5, 7.3 Hz, 1H), 2.06 (d, J = 12.1 Hz, 1H); ^{13}C NMR (100 MHz, $CDCl_3$) δ 153.2, 147.6 (d, J = 1.7 Hz), 147.5 (d, J = 2.6 Hz), 147.3, δ146.6 (d, J = 8.9 Hz), 145.3, 138.1 (d, J = 8.5 Hz), 135.1, 132.6, 132.4, 132.3, 132.1, 131.9, 131.3, 129.0, 128.9, 128.8, 127.5, 127.4, 127.21, 127.0, 126.3, 126.2, 122.5, 117.4, 121.9 (d, J = 2.2 Hz), 121.1 (d, J = 2.1 Hz), 120.9 (d, J = 2.8 Hz), 120.7 (d, J = 3.2 Hz), 117.2, 116.2, 59.2, 38.1, 37.8, 31.6 (d, J = 6.2 Hz); ^{31}P NMR (162 MHz, $CDCl_3$) δ -2.02 . HRMS (ESI) calcd for $[C_{37}H_{27}NaO_5P, M+Na]^+$: 605.1488,

Found: 605.1474.

3.7 Synthesis of (*S*, *R*)-**P10**

The (*S*, *R*)-**P10** was prepared according to the (*R*, *R*)-**P9** procedure.

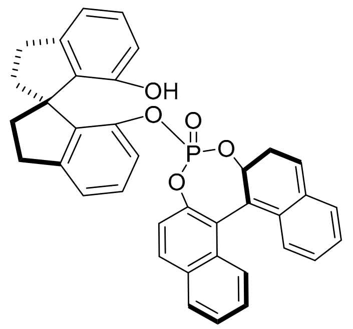

(*S*, *R*)-**P10**

^{1}H NMR (400 MHz, $CDCl_3$) δ 7.97 (d, *J* = 8.9 Hz, 1H), 7.93 - 7.87 (m, 2H), 7.83 (s, 1H), 7.45 (t, *J* = 8.1 Hz, 3H), 7.29 (m, 6H), 7.15 (d, *J* = 7.3 Hz, 1H), 7.00 (t, *J* = 7.7 Hz, 1H), 6.85 (d, *J* = 8.9 Hz, 1H), 6.59 (dd, *J* = 15.6, 7.7 Hz, 2H), 3.03 (t, *J* = 7.3 Hz, 2H), 2.89 (dd, *J* = 16.6, 7.6 Hz, 1H), 2.80 (dd, *J* = 15.7, 8.6 Hz, 1H), 2.30 (d, *J* = 9.9 Hz, 1H), 2.21 (dt, *J* = 22.2, 7.6 Hz, 3H); ^{13}C NMR (100MHz, $CDCl_3$) δ 152.5, 147.4 (d, *J* = 6.7 Hz), 147.4, 147.3 (d, *J* = 11.5 Hz), 146.1 (d, *J* = 8.5 Hz), 145.1, 137.8 (d, *J* = 7.3 Hz), 134.6, 132.2, 131.9, 131.7, 131.5, 131.1, 129.2, 128.5, 128.4, 128.2, 127.3, 127.0, 126.7, 126.6, 125.8 (d, *J* = 6.1 Hz), 122.6, 121.4 (d, *J* = 2.4 Hz), 120.9 (d, *J* = 2.1 Hz), 120.7 (d, *J* = 3.1 Hz), 120.2 (d, *J* = 3.3 Hz), 117.9, 117.4, 115.3, 58.8, 38.0, 37.8, 31.3, 31.1; ^{31}P NMR (161 MHz, $CDCl_3$) δ -1.91. HRMS (ESI) calcd for [$C_{37}H_{27}NaO_5P$, M+Na]$^+$: 605.1488, Found: 605.1480.

3.8 Synthesis of (*S*, *S*, *S*, *R*)-**P11**

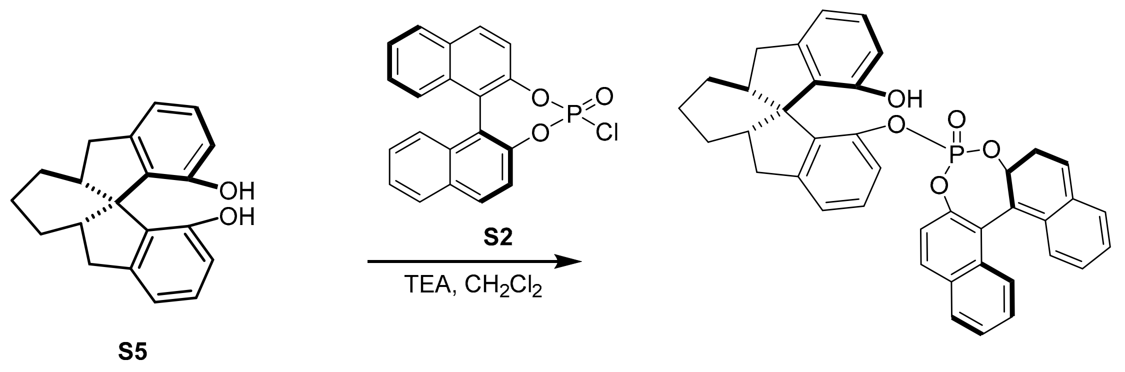

(*S*, *S*, *S*, *S*)-**P11**

Under a nitrogen atmosphere, to a solution of **S2** (366 mg, 1 mmol) and cyclohexyl-fused chiral spirobiindanediol[6] **S5** (292 mg, 1 mmol) in anhydrous CH_2Cl_2 (8 ml), Et_3N (0.15 mL, 1 mmol) was added at 0 °C. The reaction was stirred at room temperature overnight. The solvent was removed under vacuum and the residue was purified by flash column chromatography (CH_2Cl_2/PE) to delivered product **P11** as a white solid (466 mg, 75 % yield). ^{1}H NMR (400 MHz, $CDCl_3$) δ 8.01 (d, *J* = 8.9 Hz, 1H), 7.94 (t, *J* = 7.9 Hz, 2H), 7.85 (d, *J* = 8.9 Hz, 1H), 7.57 (d, *J* = 8.9 Hz, 1H), 7.53-7.43 (m, 3H), 7.30 (m, 5H), 7.16 (d, *J* = 7.4 Hz, 1H), 7.06 (d, *J* = 8.9 Hz, 1H), 6.87 (t,

J = 7.6 Hz, 1H), 6.76 (d, *J* = 7.3 Hz, 1H), 6.35 (d, *J* = 7.9 Hz, 1H), 3.15 (dd, *J* = 15.9, 7.5 Hz, 1H), 2.73 (dd, *J* = 15.5, 6.1 Hz, 2H), 2.65 (s, 1H), 2.57 (s, 1H), 2.29 (dd, *J* = 15.1, 7.6 Hz, 1H), 1.47 (m, 4H), 1.28-1.19 (m, 2H); ^{13}C NMR (100 MHz, $CDCl_3$) δ 152.7, δ 148.3 (d, *J* = 6.6 Hz), 147.7, 147.1 (d, *J* = 11.6 Hz), 146.2 (d, *J* = 8.7 Hz), 144.9, 135.7 (d, *J* = 8.1 Hz), 133.3, 132.2 (d, *J* = 7.7 Hz), 131.8, 131.6, 131.5, 131.1, 129.2, 128.5 (d, *J* = 8.9 Hz), 128.0, 127.1, 126.9, 126.8 (d, *J* = 6.9 Hz), 125.9, 123.0, 121.4 (d, *J* = 2.3 Hz), 120.9 (d, *J* = 2.3 Hz), 120.5 (d, *J* = 2.9 Hz), 120.0 (d, *J* = 3.4 Hz), 118.3, 117.2, 115.3, 61.1, 44.4, 43.0, 37.8, 36.0, 25.8, 23.5, 17.2; ^{31}P NMR (161 MHz, $CDCl_3$) δ -2.93. HRMS (ESI) calcd for [$C_{40}H_{31}NaO_5P$, M+Na]$^+$: 645.1801, Found: 645.1791.

4. Deuteration of styrene

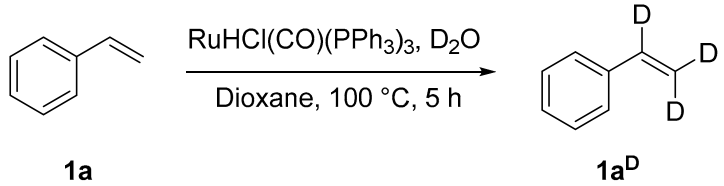

A mixture of $RuHCl(CO)(PPh_3)_3$ (38.1 mg, 0.04 mmol, 2.0 mol%), styrene (230 μL, 2.0 mmol) and D_2O(1 ml) in dioxane (4 mL) in a stoppered Schlenck tube was stirred and heated at 100 °C for 5 hours. The reaction mixture was cooled to room temperature and then extracted with diethyl ether. The combined organic extract was washed with water and a brine solution and dried over $MgSO_4$. The crude product was purified by column chromatography on silica gel using n-hexane as the eluent to give the desired product **1a^{D}** as a colorless oil (128 mg, 60% yield). Theoretical percentage of deuteration at the vinyl position = 97%. ^{1}H NMR (400 MHz, $CDCl_3$) δ 7.43-7.41 (m, 2H), 7.36-7.31(m, 2H), 7.28-7.24 (m, 1H), 6.71 (brs, 0.03H), 5.74-5.73 (m, 0.03H), 5.23(m, 0.03H).

5. General procedure for hydroformylation

The hydroformylation reactions were conducted in a batch reactor (Shanghai Yanzheng). In a typical run, 0.001 mmol of chloro(1,5-cyclooctadiene)rhodium(I) dimer, 0.006 mmol of ligand (Rh/L= 1:3) was dissolved in 25 mL toluene. and then the solution of substrate (3.0 mmol) was added. After that, the reactor was charged with 4.0 MPa syngas (CO/H_2=1:1) for 12-48 h at 30 °C. The products were analyzed with GC and GC-MS. The yield and the regioselectivity of aldehydes were identified by GC. The mixture was concentrated under reduced pressure. Then the crude product was purified by flash chromatography on silica gel to give the desired aldehyde.

6. Characterization data of *rac*-*α*-aryl propionaldehydes[7-16]

2-(2-Fluorophenyl)propanal (2b)

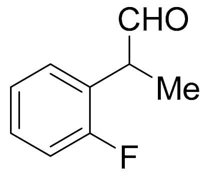

Colorless oil, 94% yield. ^{1}H NMR (400 MHz, $CDCl_3$) δ 9.67 (d, J = 0.7 Hz, 1H), 7.22 (m, 1H), 7.11-7.01 (m, 3H), 3.84 (q, J = 7.1 Hz, 1H), 1.38 (d, J = 7.2 Hz, 3H).

2-(3-Fluorophenyl)propanal (2c)

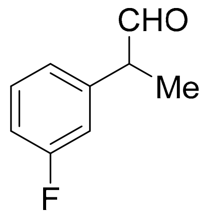

Colorless oil, 94% yield. ^{1}H NMR (400 MHz, $CDCl_3$) δ 9.56 (d, J = 1.4 Hz, 1H), 7.27-7.21 (m, 1H), 6.92-6.87 (m, 2H), 6.85-6.81 (m, 1H), 3.54 (q, J = 6.4 Hz, 1H), 1.34 (d, J = 7.1 Hz, 3H).

2-(4-fluorophenyl)propanal (2d)

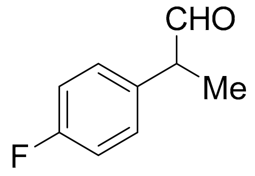

Colorless oil, 93% yield. ^{1}H NMR (400 MHz, $CDCl_3$) δ 9.67 (d, J = 1.3 Hz, 1H), 7.38-7.34 (m, 2H), 7.18-7.14 (m, 2H), 3.64 (q, J = 6.7 Hz, 1H), 1.45 (d, J = 7.1 Hz, 3H).

2-(2-chlorophenyl)propanal (2e)

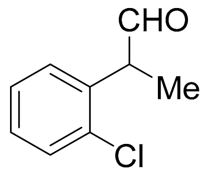

Colorless oil, 95% yield. ^{1}H NMR (400 MHz, $CDCl_3$) δ 9.72 (s, 1H), 7.44 (dd, J = 7.5, 1.8 Hz, 1H), 7.31-7.22 (m, 2H), 7.14 (dd, J = 7.3, 2.1 Hz, 1H), 4.14 (q, J = 7.1 Hz, 1H), 1.44 (d, J = 7.1 Hz, 3H).

2-(3-chlorophenyl)propanal (2f)

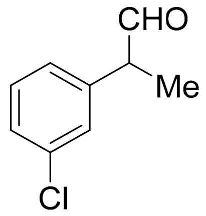

Colorless oil, 94% yield. ^{1}H NMR (400 MHz, $CDCl_3$) δ 9.68 (d, J = 1.3 Hz, 1H), 7.35-7.28 (m, 2H), 7.23 (d, J = 1.9 Hz, 1H), 7.11 (m, 1H), 3.64 (q, J = 6.7 Hz, 1H), 1.46 (d, J = 7.1 Hz, 3H).

2-(4-Chlorophenyl)propanal (2g)

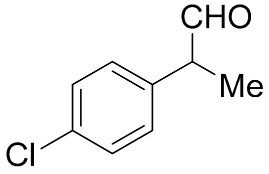

Colorless oil, 93% yield. ^{1}H NMR (400 MHz, $CDCl_3$) δ 9.64 (d, J = 1.3 Hz, 1H), 7.36-7.31 (m, 2H), 7.16-7.12 (m, 2H), 3.62 (q, J = 7.1 Hz, 1H), 1.42 (d, J = 7.1 Hz, 3H).

2-(2-bromophenyl)propanal (2h)

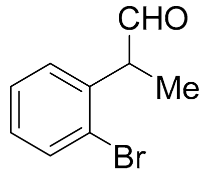

Colorless oil, 91% yield. ^{1}H NMR (400 MHz, $CDCl_3$) δ 9.65 (s, 1H), 7.55 (dd, J = 8.0, 1.3 Hz, 1H), 7.27-7.22 (m, 1H), 7.09 (m, 1H), 7.03 (dd, J = 7.7, 1.7 Hz, 1H), 4.08 (q, J = 7.1 Hz, 1H), 1.34 (d, J = 7.1 Hz, 3H).

2-(3-bromophenyl)propanal (2i)

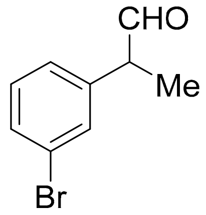

Colorless oil, 94% yield. ^{1}H NMR (400 MHz, $CDCl_3$) δ 9.50 (d, J = 1.3 Hz, 1H), 7.29-7.23 (m, 2H), 7.10 (t, J = 7.8 Hz, 1H), 7.00 (m, 1H), 3.47 (q, J = 7.1, 6.4 Hz, 1H), 1.28 (d, J = 7.1 Hz, 3H).

2-(4-bromophenyl)propanal (2j)

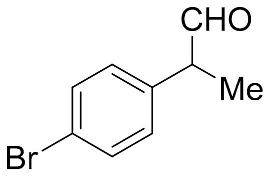

Colorless oil, 93% yield.^{1}H NMR (400 MHz, $CDCl_3$) δ 9.66 (d, J = 1.3 Hz, 1H), 7.53-7.47 (m, 2H), 7.12-7.05 (m, 2H), 3.62 (q, J = 7.1, 6.7 Hz, 1H), 1.44 (d, J = 7.1 Hz, 3H).

2-(4-nitrophenyl)propanal (2k)

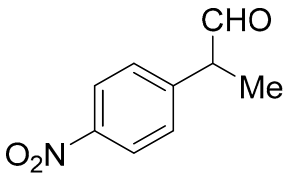

Yellow solid, m.p. 39.5-40.1 °C, 94% yield.^{1}H NMR (400 MHz, $CDCl_3$) δ 9.65 (d, J = 1.1 Hz, 1H), 8.17 (d, J = 8.7 Hz, 2H), 7.33 (d, J = 8.7 Hz, 2H), 3.73 (q, J = 7.1 Hz, 1H), 1.45 (d, J = 7.2 Hz, 3H).

2-(*o*-Tolyl)propanal (2l)

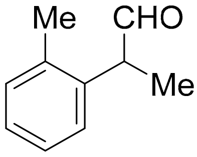

Colorless oil, 92% yield.^{1}H NMR (400 MHz, $CDCl_3$) δ 9.52 (s, 1H), 7.12-7.06 (m, 3H), 6.92 (d, J = 6.4 Hz, 1H), 3.72 (q, J = 7.0 Hz, 1H), 2.24 (s, 3H), 1.29 (d, J = 8.3 Hz, 3H).

2-(*m*-Tolyl)propanal (2m)

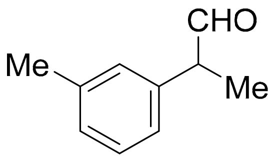

Colorless oil, 92% yield. ^{1}H NMR (400 MHz, $CDCl_3$) δ 9.59 (d, J = 1.0 Hz, 1H), 7.18 (t, J = 7.9 Hz, 1H), 7.03 (d, J = 7.5 Hz, 1H), 6.92 (d, J = 6.6 Hz, 2H), 3.51 (q, J = 7.0 Hz, 1H), 2.27 (s, 3H), 1.34 (d, J = 7.1 Hz, 3H).

2-(*p*-Tolyl)propanal (2n)

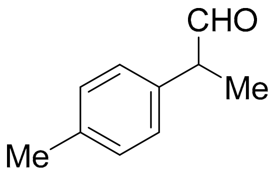

Colorless oil, 92% yield. ^{1}H NMR (400 MHz, $CDCl_3$) δ 9.66 (d, J = 1.3 Hz, 1H), 7.20 (d, J = 7.9 Hz, 2H), 7.10 (d, J = 8.0 Hz, 2H), 3.60 (q, J = 6.9 Hz, 1H), 2.35 (s, 3H), 1.42 (d, J = 7.1 Hz, 3H).

2-(4-*iso*-Butylphenyl)propanal (2o)

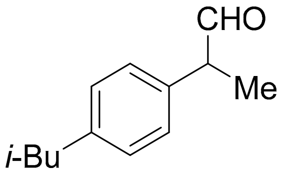

Colorless oil, 92% yield.^{1}H NMR (400 MHz, $CDCl_3$) δ 9.55 (d, J = 1.4 Hz, 1H), 7.06-6.98 (m, 4H), 3.49 (q, J = 6.1 Hz, 1H), 2.36 (d, J = 7.2 Hz, 2H), 1.75 (m, 1H), 1.31 (d, J = 7.1 Hz, 3H), 0.80 (d, J = 6.6 Hz, 6H).

2-(4-(*tert*-Butyl)phenyl)propanal (2p)

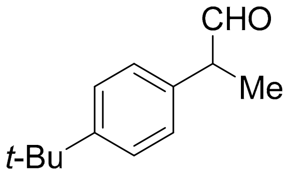

Colorless oil, 88% yield.^{1}H NMR (400 MHz, $CDCl_3$) δ 9.71 (d, J = 1.3 Hz, 1H), 7.44 (d, J = 8.3 Hz, 2H), 7.19 (d, J = 8.2 Hz, 2H), 3.65 (q, J = 7.0 Hz, 1H), 1.47 (d, J = 7.1 Hz, 3H), 1.36 (s, 9H).

2-(4-Hydroxyphenyl)propanal (2q)

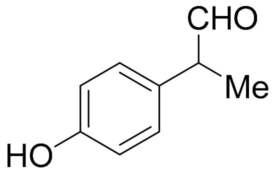

Colorless oil, 92% yield.^{1}H NMR (400 MHz, $CDCl_3$) δ 9.53 (d, J = 1.0 Hz, 1H), 6.96 (d, J = 8.4 Hz, 2H), 6.77 (d, J = 8.4 Hz, 2H), 6.57 (s, 1H), 3.50 (q, J = 6.8 Hz, 1H), 1.31 (d, J = 7.1 Hz, 3H).

2-(4-Methoxyphenyl)propanal (2r)

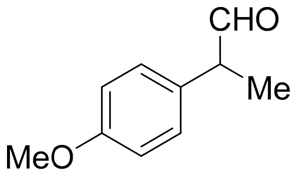

Colorless oil, 91% yield.^{1}H NMR (400 MHz, $CDCl_3$) δ 9.53 (d, J = 1.4 Hz, 1H), 7.05-7.00 (m, 2H), 6.84-6.79 (m, 2H), 3.69 (s, 3H), 3.48 (q, J = 7.1 Hz, 1H).

2-(2,5-dimethylphenyl)propanal (2s)

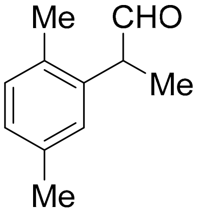

Colorless oil, 91% yield.^{1}H NMR (400 MHz, $CDCl_3$) δ 9.71 (d, J = 1.1 Hz, 1H), 7.19 (d, J = 7.7 Hz, 1H), 7.08 (d, J = 9.1 Hz, 1H), 6.92 (s, 1H), 3.91-3.84 (m, 1H), 2.39 (s, 3H), 2.38 (s, 3H), 1.46 (d, J = 7.0 Hz, 3H).

2-(3,4-dimethoxyphenyl)propanal (2t)

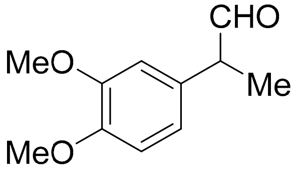

Colorless oil, 89% yield.^{1}H NMR (400 MHz, $CDCl_3$) δ 9.54 (d, J = 1.4 Hz, 1H), 6.78 (d, J = 8.2 Hz, 1H), 6.66 (dd, J = 8.2, 2.0 Hz, 1H), 6.60 (d, J = 2.0 Hz, 1H), 3.77 (s, 3H), 3.77 (s, 3H), 3.51-3.44 (m, 1H), 1.32 (d, J = 7.1 Hz, 3H).

2-(naphthalen-2-yl)propanal (2u)

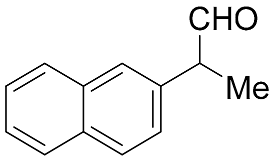

White solid, m.p. 87.0-88.2 °C, 92% yield.^{1}H NMR (400 MHz, $CDCl_3$) δ 9.80 (d, J = 1.4 Hz, 1H), 7.92-7.86 (m, 3H), 7.72 (s, 1H), 7.60-7.51 (m, 2H), 7.36 (dd, J = 8.4, 1.8 Hz, 1H), 3.82 (q, J = 6.6 Hz, 1H), 1.59 (d, J = 7.1 Hz, 3H).

7. Isotope Effect Measurements.

Scheme S1 Competition Isotope Effect of deuterated styrene.

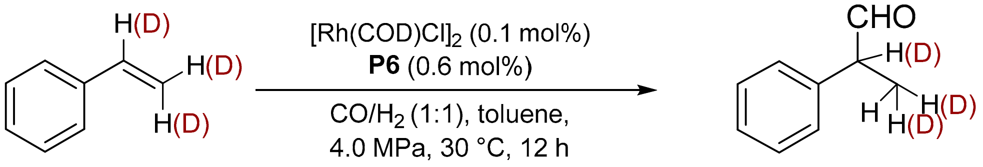

1a/1aD 2a/2a^D

The Competition Isotope Effect was calculated using the following equation[17, 18]:

$(1\text{-}F_H) = \chi_t^{2a}(1\text{-}F_{tot})/\ \chi_0^{1a}$

$kH/kD = ln(1\text{-}F_H)/ln[R(1\text{-}F_H)/R_0]$

where F_{tot} represent the total conversion; F_H represent fractional conversion of protiated starting material; R_0 and R represent the extent of deuterium incorporation in the starting material prior to and following the reaction, respectively; χ_0 and χ_t represent initial and final mole fraction, respectively.

Table S2 Competition isotope effect of deuterated styrene.[a]

χ_0^{1a}	χ_0^{1aD}	R_0	χ_t^{2a}	χ_t^{2aD}	R	R/R_0	$1\text{-}F_{tot}$	$1\text{-}F_H$	k_H/k_D
0.55	0.45	1.22	0.66	0.34	1.94	1.59	0.45	0.28	1.57

[a] Deuterated product ratio was determined from integration of the ^{1}H NMR signals.

Scheme S2 Competition isotope effect under H_2/D_2 atmosphere.[a]

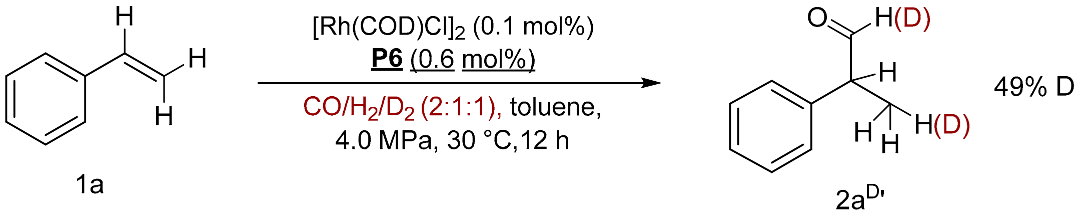

1a 2a$^{D'}$

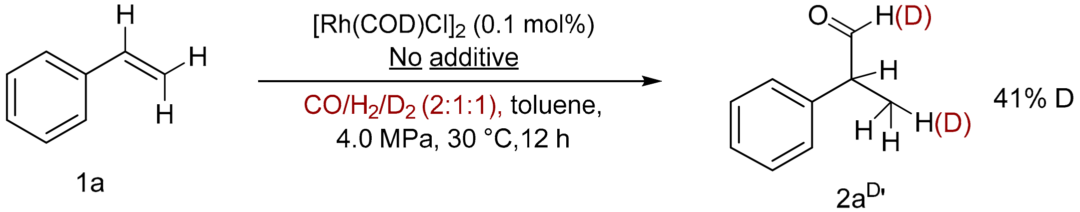

1a 2a$^{D'}$

[a] Deuterated product ratio was determined from integration of the ^{1}H NMR signals.

8. References

[1] A. Ngo Ndimba, T. Roisnel, G. Argourch, C. Lalli, *Synthesis,* **2019**, *51*, 865-873.

[2] V. Grushin, *Chem. Rev.* **2004,** *104,* 1629-1662.

[3] B. Q. Gong, W. Y. Chen, B. F. Hu, *J. Org. Chem*. **1991**, *56*, 423-425.

[4] J. G. An, J. M. Wilson, Y. Z. An, D. F. Wiemer, *J. Org. Chem.* **1996**, *61,* 4040-4045.

[5] B. Xu, S. F. Zhu, X. L. Xie, J. J. Shen, Q. L. Zhou, *Angew. Chem. Int. Ed*. **2011**, *50*, 11483-11486.

[6] Z. Y. Zheng, Y. X. Cao, Q. L. Chong, Z. B. Han, J. M. Ding, C. G. Luo, Z. Wang, D. S. Zhu, Q. L. Zhou, K. L. Ding, *J. Am. Chem. Soc*. **2018**, *140*, 10374-10381.

[7] R. Witten, N. Jacobsen, *Org. Lett.* **2015**, *17*, 2772–2775.

[8] R. Tanaka, K. Nakano, K. Nozaki, *J. Org. Chem.* **2007**, *72*, 8671-8676.

[9] J. Zhang, C. Liu, G. Wang, Z. Chen, F. Zhang, B. Zhang, *Chem. Commun.* **2018**, *54*, 6204.

[10] H. Kikuchi, K. Kogure, M. Toyoda. *Chemistry Letters*. **1984**, 341-344.

[11] S. Kasinathan, L. Bourne, P. Tolstoy, P. Koos, Matthew. Brien, W. Bates, R. Baxendale, V. Ley, *Synlett*. **2011**, *18*, 2648-2651.

[12] B. List, I. Čorić, O. Grygorenko, J. Kaib, I. Komarov, A. Lee, M. Leutzsch, S. Chandra, V. Tymtsunik, V. Gemmeren. *Angew. Chem. Int. Ed.* **2014**, *53*, 282-285.

[13] J. Zhang, W. Schuppe, T. Pan, X. Chen, R. Wang, R. Newhouse, L. Yin. *J. Am. Chem. Soc.* **2018**, *140*, 5300-5310.

[14] A. Reziq, H. Alper, S. Wang, L. Post, *J. Am. Chem. Soc.* **2006**, *128*, 5279-5282.

[15] L. Zhang, Z. Zuo, X. Wan, Z. Huang, *J. Am. Chem. Soc.* **2014**, *136*, 15501-15004.

[16] J. Fried, *J. Med. Chem.* **1970**, *13*, 203-205.

[17] L. C. S. Melander; W. H. Saunders, *Reaction Rates of Isotopic Molecules*; Wiley: New York, **1980**; p 102.

[18] D. A. Singleton; A. A. Thomas, *J. Am. Chem. Soc.* **1995**, *117*, 9357-9358.

9. IR spectra

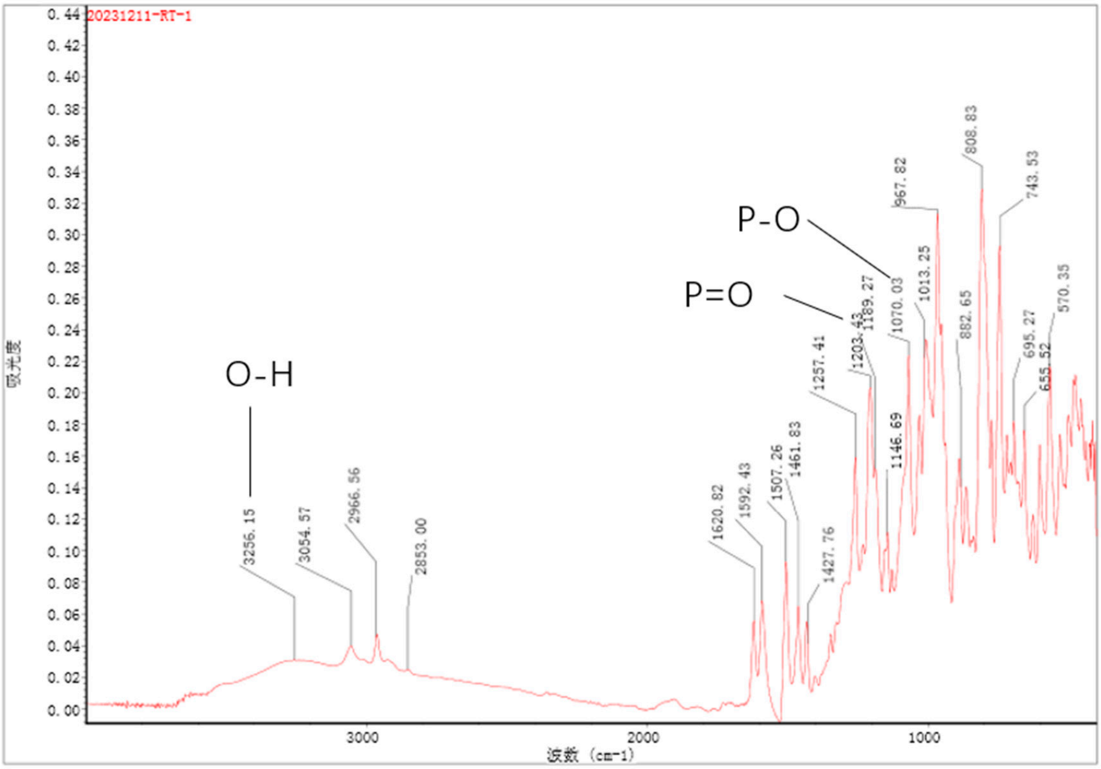

Fig. S4 IR spectrum of **P6**

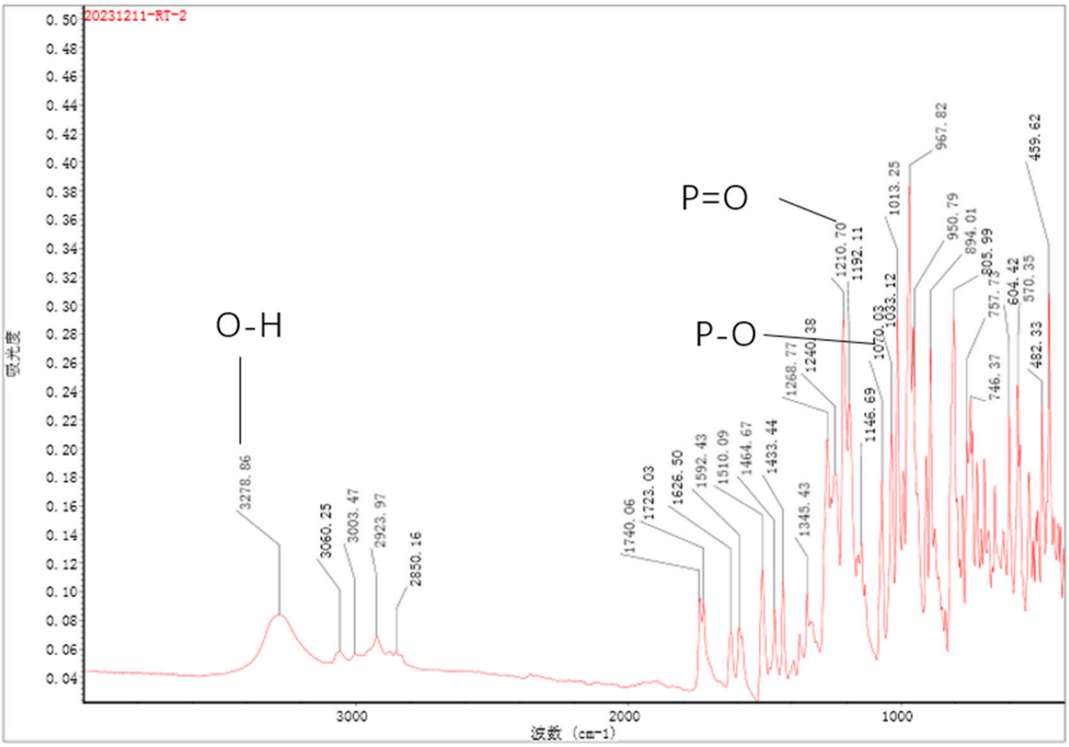

Fig. S5 IR spectrum of **P6**/[Rh] = 1/1

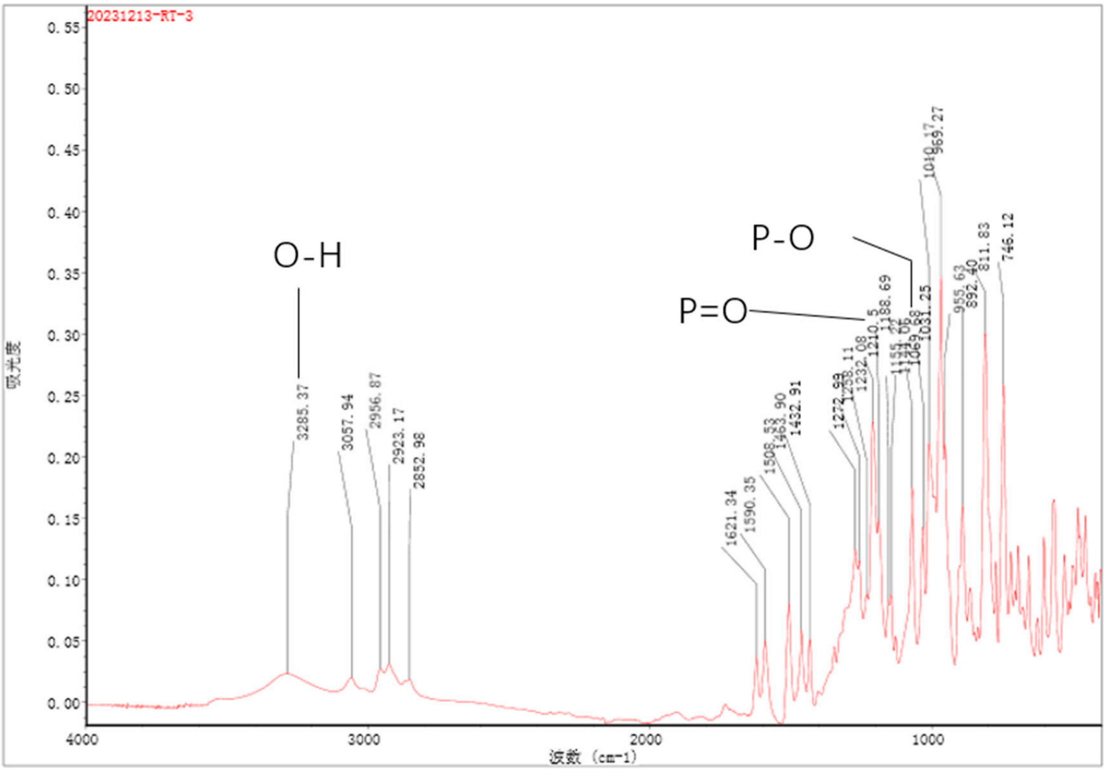

Fig. S6 IR spectrum of **P6**/[Rh] = 2:1

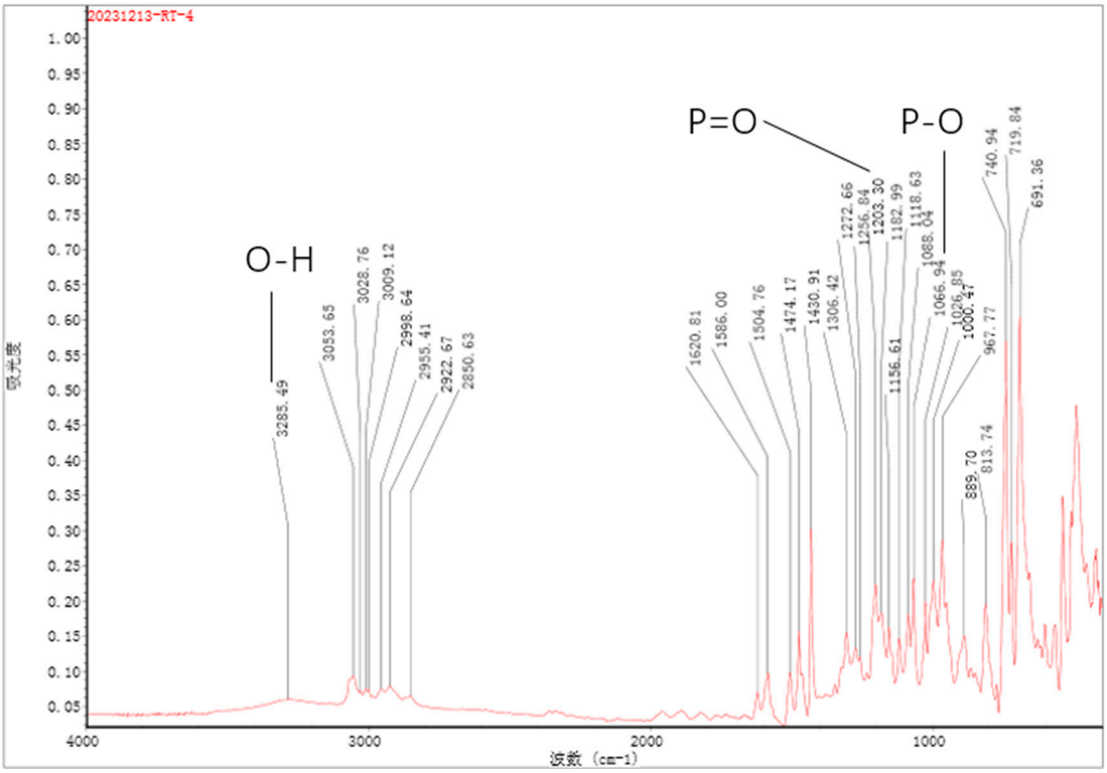

Fig. S7 IR spectrum of **P6**/[Rh]/PPh_3 = 1:1:2

10. NMR Spectra

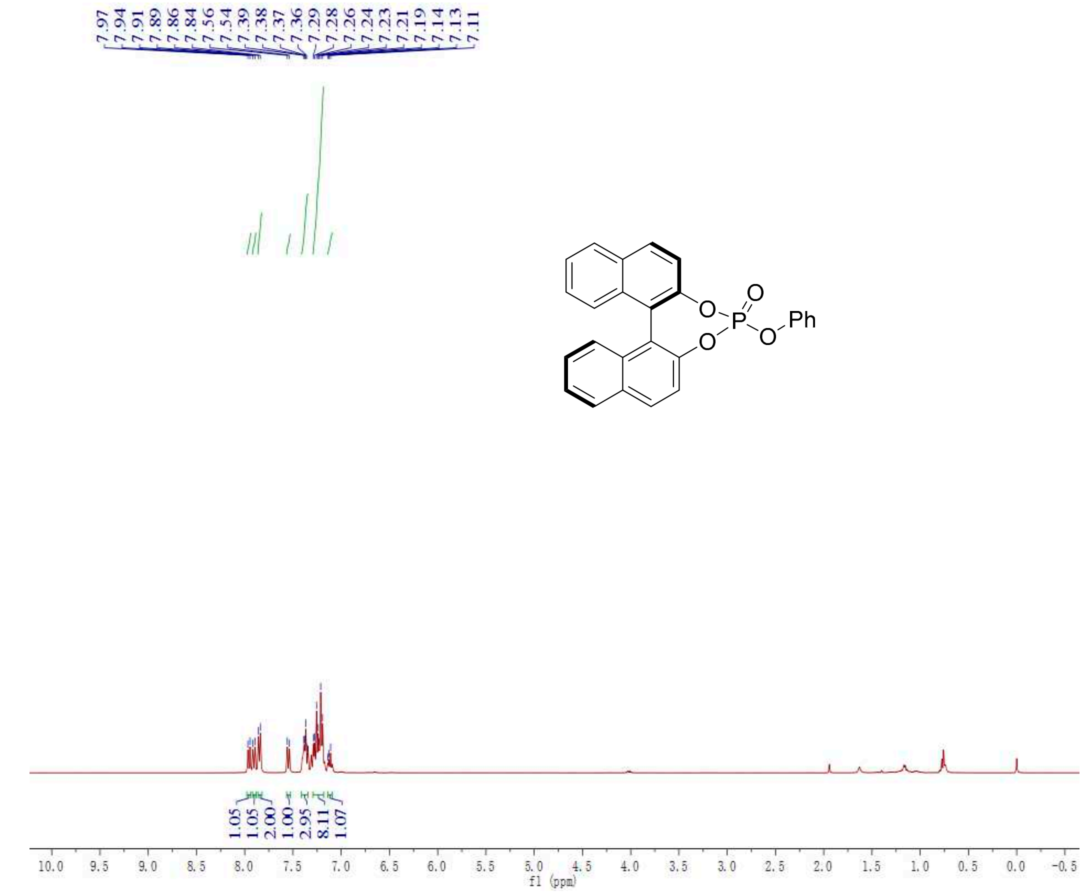

Fig. S8 ^{1}H-NMR spectrum of (*R*)-**P4** (400 MHz, $CDCl_3$)

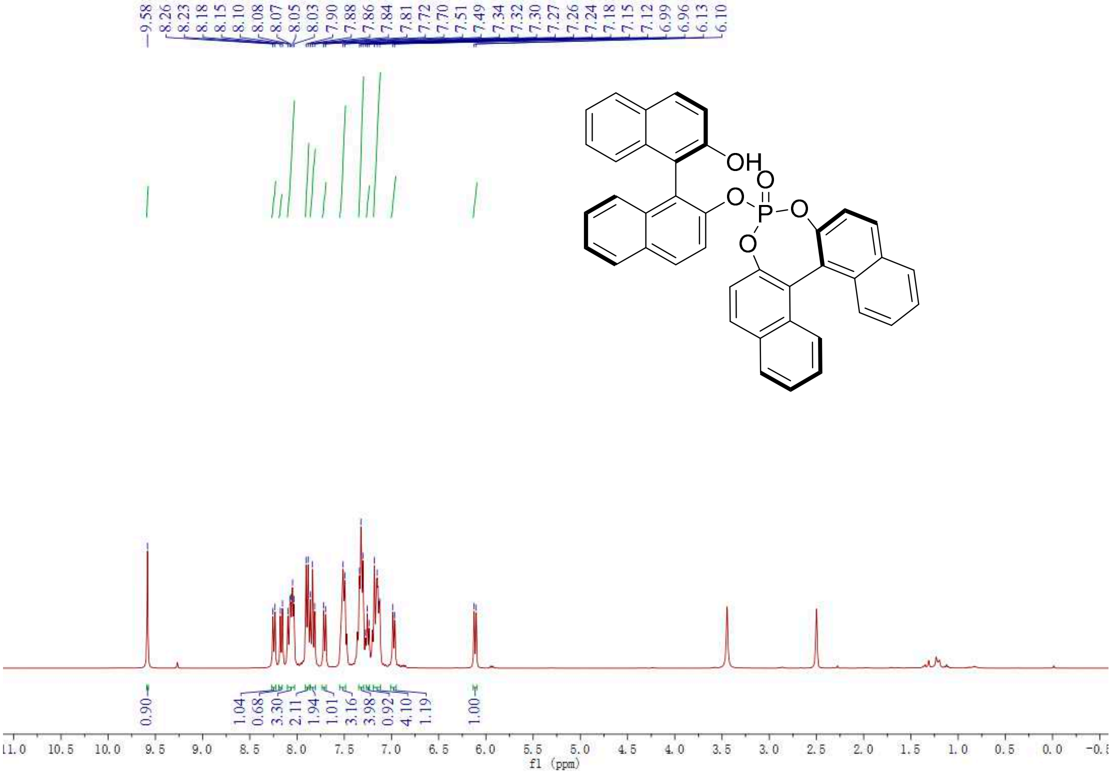

Fig. S9 ^{1}H-NMR spectrum of (*R, R*)-**P5** (400 MHz, DMSO-d_6)

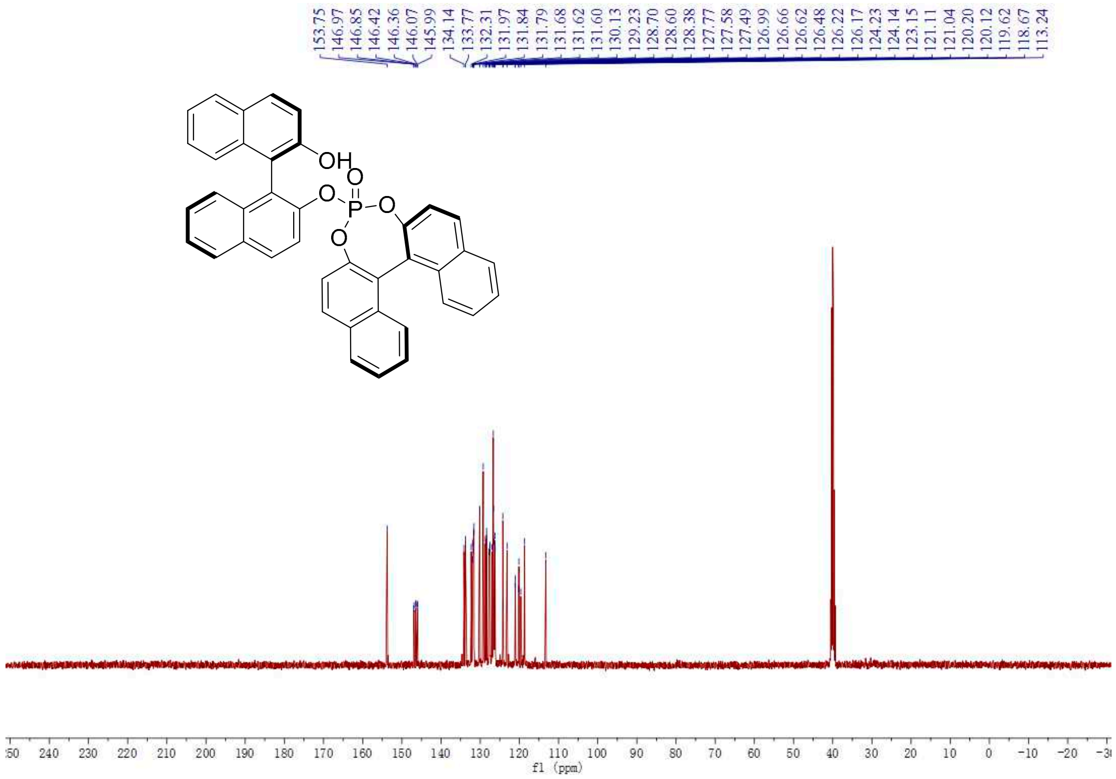

Fig. S10 ^{13}C-NMR spectrum of (*R, R*)-**P5** (100 MHz, DMSO-*d*$_6$)

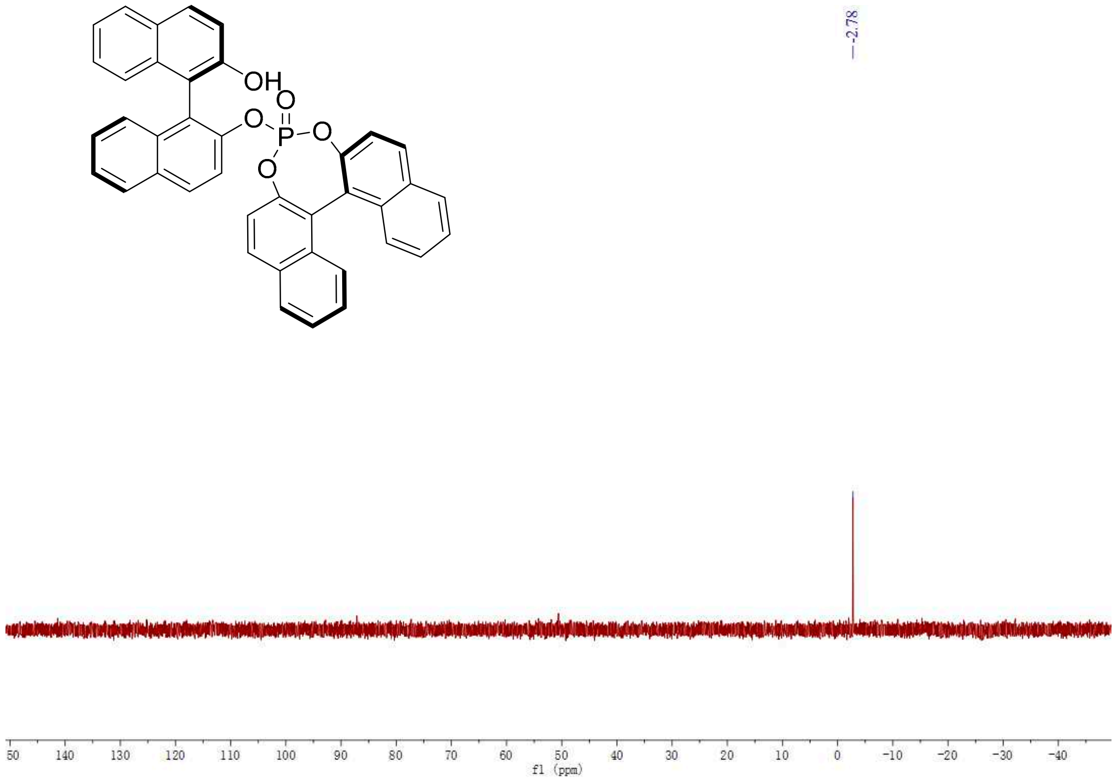

Fig. S11 ^{31}P-NMR spectrum of (*R, R*)-**P5** (161 MHz, DMSO-*d*$_6$)

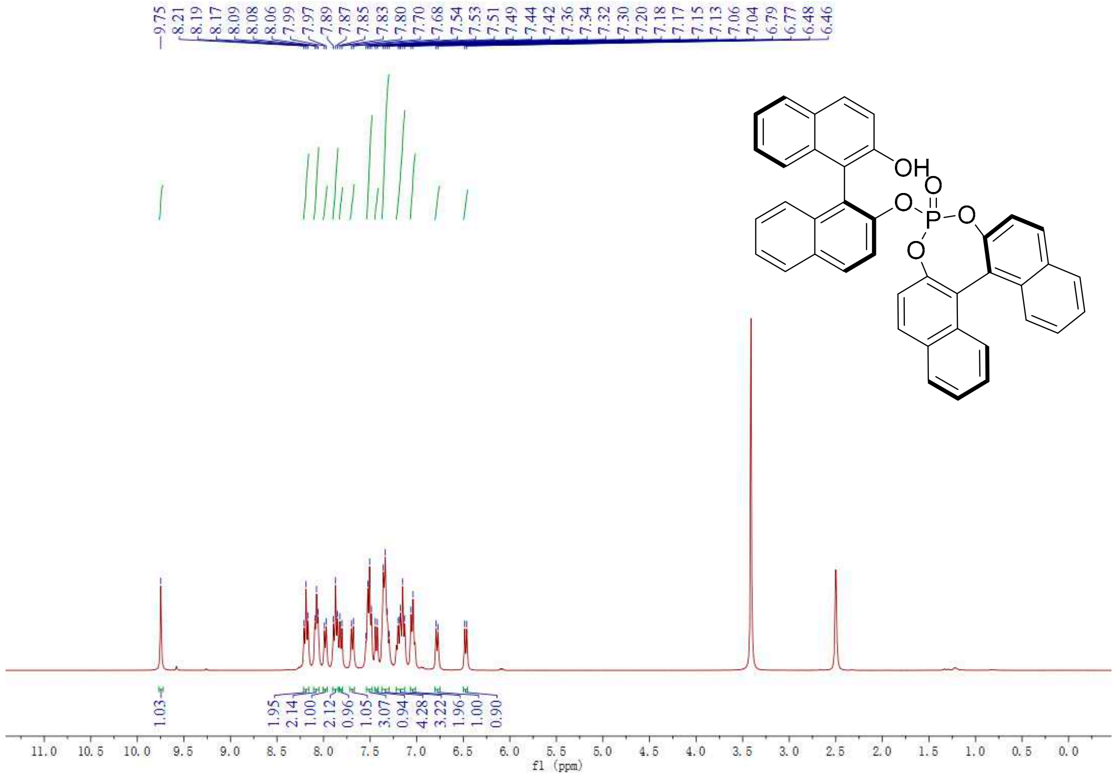

Fig. S12 ^{1}H-NMR spectrum of (*S, R*)**-P6** (400 MHz, DMSO-d_6)

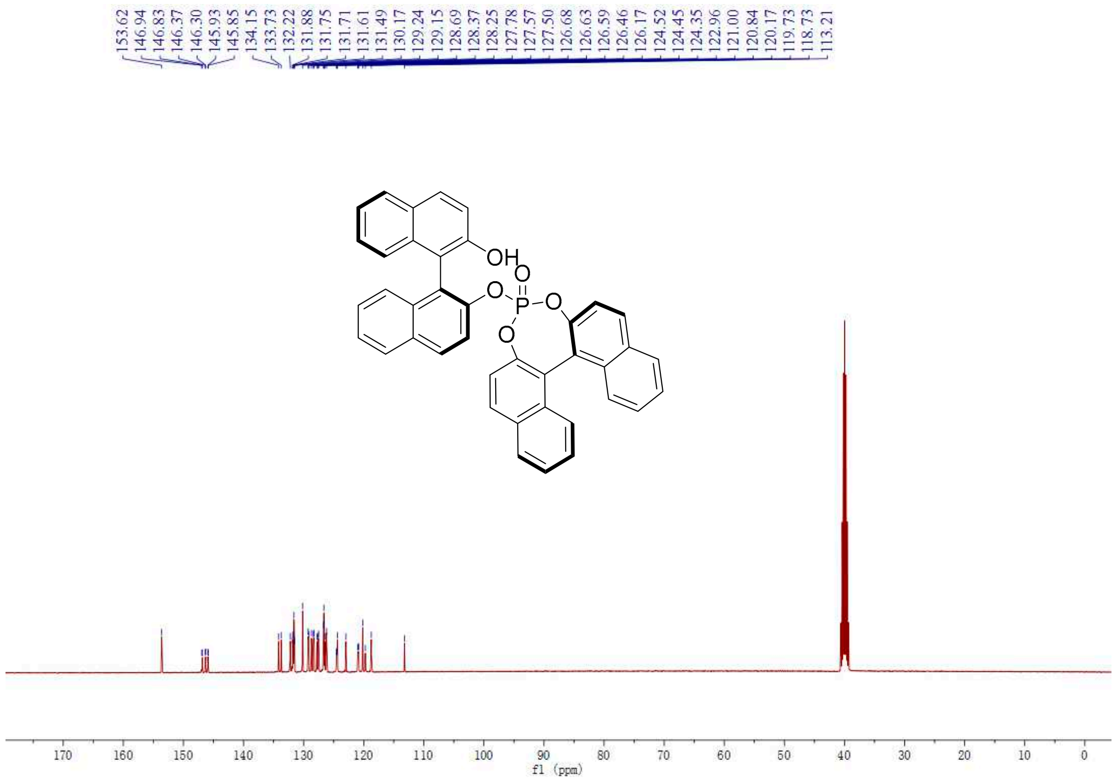

Fig. S13 ^{13}C-NMR spectrum of (*S, R*)**-P6** (100 MHz, DMSO-d_6)

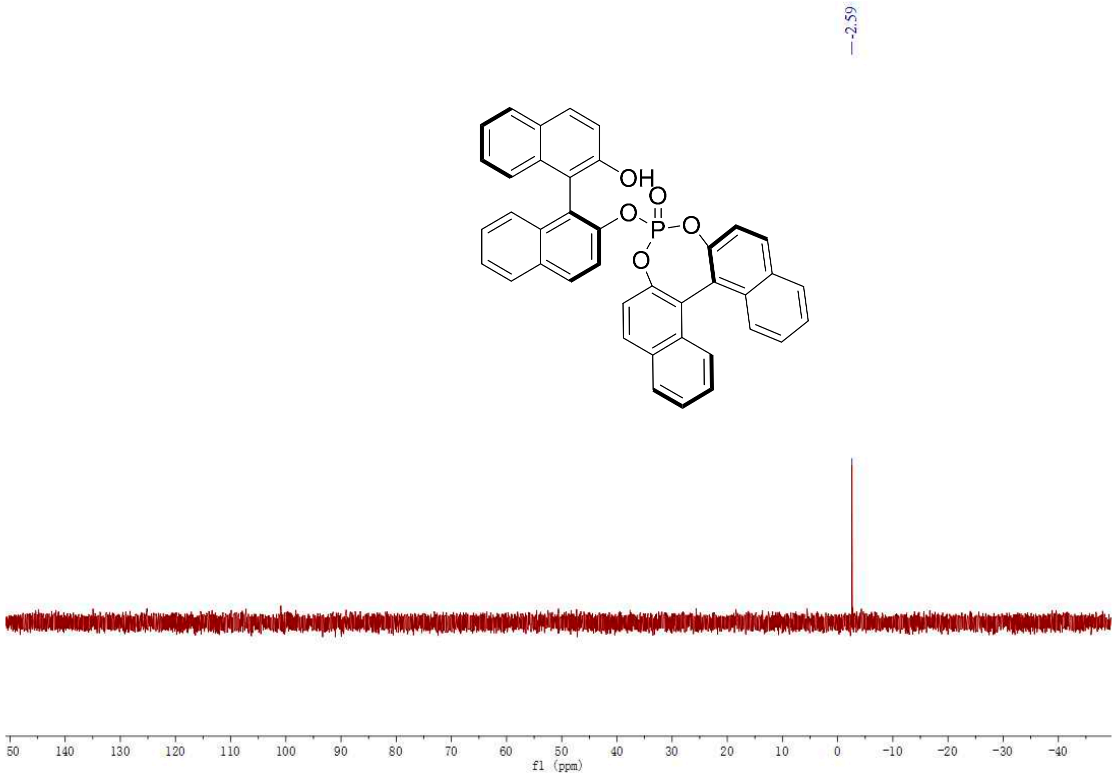

Fig. S14 ^{31}P-NMR spectrum of (*S, R*)**-P6** (161 MHz, DMSO-d_6)

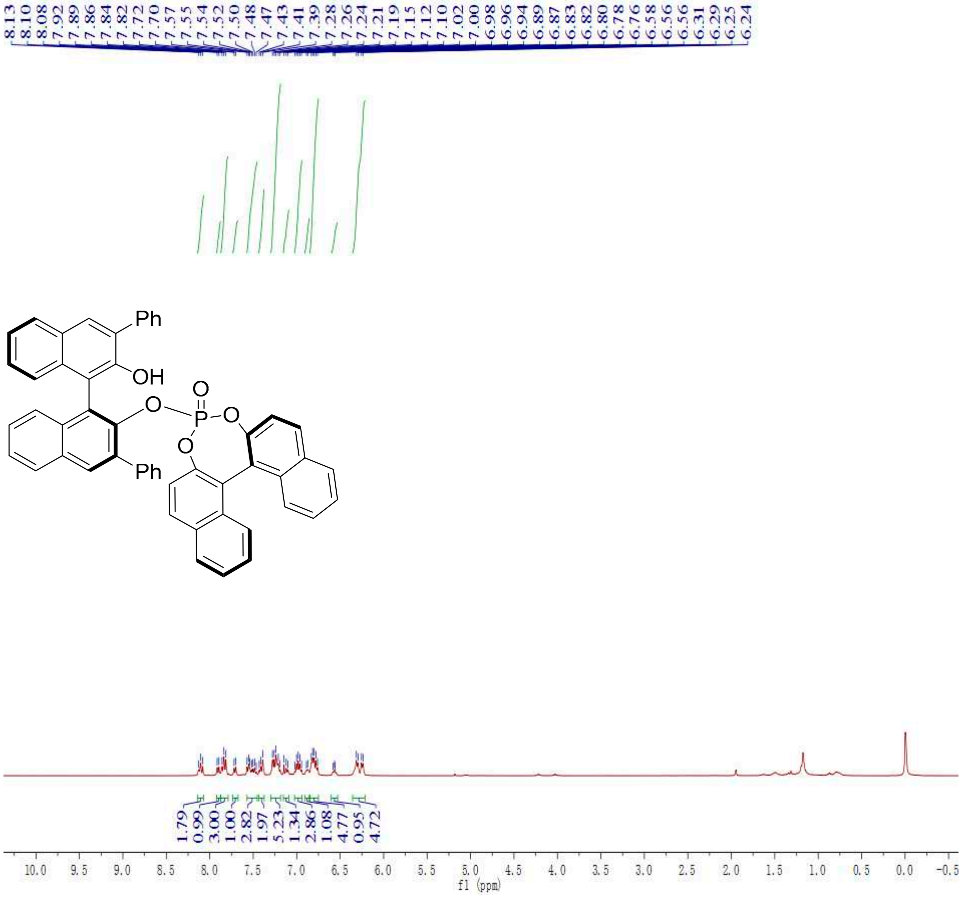

Fig. S15 ^{1}H-NMR spectrum of (*S, R*)**-P7** (400 MHz, $CDCl_3$)

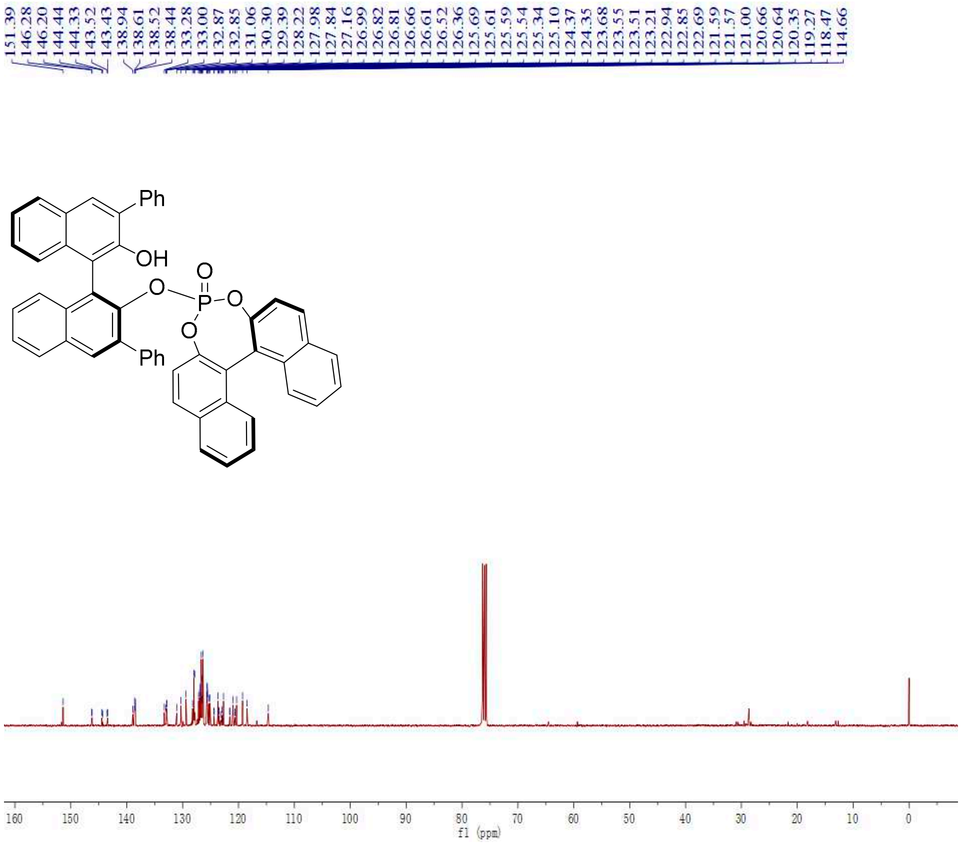

Fig. S16 ^{13}C-NMR spectrum of (*S, R*)-**P7** (100 MHz, CDCl$_3$)

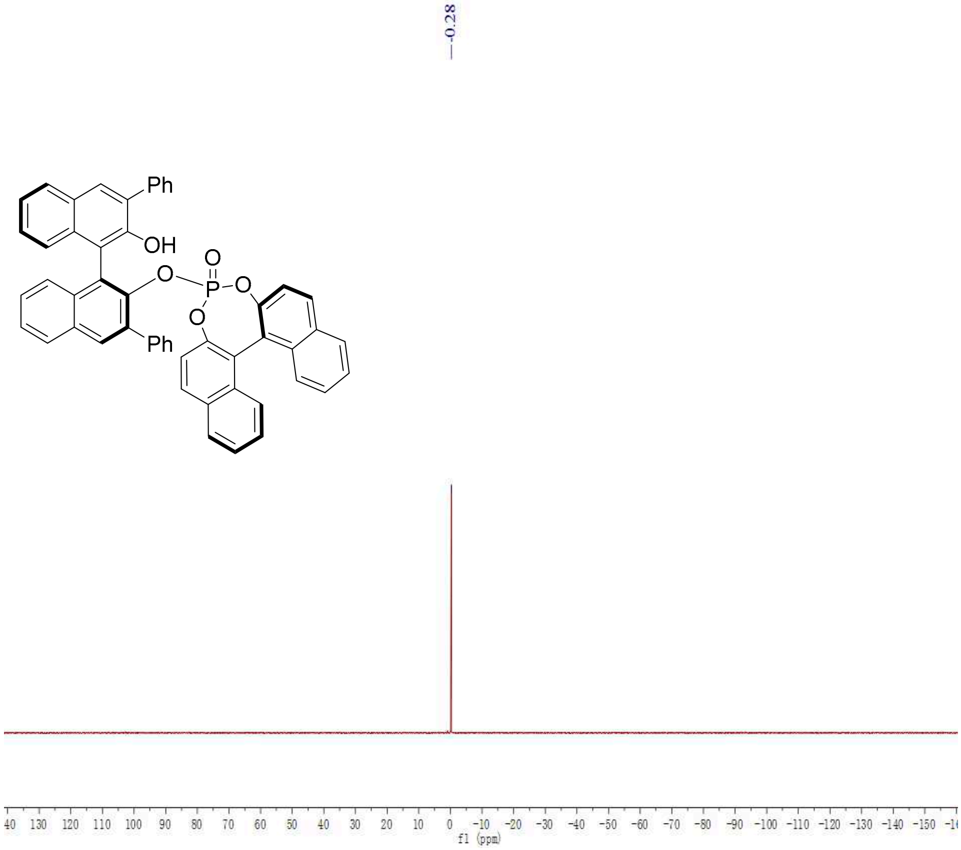

Fig. S17 ^{31}P-NMR spectrum of (*S, R*)-**P7** (161 MHz, CDCl$_3$)

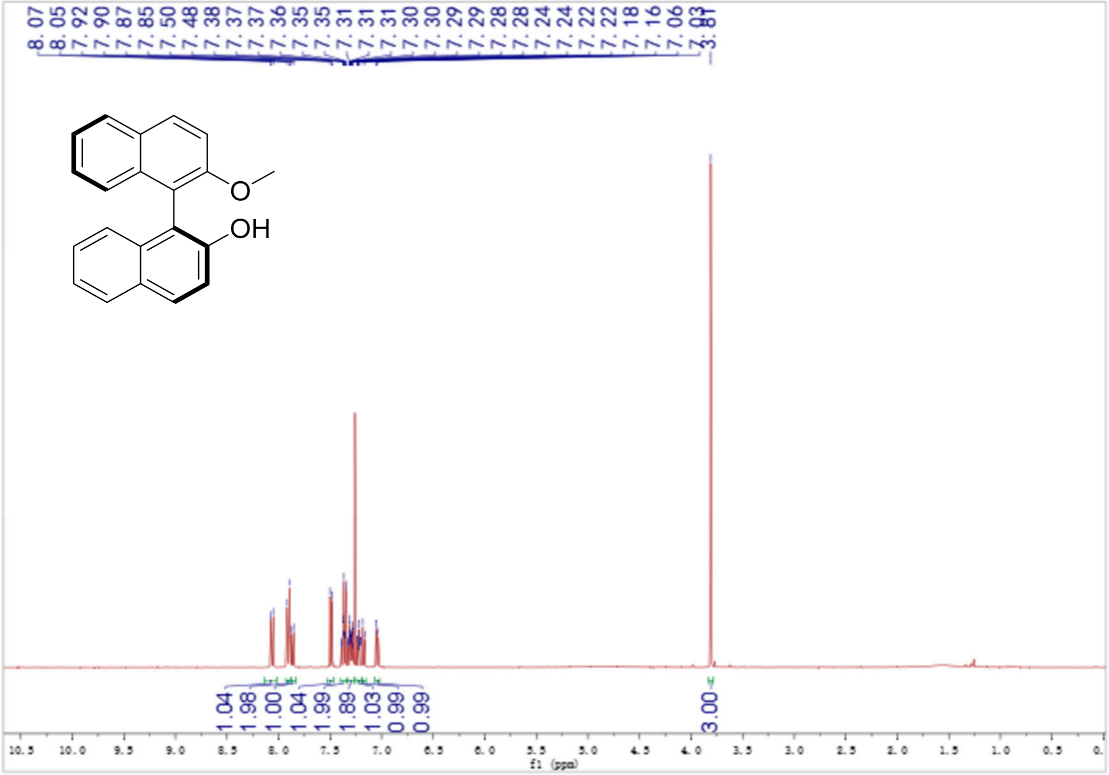

Fig. S18 ^{1}H-NMR spectrum of **S3** (400 MHz, $CDCl_3$)

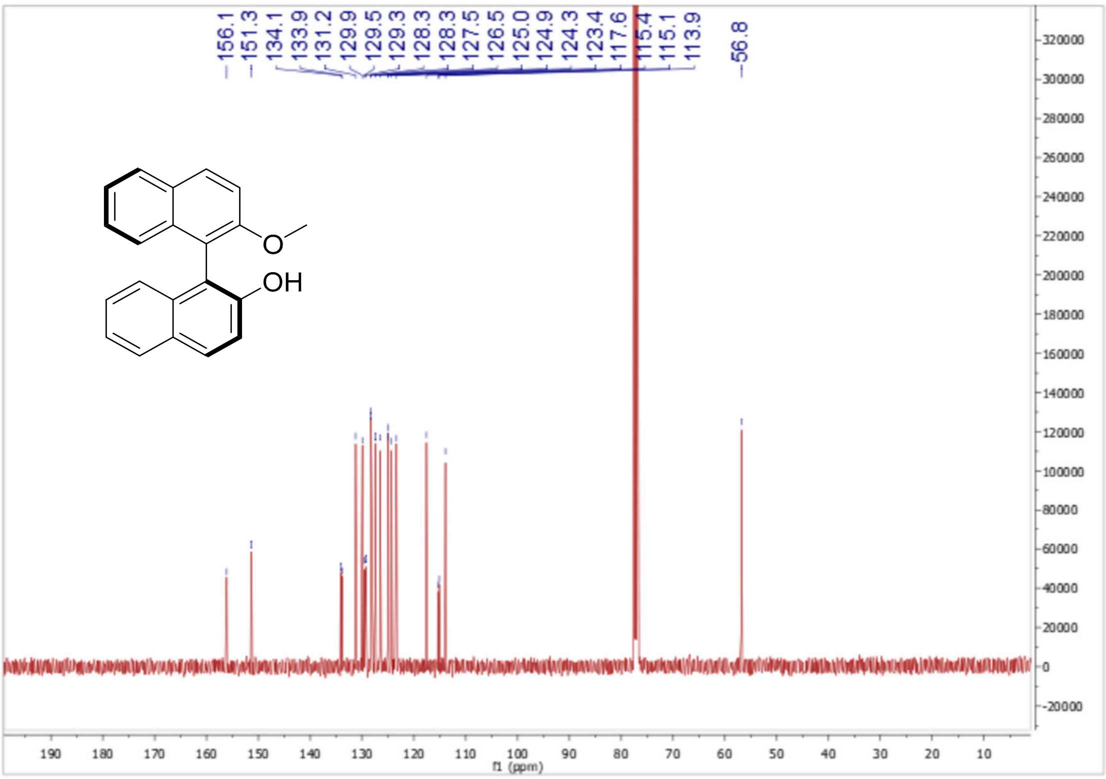

Fig. S19 ^{13}C-NMR spectrum of **S3** (100 MHz, $CDCl_3$)

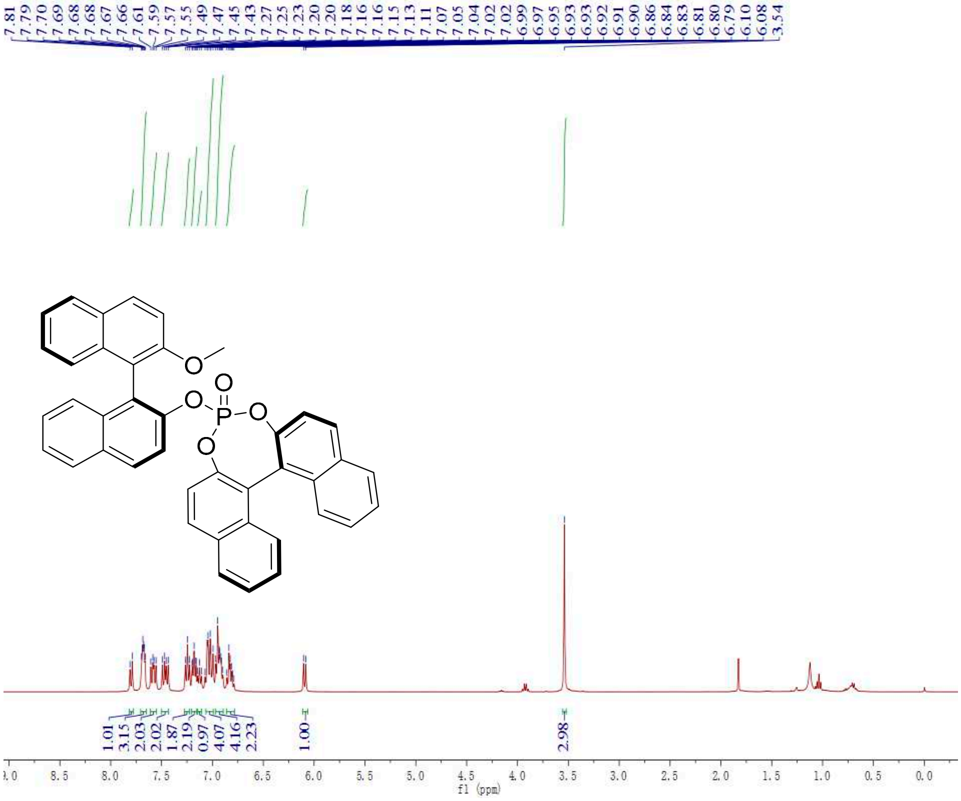

Fig. S20 ^{1}H-NMR spectrum of (*S*, *R*)-**P8** (400 MHz, $CDCl_3$)

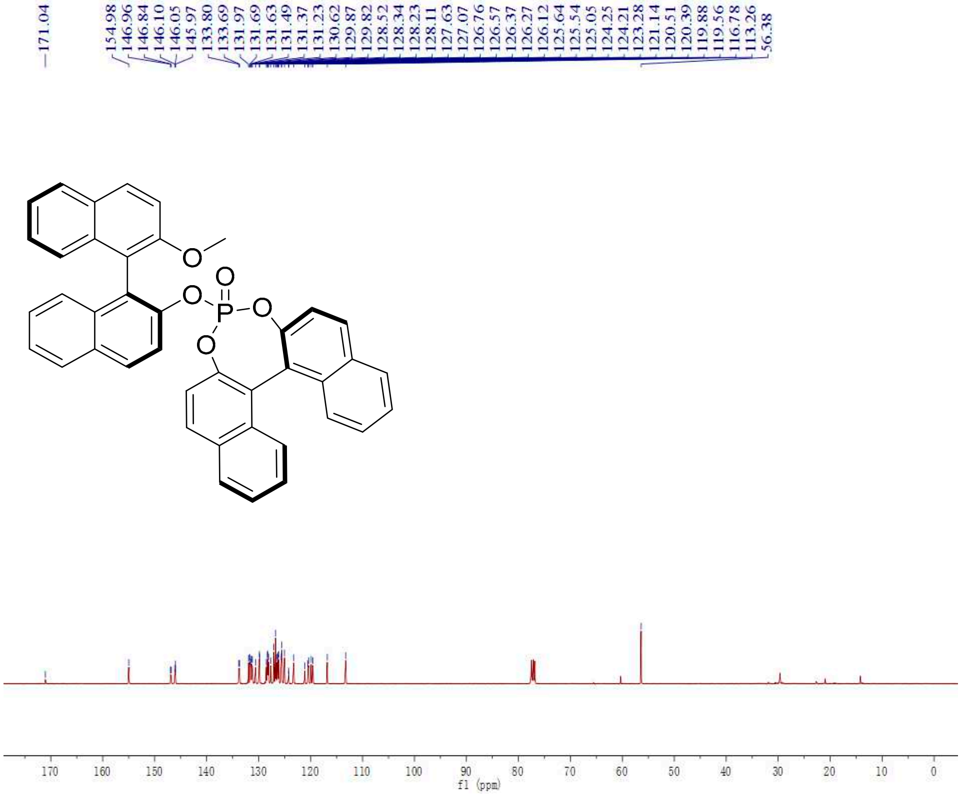

Fig. S21 ^{13}C-NMR spectrum of (*S*, *R*)-**P8** (100 MHz, $CDCl_3$)

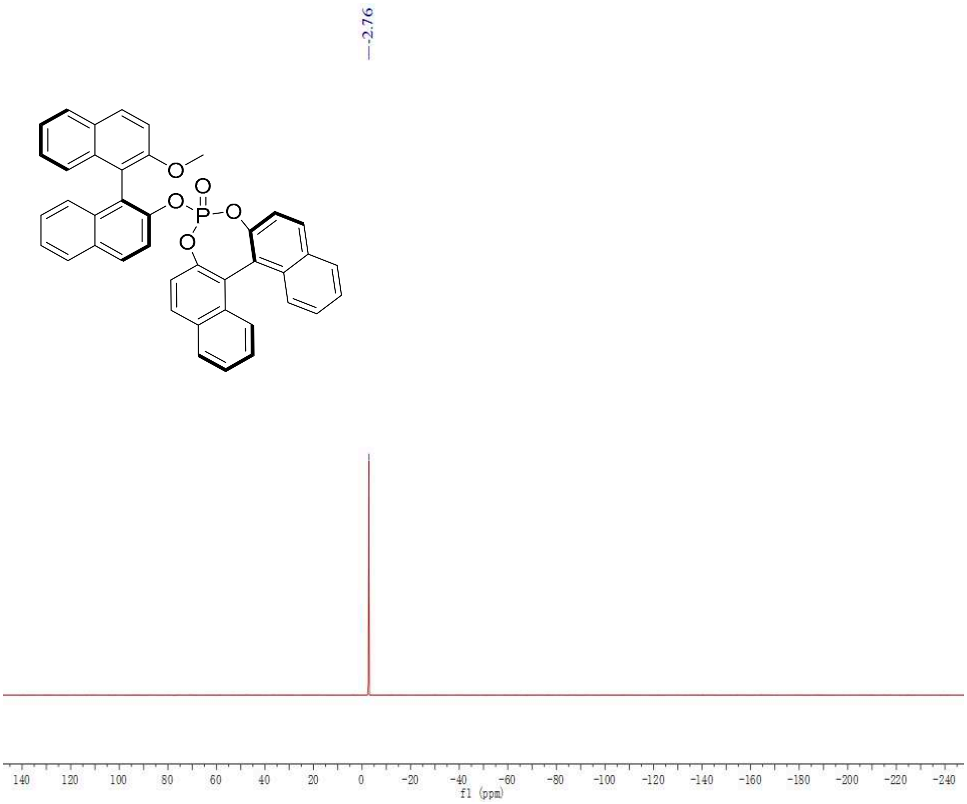

Fig. S22 ^{31}P-NMR spectrum of (*S*, *R*)-**P8** (400 MHz, CDCl$_3$)

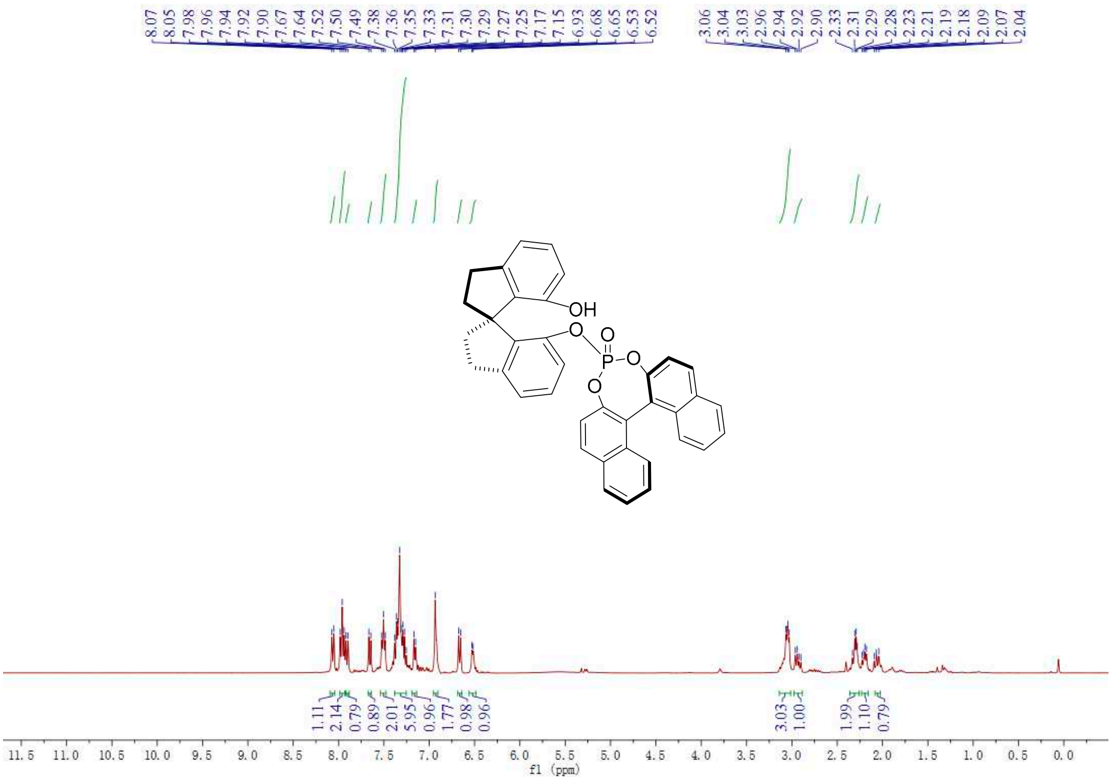

Fig. S23 ^{1}H-NMR spectrum of (*R*, *R*)-**P9** (400 MHz, CDCl$_3$)

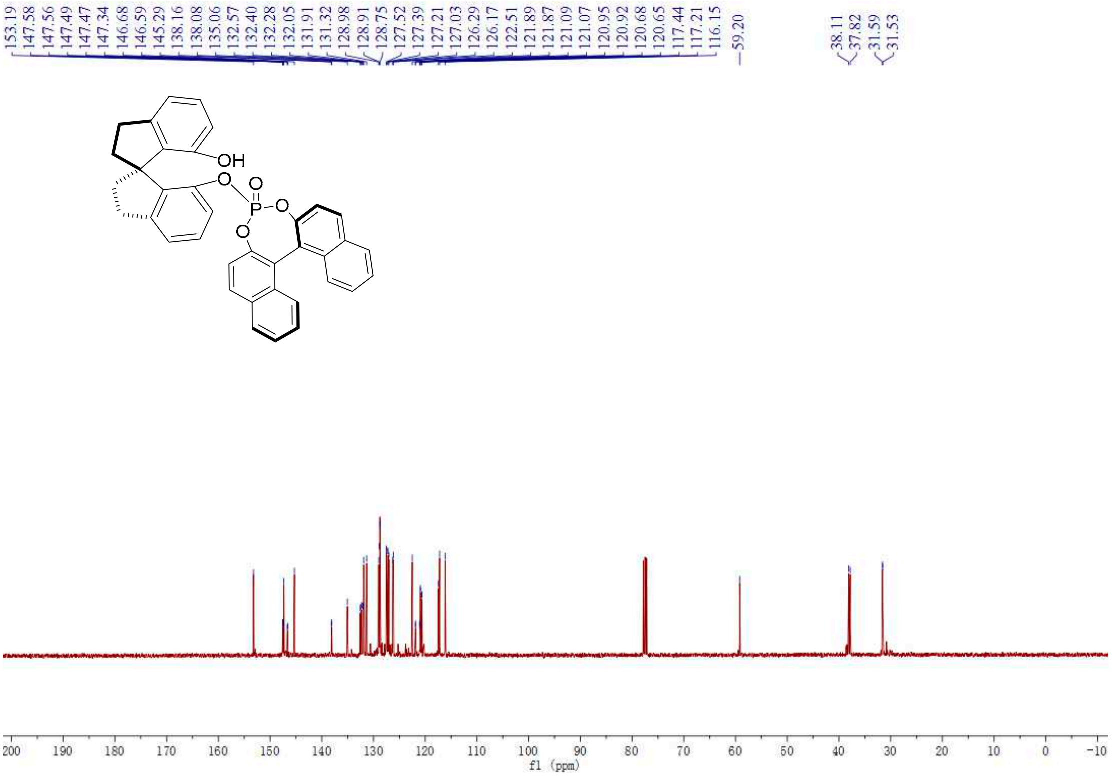

Fig. S24 ^{13}C-NMR spectrum of (*R*, *R*)-**P9** (100 MHz, $CDCl_3$)

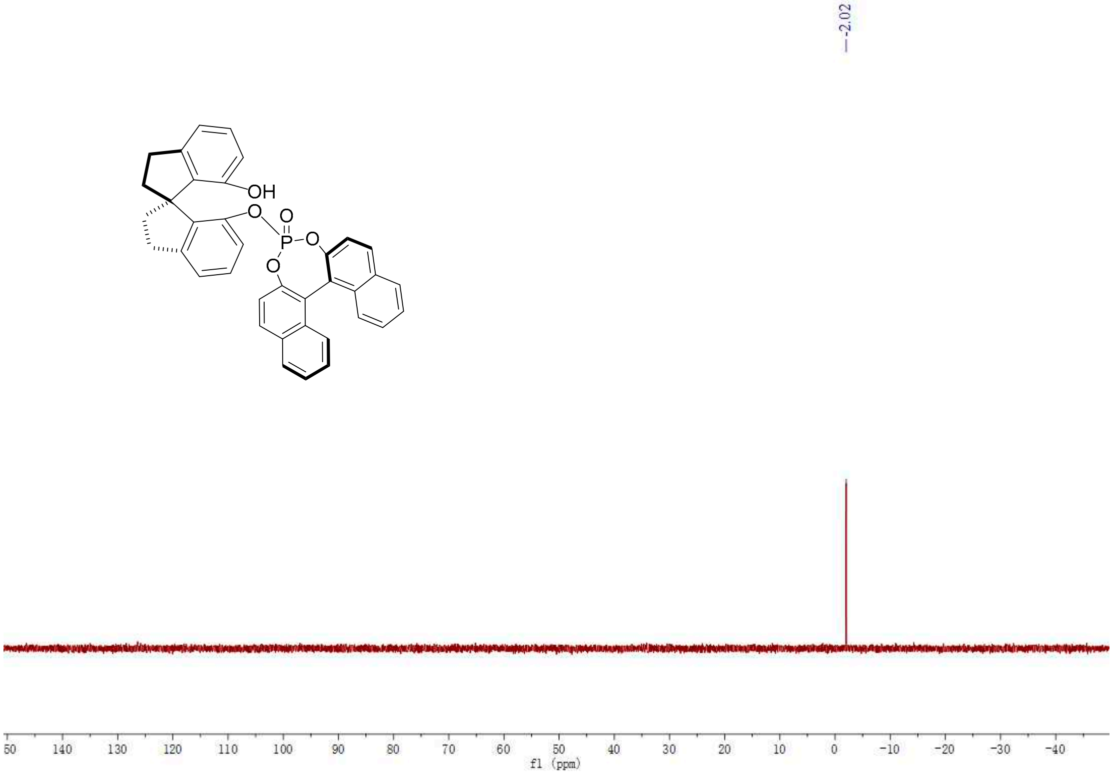

Fig. S25 ^{31}P-NMR spectrum of (*R*, *R*)-**P9** (161 MHz, $CDCl_3$)

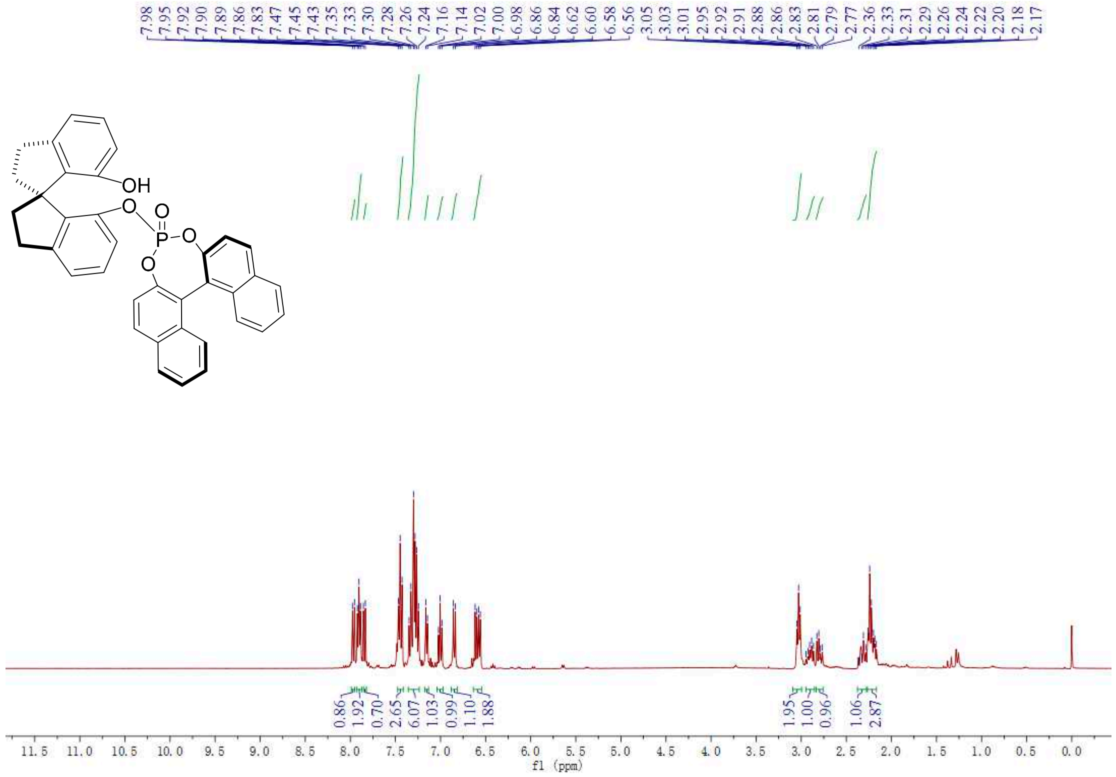

Fig. S26 ^{1}H-NMR spectrum of (*S*, *R*)-**P10** (400 MHz, $CDCl_3$)

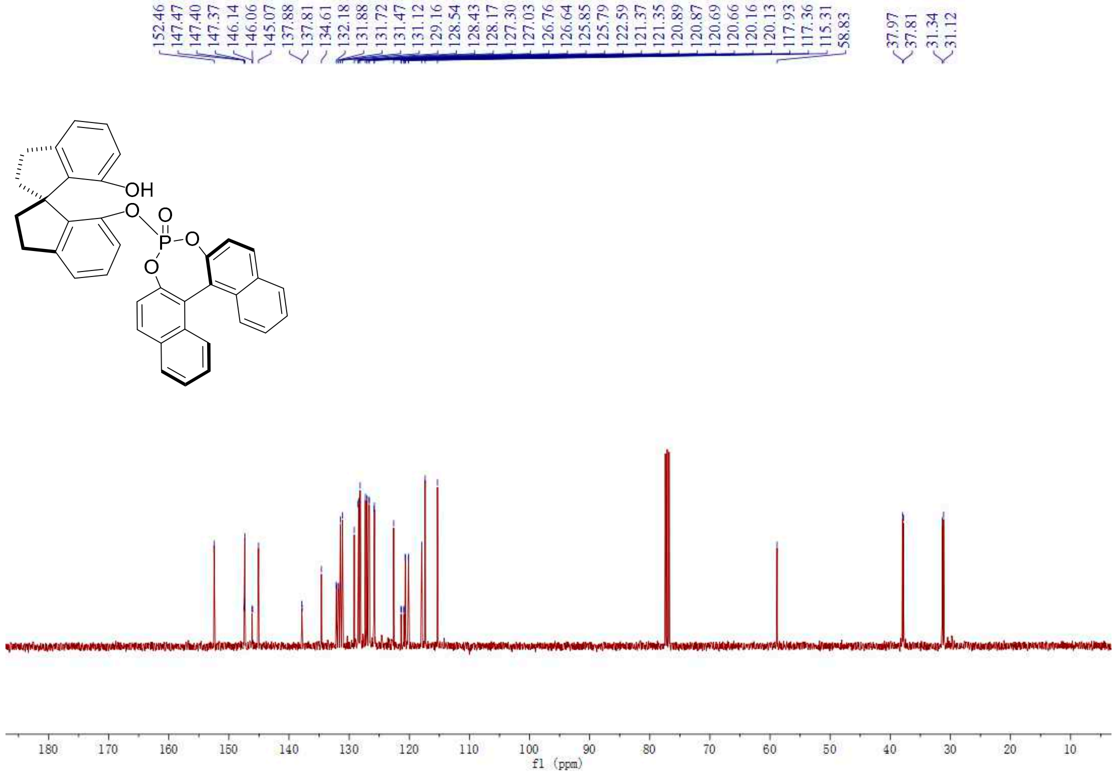

Fig. S27 ^{13}C-NMR spectrum of (*S*, *R*)-**P10** (161 MHz, $CDCl_3$)

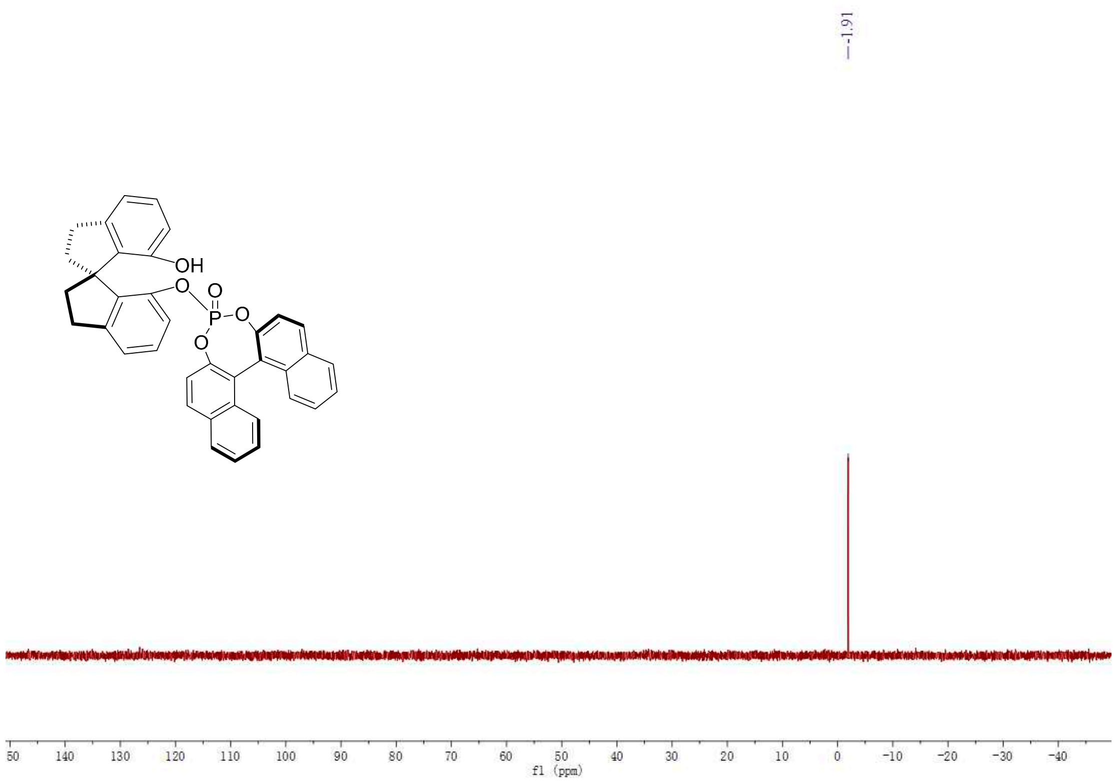

Fig. S28 ^{31}P NMR spectrum of (*S*, *R*)-**P10** (161 MHz, CDCl$_3$)

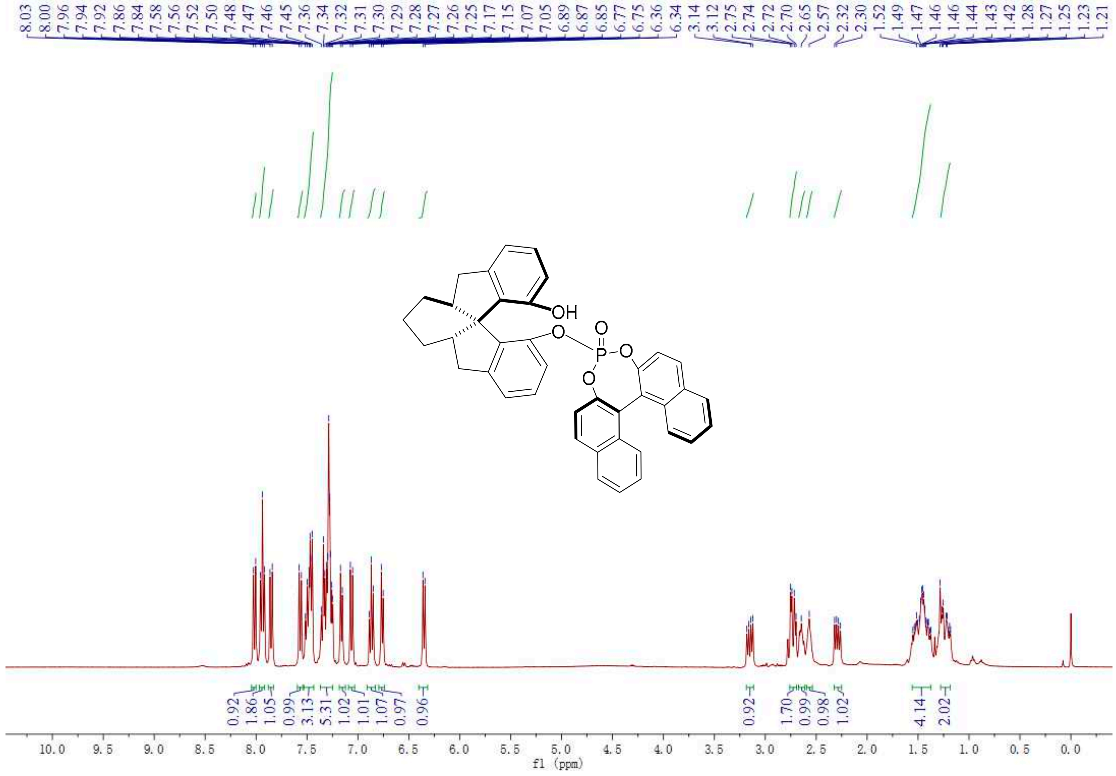

Fig. S29 ^{1}H NMR spectrum of (*S, S, S, S*)-**P11** (400 MHz, CDCl$_3$)

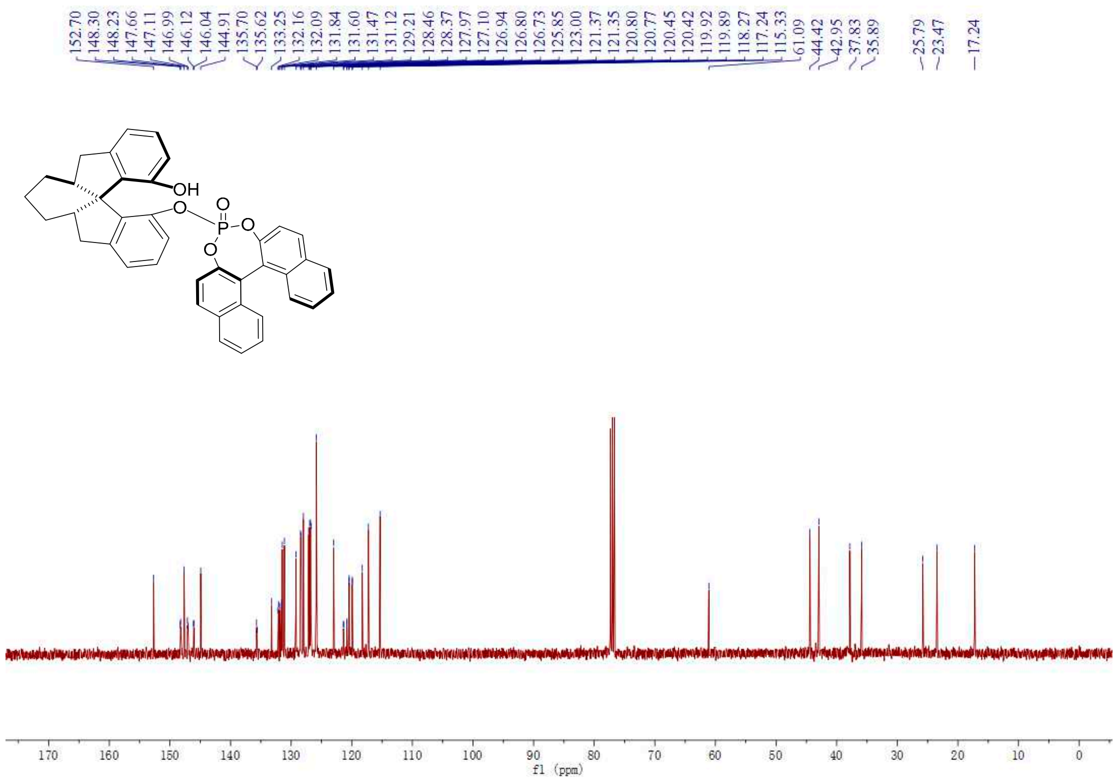

Fig. S30 ^{13}C NMR spectrum of (*S, S, S, S*)-**P11** (100 MHz, $CDCl_3$)

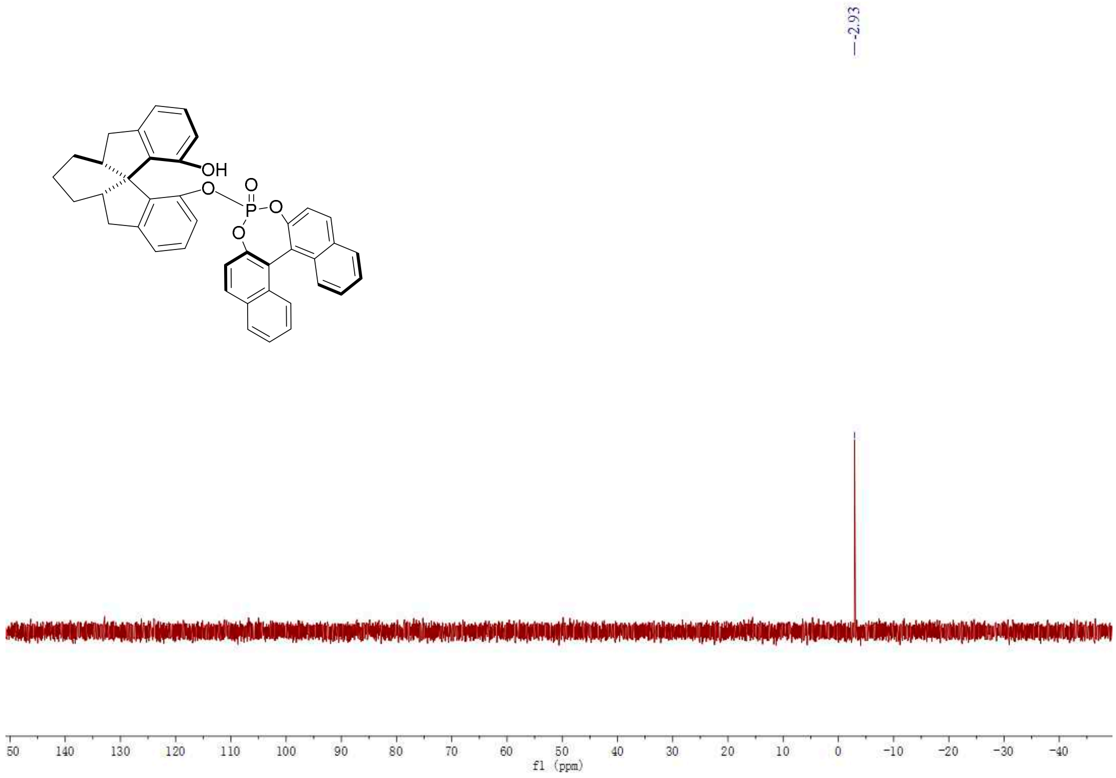

Fig. S31 ^{31}P NMR spectrum of (*S, S, S, S*)-**P11** (400 MHz, $CDCl_3$)

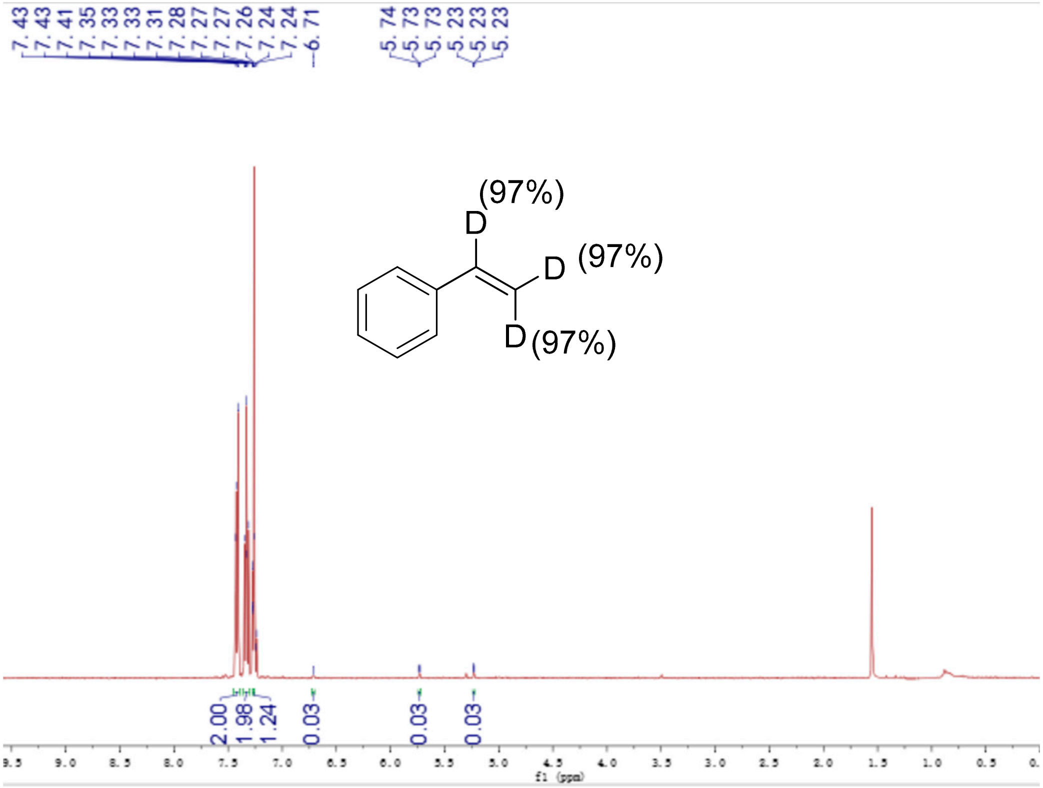

Fig. S32 ^{1}H NMR spectrum of **1a$^{\mathbf{D}}$** (400 MHz, $CDCl_3$)

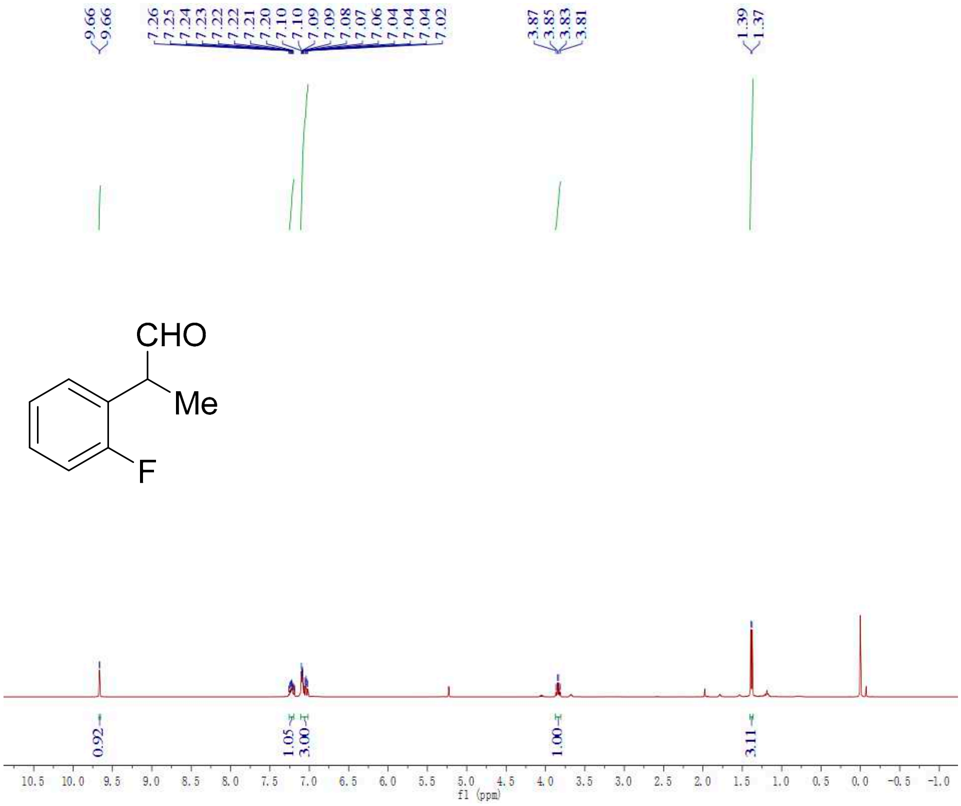

Fig. S33 ^{1}H NMR spectrum of **2b** (400 MHz, $CDCl_3$)

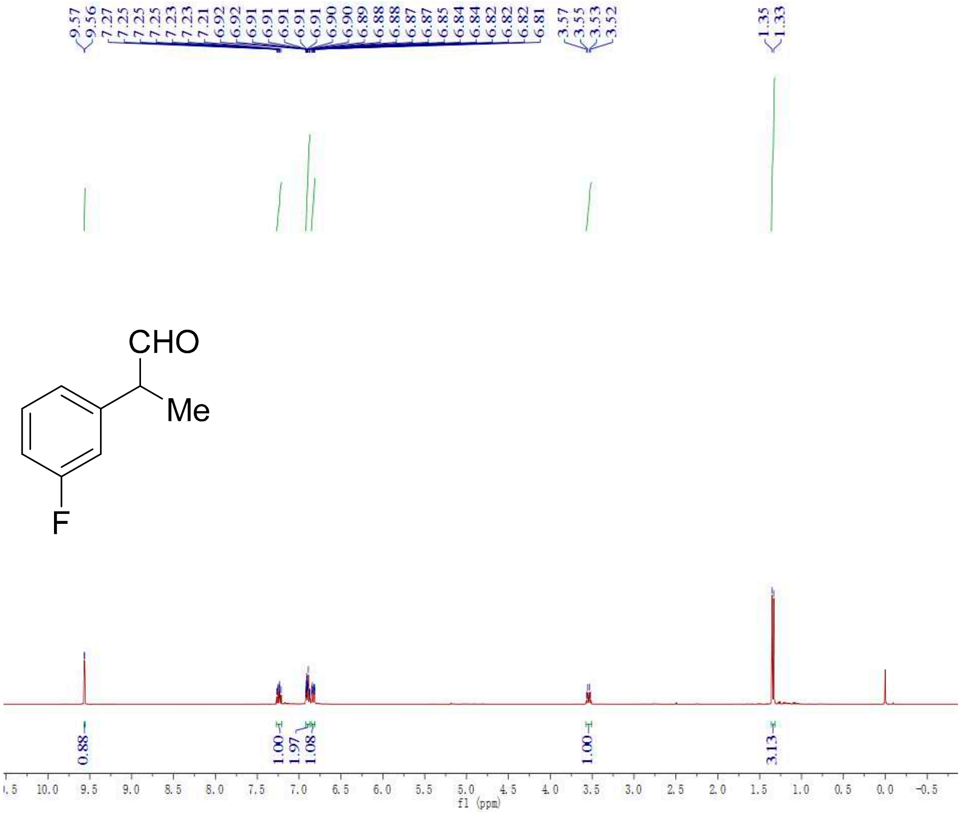

Fig. S34 ^{1}H NMR spectrum of **2c** (400 MHz, $CDCl_3$)

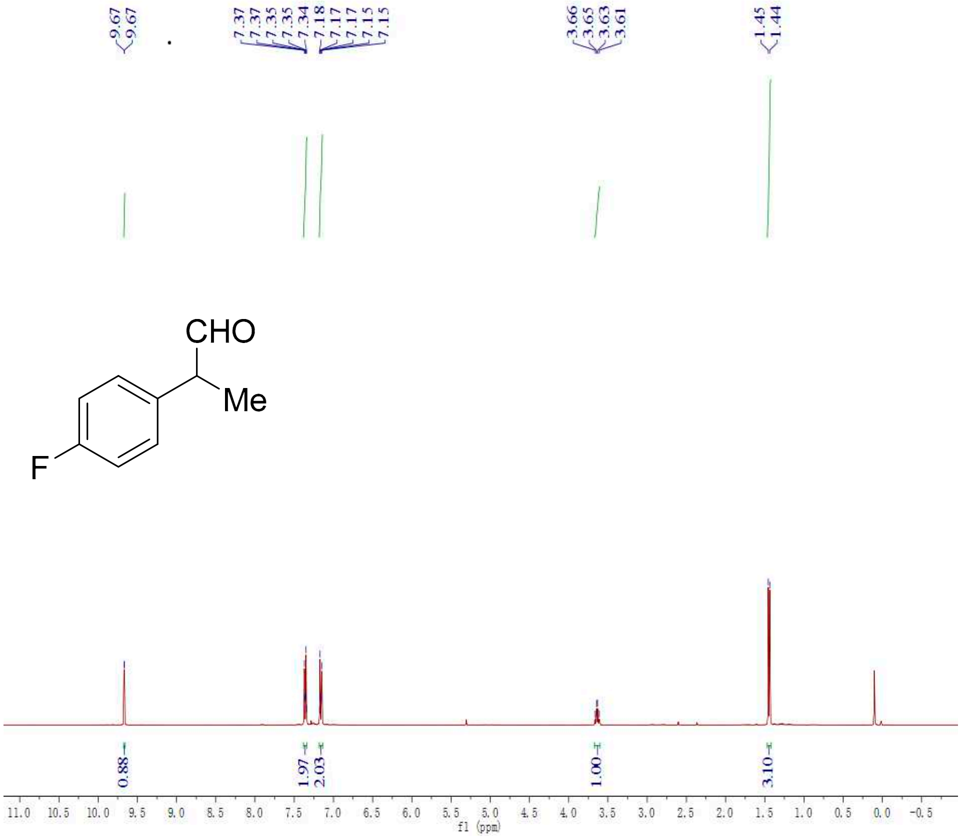

Fig. S35 ^{1}H NMR spectrum of **2d** (400 MHz, $CDCl_3$)

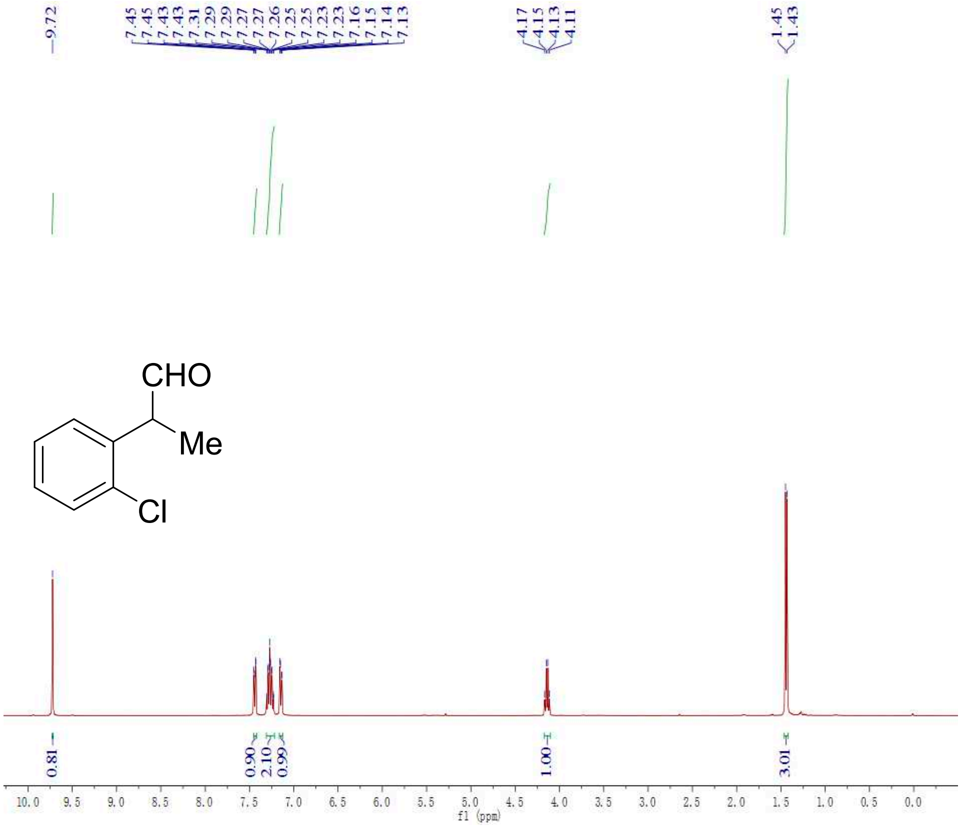

Fig. S36 ^{1}H NMR spectrum of **2e** (400 MHz, $CDCl_3$)

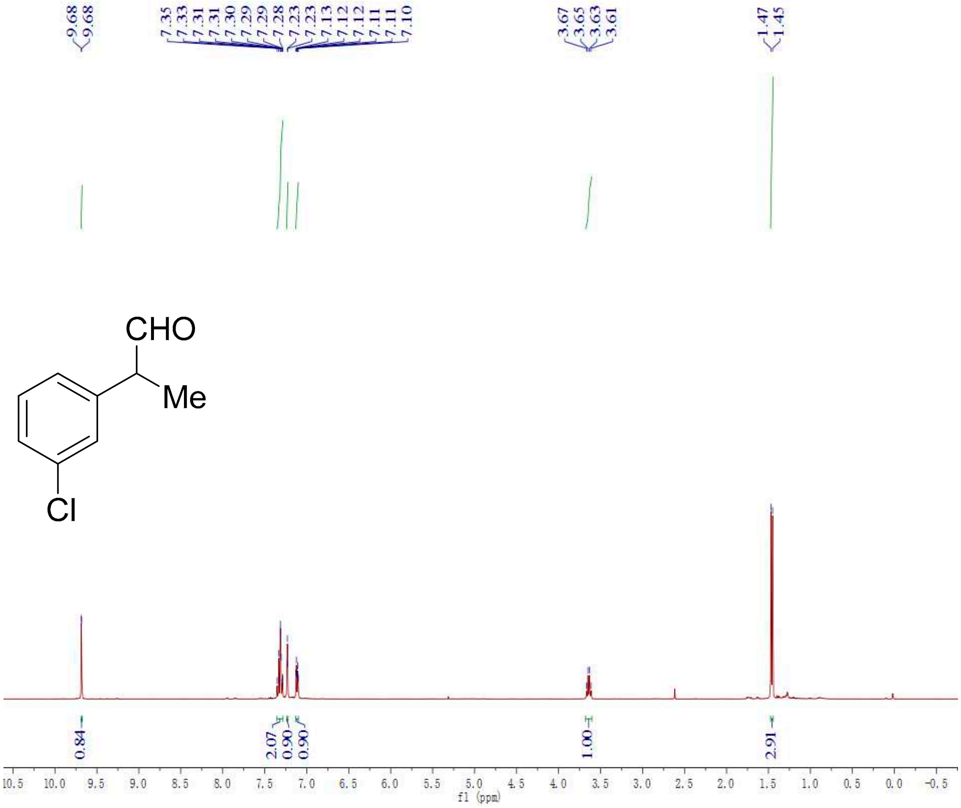

Fig. S37 ^{1}H NMR spectrum of **2f** (400 MHz, $CDCl_3$)

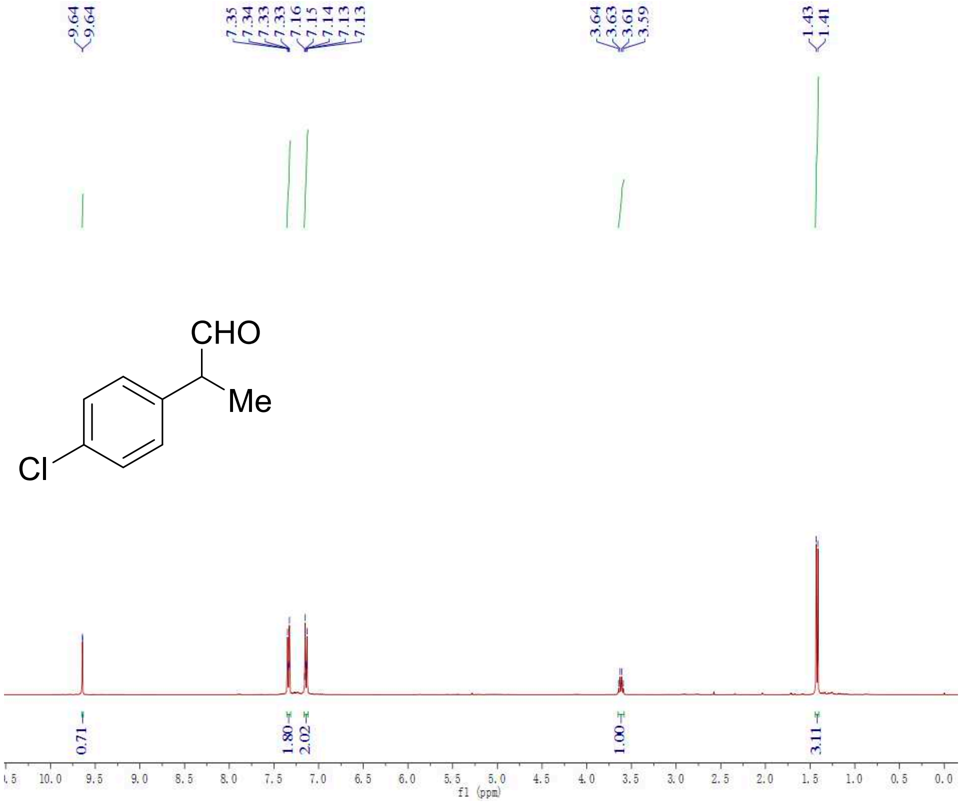

Fig. S38 ^{1}H NMR spectrum of **2g** (400 MHz, $CDCl_3$)

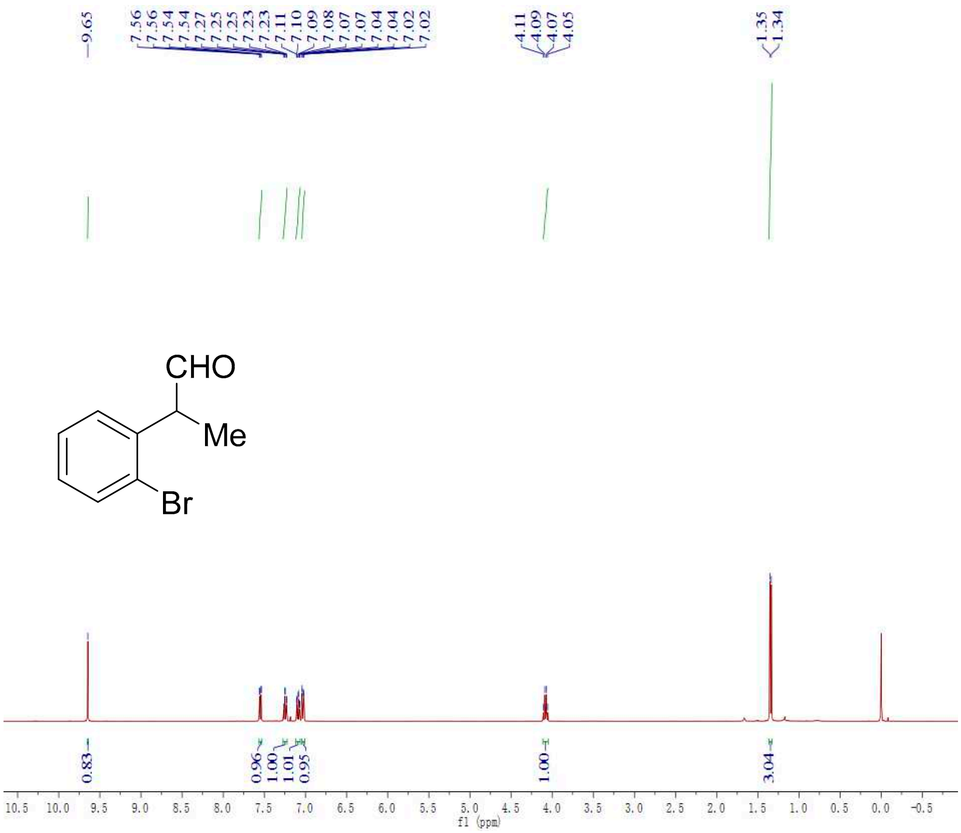

Fig. S39 ^{1}H NMR spectrum of **2h** (400 MHz, $CDCl_3$)

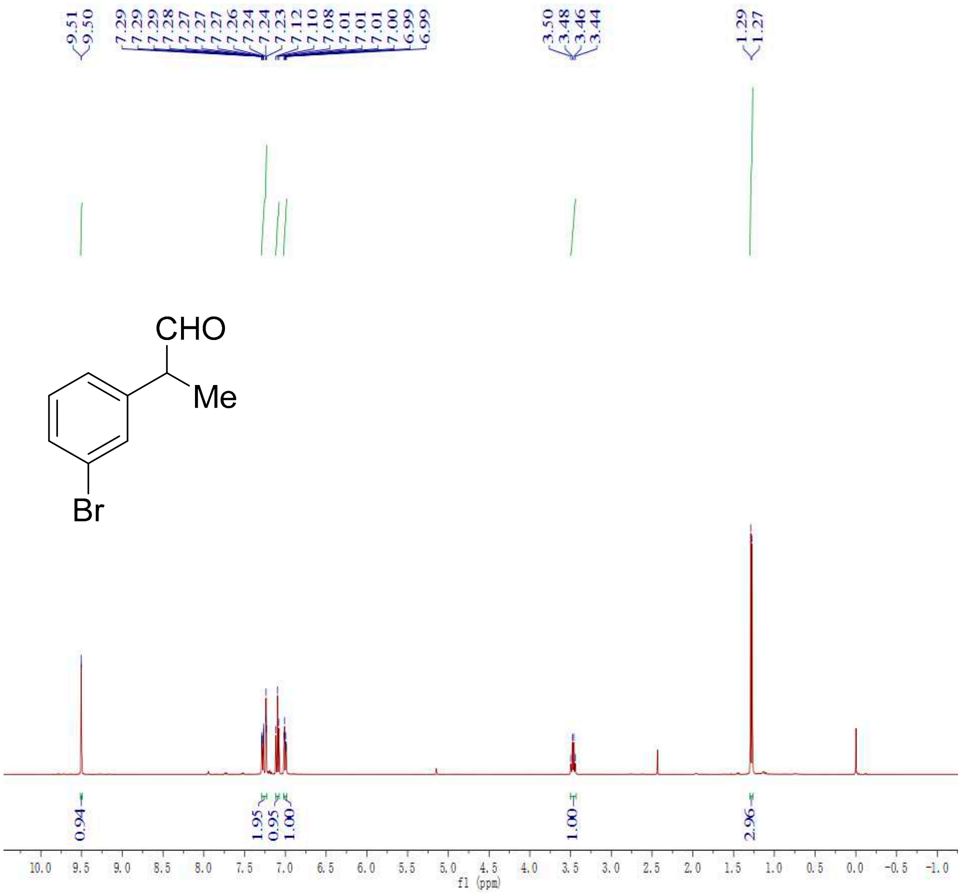

Fig. S40 ^{1}H NMR spectrum of **2i** (400 MHz, $CDCl_3$)

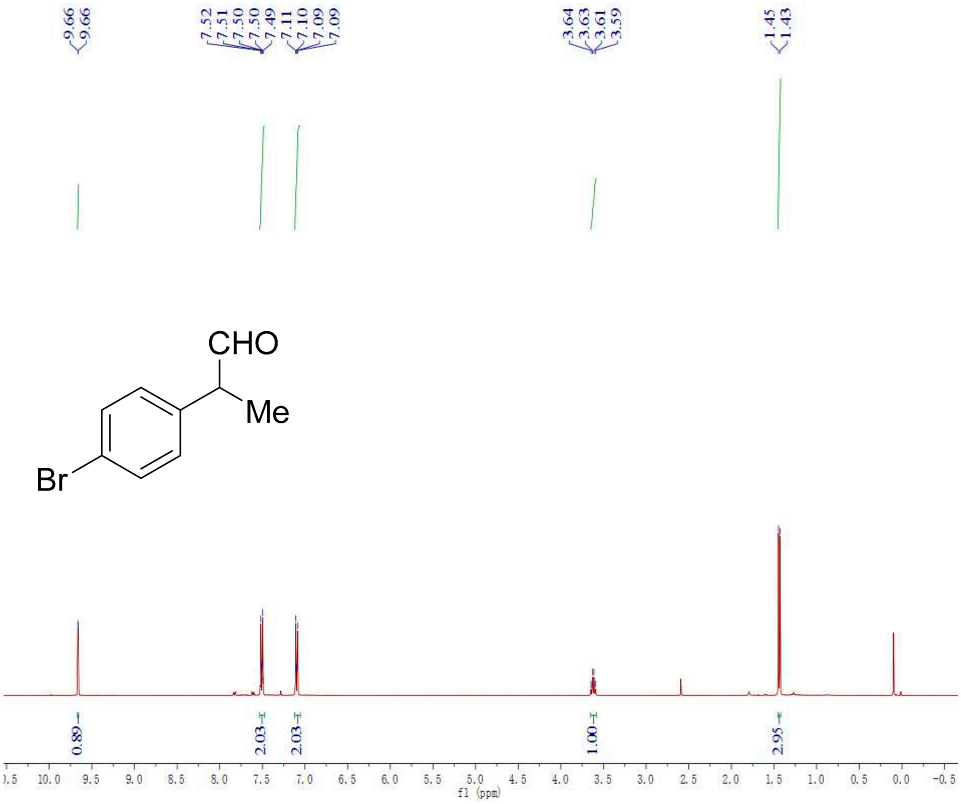

Fig. S41 ^{1}H NMR spectrum of **2j** (400 MHz, $CDCl_3$)

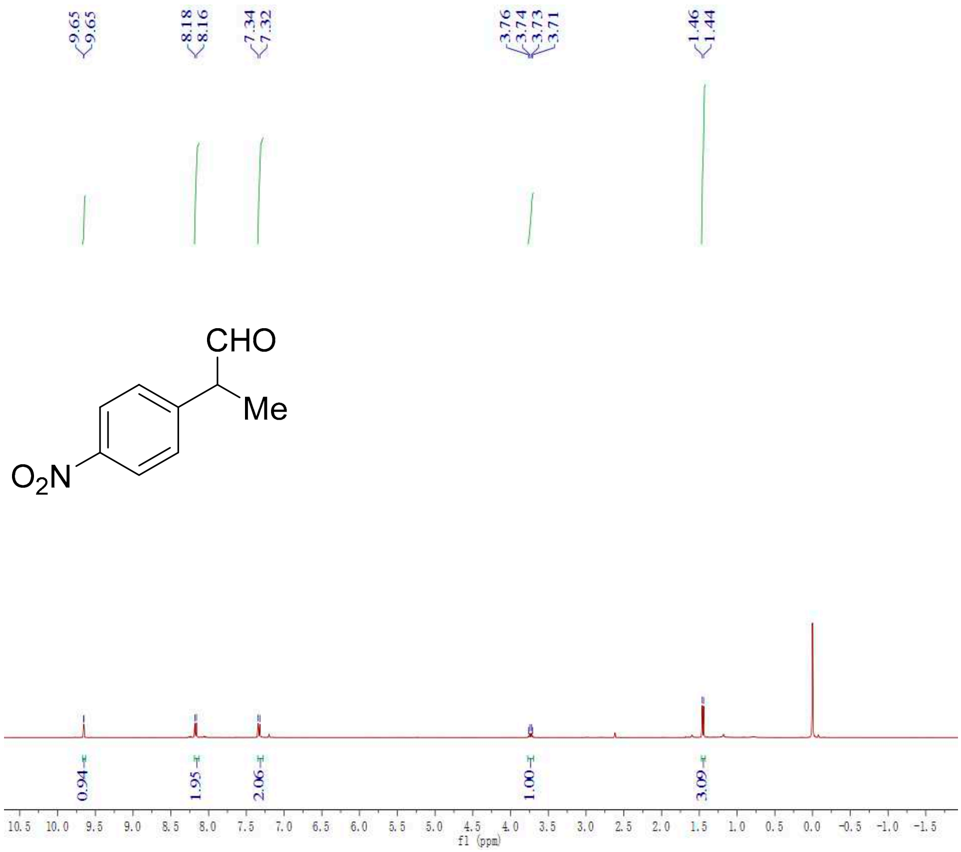

Fig. S42 ^{1}H NMR spectrum of **2k** (400 MHz, $CDCl_3$)

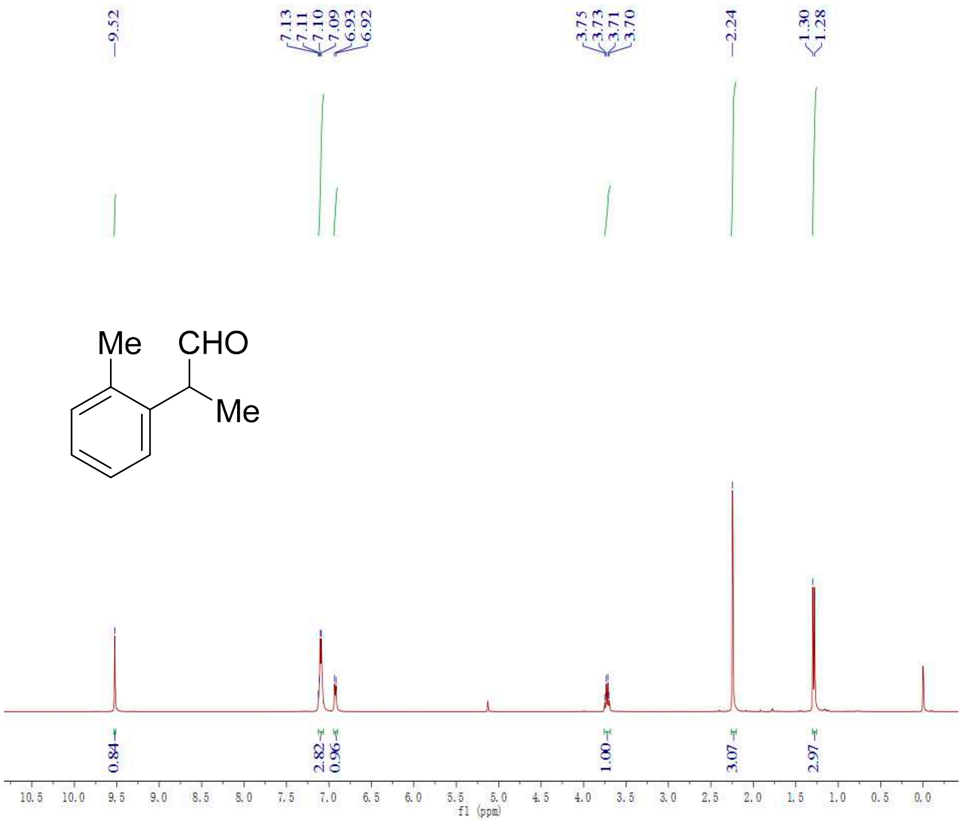

Fig. S43 ^{1}H NMR spectrum of **2l** (400 MHz, $CDCl_3$)

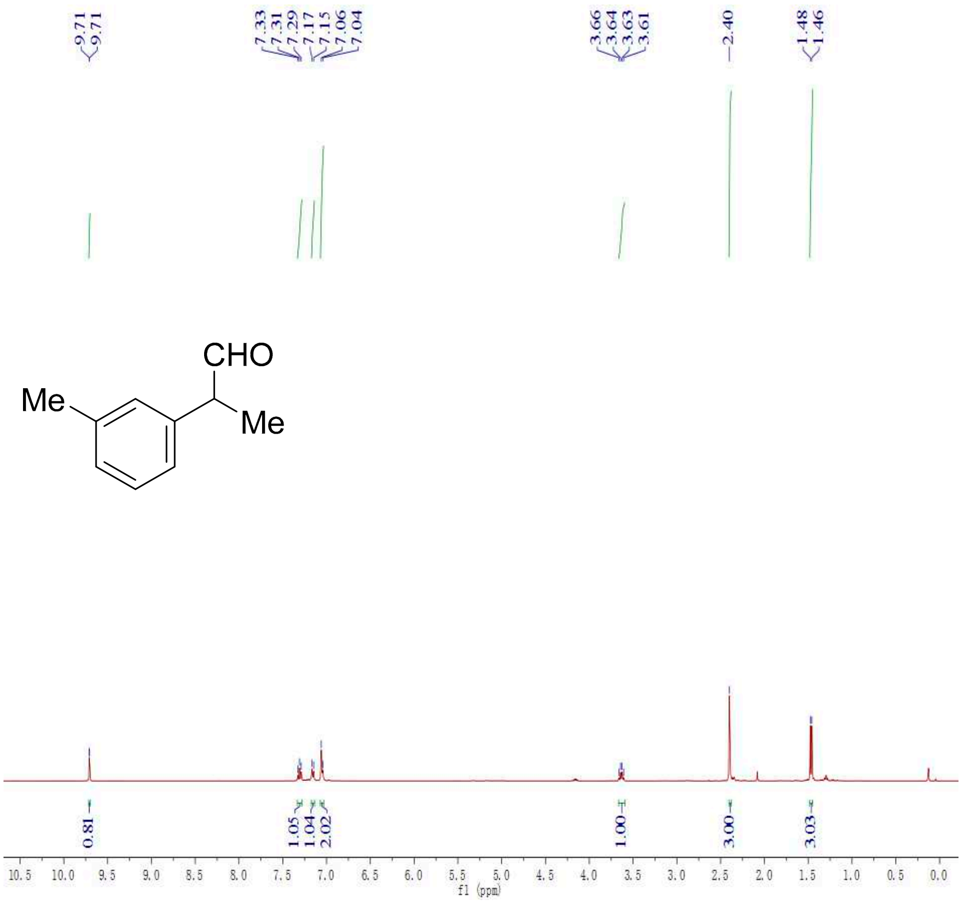

Fig. S44 ^{1}H NMR spectrum of **2m** (400 MHz, $CDCl_3$)

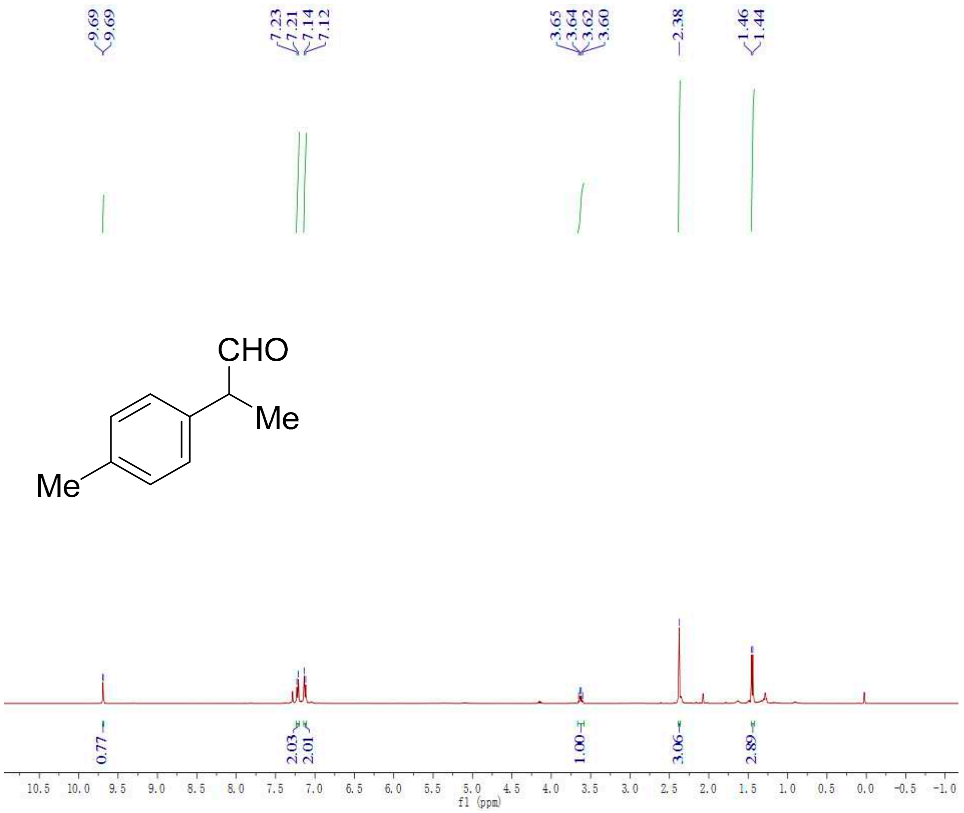

Fig. S45 ^{1}H NMR spectrum of **2n** (400 MHz, $CDCl_3$)

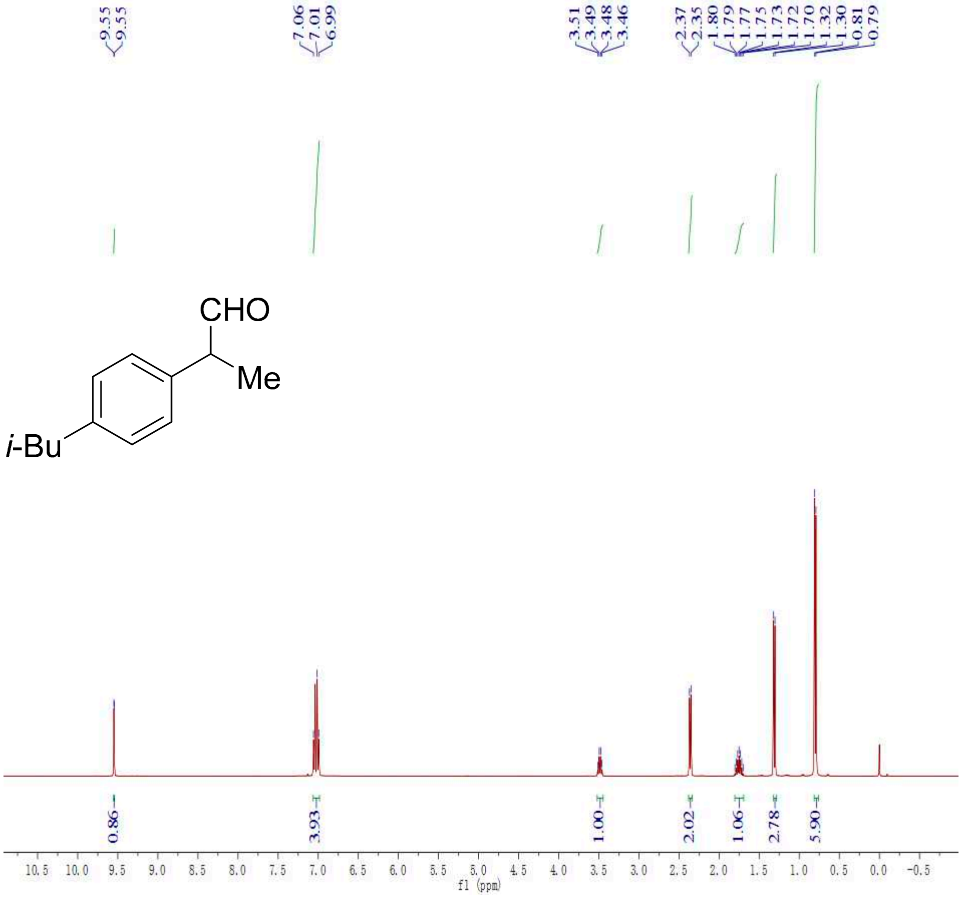

Fig. S46 ^{1}H NMR spectrum of **2o** (400 MHz, $CDCl_3$)

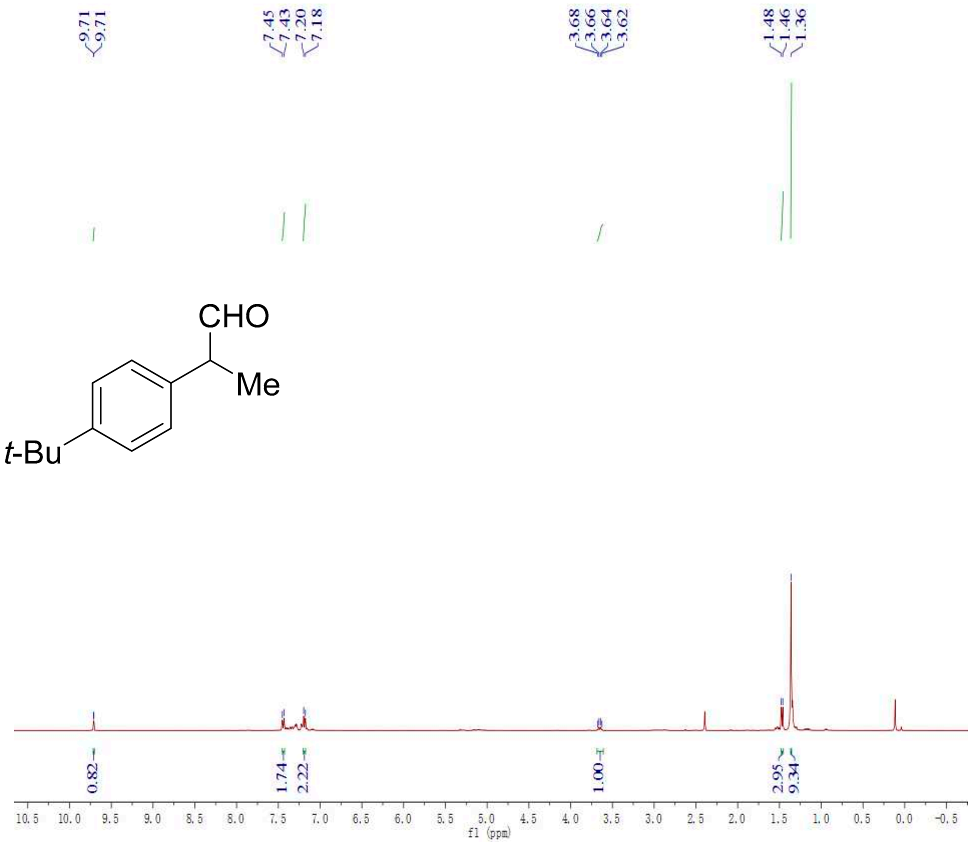

Fig. S47 ^{1}H NMR spectrum of **2p** (400 MHz, $CDCl_3$)

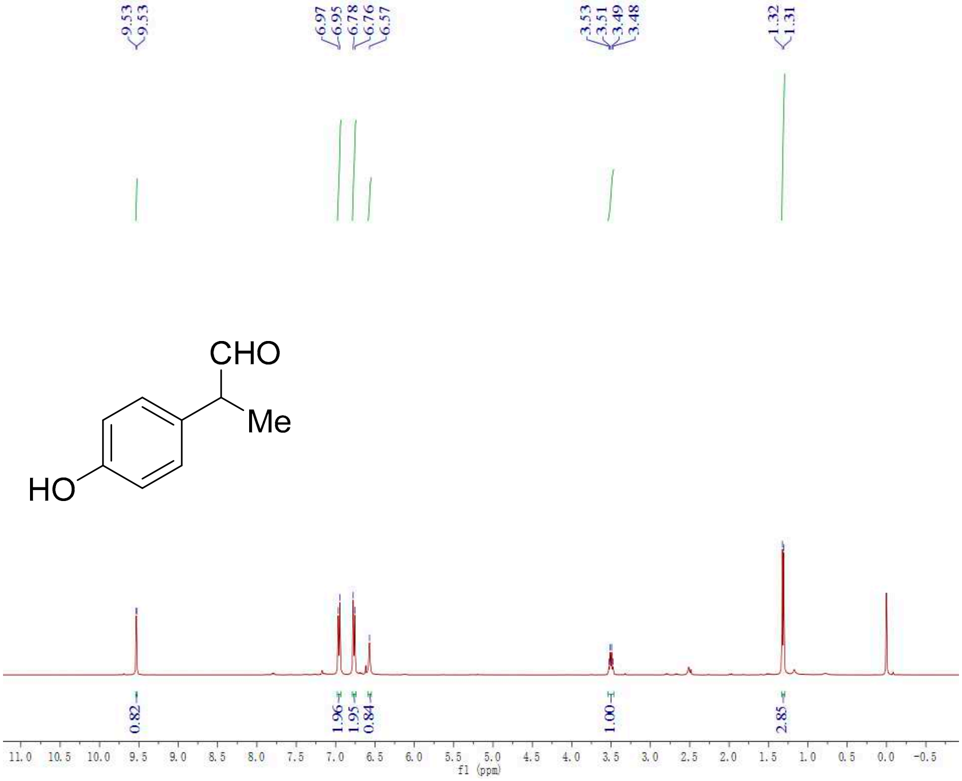

Fig. S48 ^{1}H NMR spectrum of **2q** (400 MHz, $CDCl_3$)

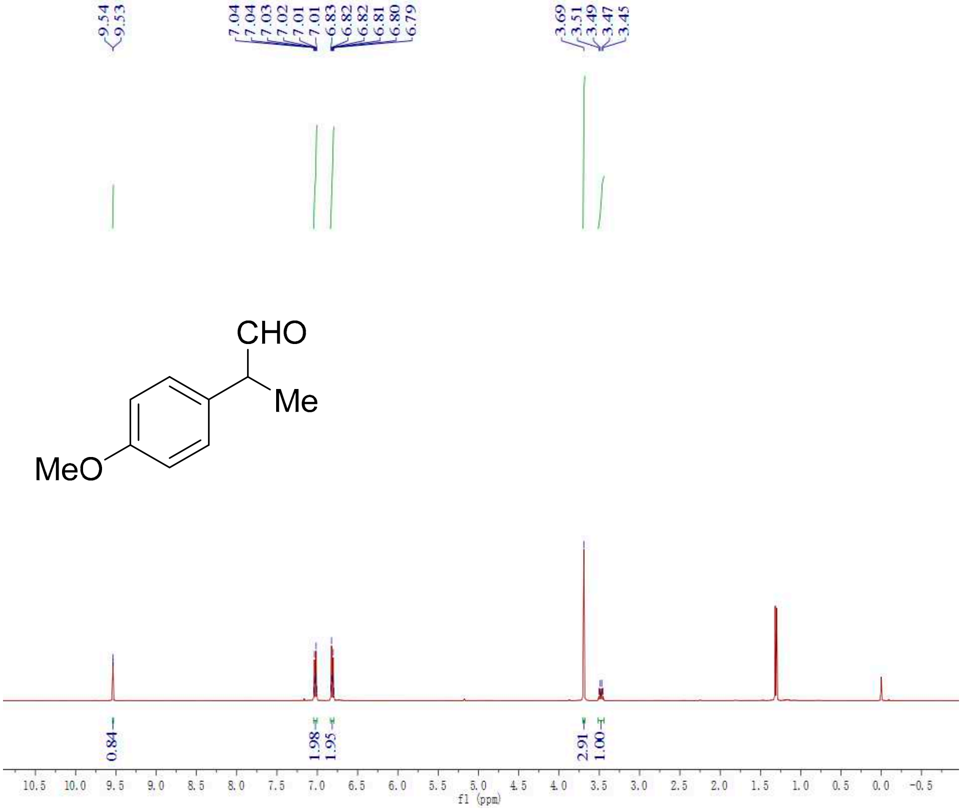

Fig. S49 ^{1}H NMR spectrum of **2r** (400 MHz, $CDCl_3$)

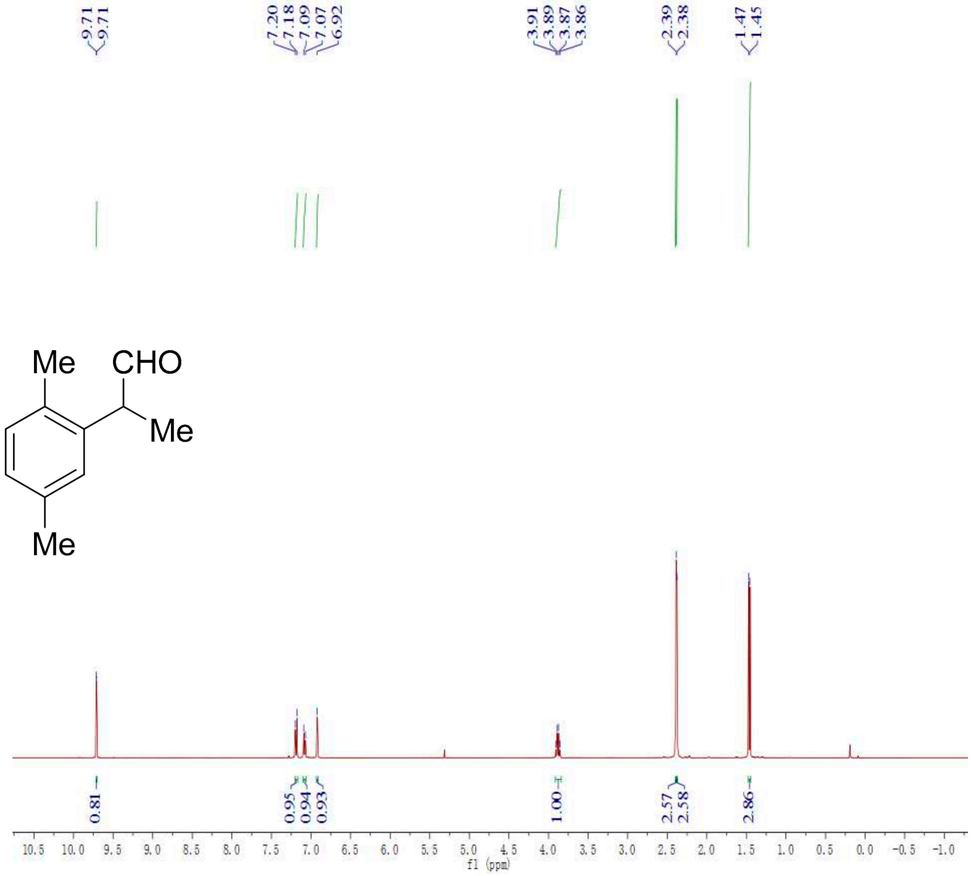

Fig. S50 ^{1}H NMR spectrum of **2s** (400 MHz, $CDCl_3$)

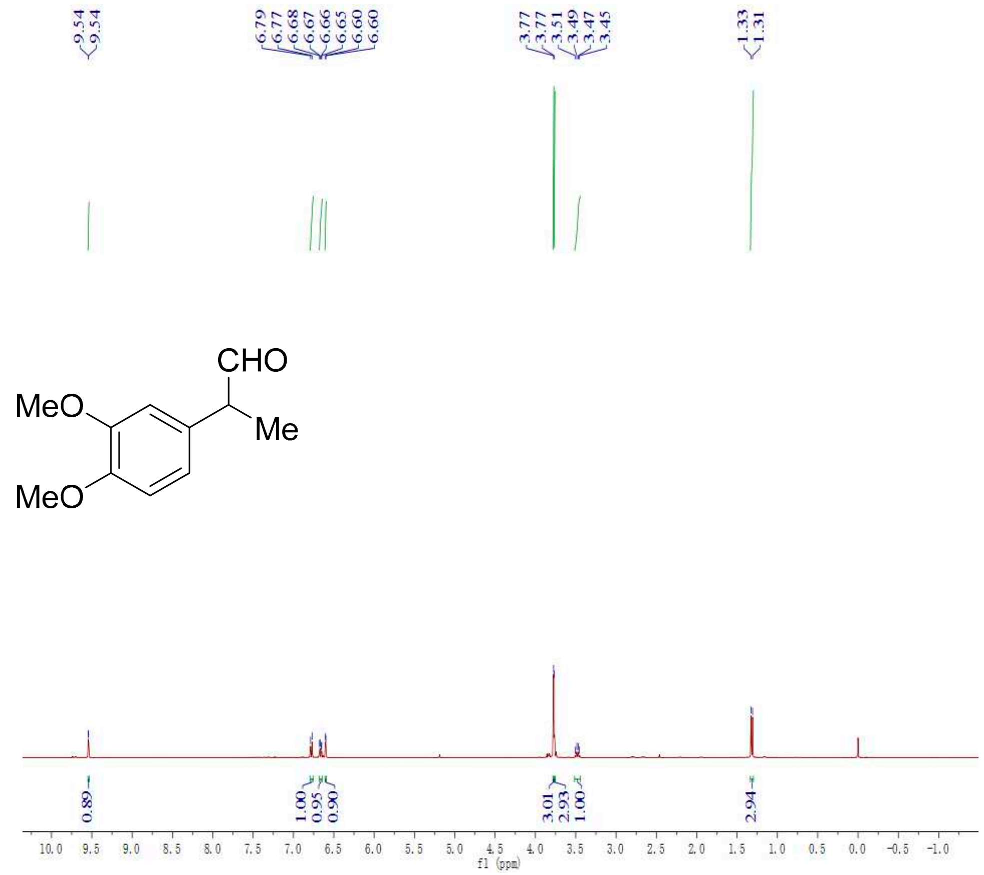

Fig. S51 ^{1}H NMR spectrum of **2t** (400 MHz, $CDCl_3$)

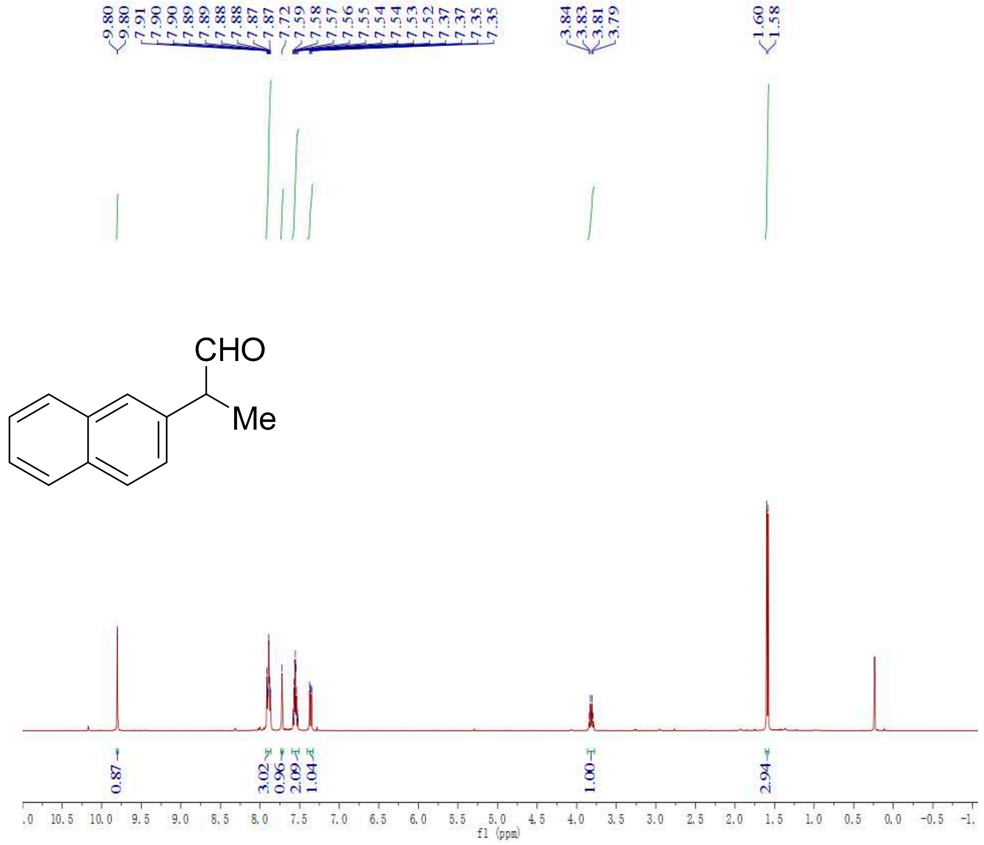

Fig. S52 ^{1}H NMR spectrum of **2u** (400 MHz, $CDCl_3$)

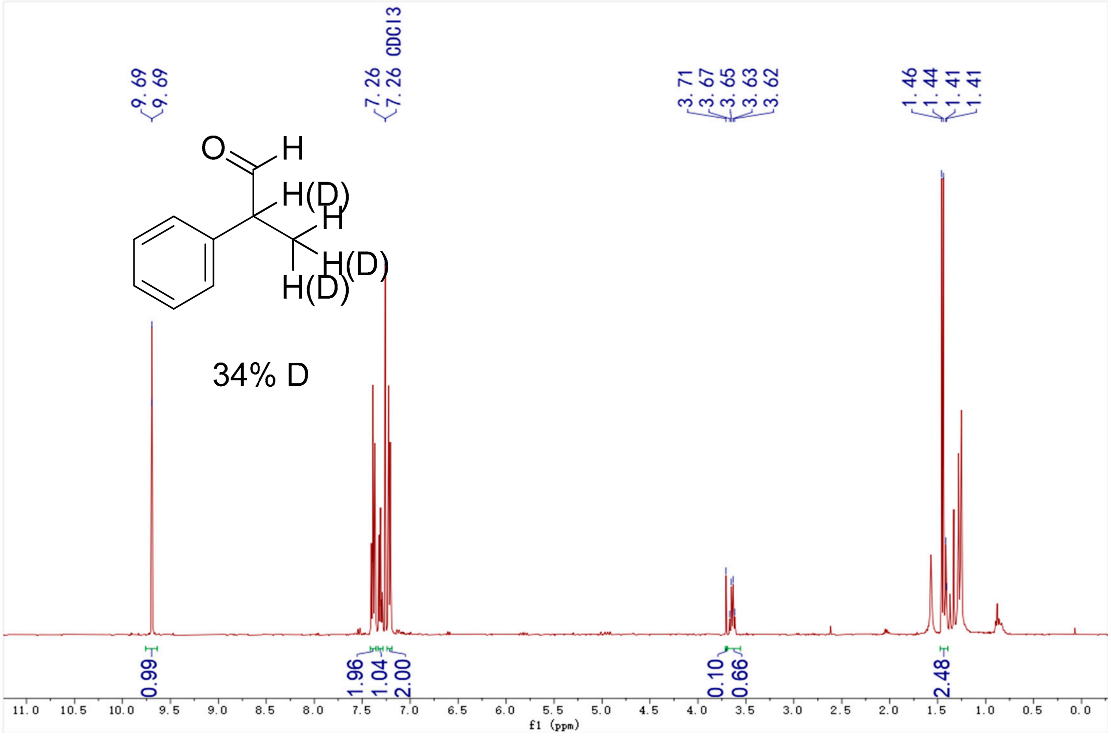

Fig. S53 ^{1}H NMR spectrum of **2a/2a$^{\mathbf{D}}$** (400 MHz, $CDCl_3$)

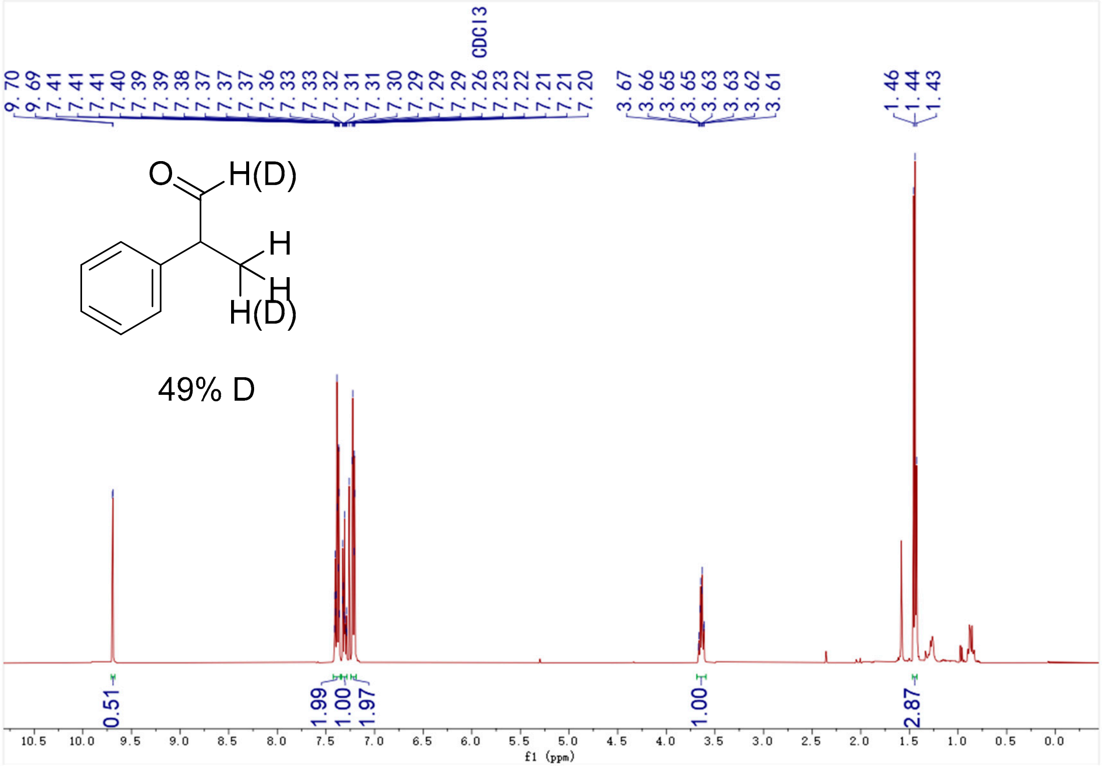

Fig. S54 ^{1}H NMR spectrum of **2a**$^{\underline{\mathbf{D'}}}$ (49% D, 400 MHz, $CDCl_3$)

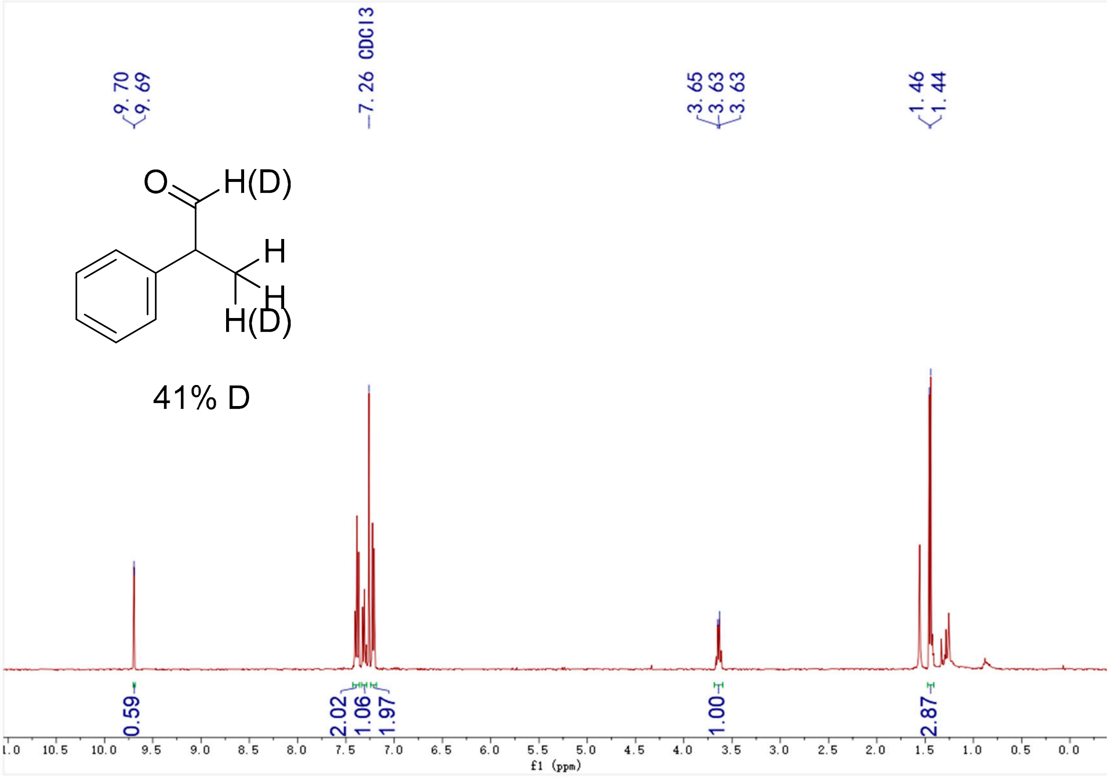

Fig. S55 ^{1}H NMR spectrum of **2a$^{D'}$** (41% D, 400 MHz, $CDCl_3$)
